# C-Terminal Arginine-Selective Cleavage of Peptides as a Method for Mimicking Carboxypeptidase B

**DOI:** 10.1021/acs.orglett.3c02418

**Published:** 2023-08-16

**Authors:** Lyndsey C. Prosser, John M. Talbott, Rose P. Garrity, Monika Raj

**Affiliations:** Department of Chemistry, Emory University, Atlanta, Georgia 30322, United States

## Abstract

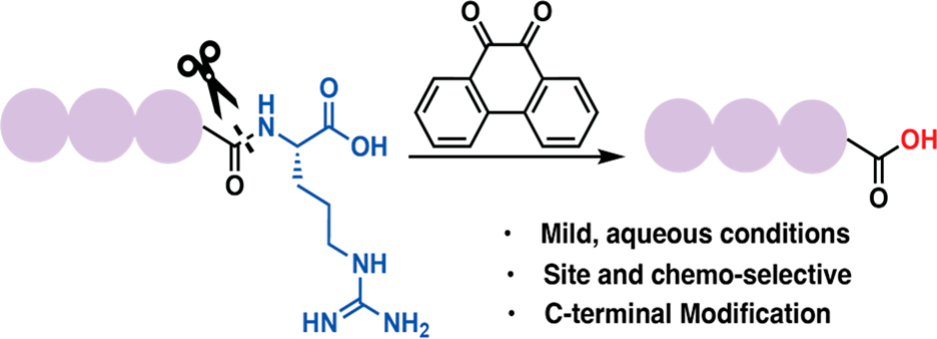

C-Terminal residues play a pivotal role in dictating the structure and functions of proteins. Herein, we report a mild, efficient, chemoselective, and site-selective chemical method that allows for precise chemical proteolysis at C-terminal arginine dictated by 9,10-phenanthrenequinone independent of the remaining sequence. This biomimetic approach also exhibits the potential to synthesize C-terminal methyl ester (−CO_2_Me) peptides.

The use of proteases for cleavage of the amide backbone has aided in the analysis of chemical sequences and determining the impact of the primary sequence on the bioactivity of peptides and proteins.^1^ The significance of determining the primary sequence of proteins prompted the development of several chemical methods that enable cleavage of peptides and proteins at particular amino acid residues, such as Ser/Thr,^2^ Met,^3^ Asp/Glu,^4^ and Asn/Gln.^5^ Most current chemical methods are highly chemoselective and can cleave specific residues anywhere within the peptide/protein sequence.^2−5^ Additionally, a few chemical methods are highly site-selective and can cleave any amino acid residue at the N terminus, regardless of the N-terminal amino acid residue.^6^ However, chemical methods that enable the cleavage of the peptide backbone chain that are both chemoselective and site-selective are lacking. The site-specific cleavage of C-terminal amide bonds in peptides and proteins is an important chemical transformation with multiple applications, including proteomics, site-specific functionalization, and the design of peptide therapeutics with novel functions.^7^ C termini can also serve as a unique signature for biological activities, such as protein localization and the formation of complexes.^8^ For example, the norovirus capsid protein contains a cluster of arginine residues at the C terminus. Previous work has shown that the cleavage of the C-terminal arginine clusters resulted in the loss of activity of the protein.^8^^d^ C-terminal analysis can also aid in the identification of low-abundant proteins, which has become an obstacle for traditional proteomics analysis, thus making selective C-terminal cleavage a point of interest in recent years.^9^ Carboxypeptidase B selectively cleaves peptide bonds at C-terminal lysine and arginine residues and has previously been used in deep screening of C-terminome.^10^ However, chemical methods for selective C-terminal cleavage at specific residues are completely lacking. Herein, we report the first chemical method for selective cleavage of the amide backbone chain at C-terminal Arg residues under mild, aqueous conditions. This reaction is not only chemoselective toward Arg but also highly site-specific for cleaving the peptide backbone with Arg at the C terminus. Although aldehyde formation was observed, no amide backbone cleavage occurred with internal Arg or N-terminal Arg.

We envisioned an Arg-selective cleavage of the peptide bond initiated by chemoselective coupling of 9,10-phenanthrenequinone to the guanidinium moiety of Arg (Figure 1A). The nucleophilic attack of the guanidinium group to 9,10-phenanthrenequinone would generate a coupled product (intermediate **A**, Figure 1A), which in the presence of a base would cleave to a l-glutamate γ-semialdehyde intermediate (intermediate **B**, Figure 1A) along with the formation of a fluorophore byproduct (Figure 1A).^11^ We hypothesized that the amide backbone would then react with the side chain of l-glutamate γ-semialdehyde to generate a pyrrolinium-like intermediate (intermediate **C**, Figure 1A), resulting in the activation of the amide backbone, which would undergo hydrolysis under basic conditions, resulting in the cleavage of the peptide backbone chain to carboxylic acid (**2**, Figure 1A). This is the first chemical strategy ever reported for the selective cleavage of the amide bonds at the Arg residue.

**Figure 1:**
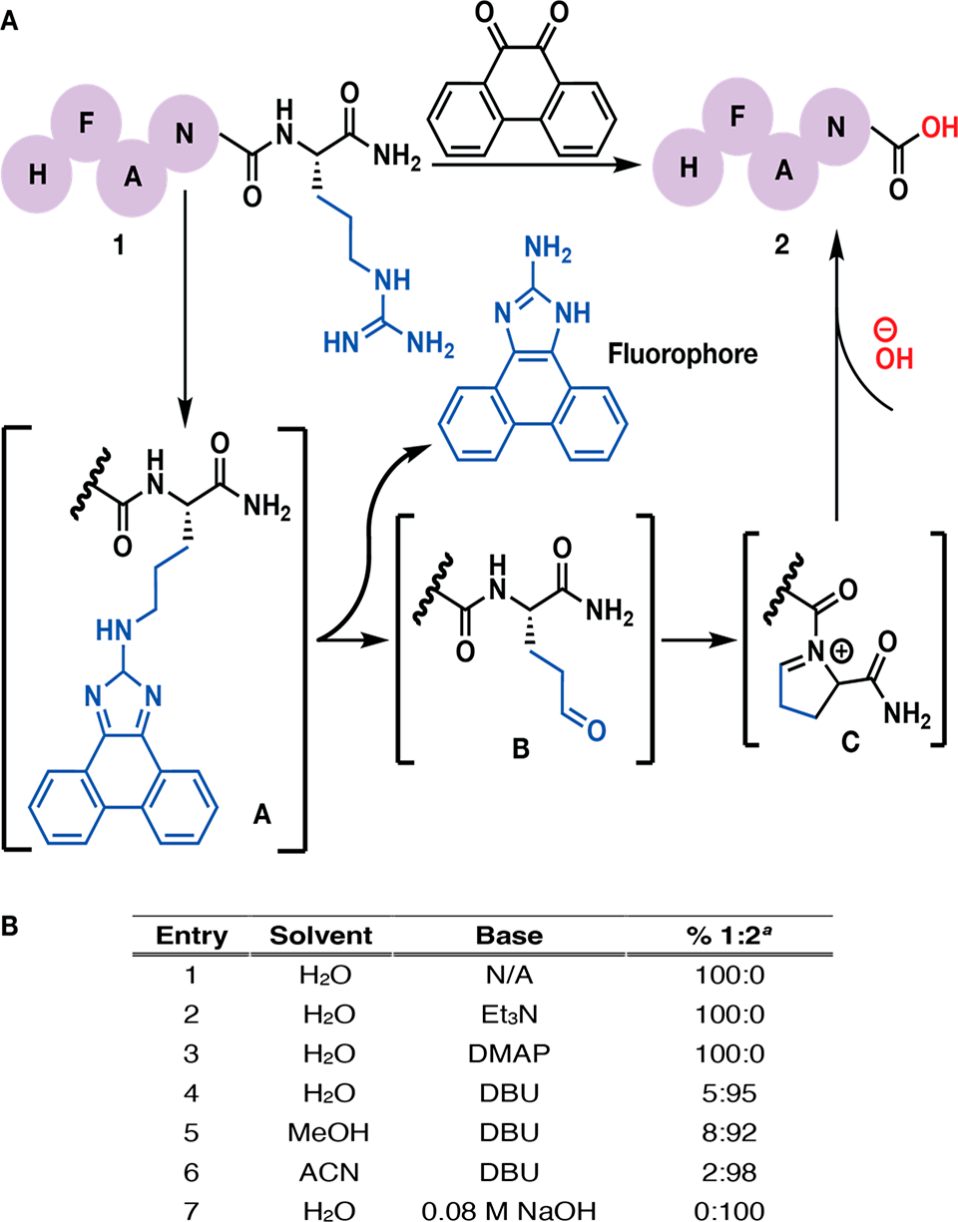
(A) Proposed mechanistic pathway for C-terminal arginine cleavage. (B) Optimization of the cleavage of peptide **1a**. All reactions were set at 1 mg of compound **1a** with 3 equiv of 9,10-phenanthrenequinone in 1 mL (9:1 H_2_O/ACN) and stirred at 37 °C for 3 h. ^*a*^Percent conversion to product **2** was determined by HPLC and LCMS (Supplementary Figure 1 of the Supporting Information).

To optimize the designed reaction, we conducted an initial study with a model substrate, dansyl-HFANR-CONH_2_ (**1a**). A dansyl group was added to the peptide to increase the ultraviolet (UV) absorbance of the peptide for easy analysis by high-performance liquid chromatography (HPLC) because 9,10-phenanthrenequinone and the fluorophore byproduct (Figure 1A) are highly UV-active and could potentially overshadow the peptide.^12^ We initiated the study by incubating the model substrate **1a**, with 9,10-phenanthrenequinone in 9:1 H_2_O/ACN at 37 °C for 3 h but did not observe the formation of any product under the reaction conditions (entry 1, Figure 1B and Supplementary Figure 1 of the Supporting Information). Next, we screened various mild bases, such as 4-dimethylaminopyridine (DMAP) and Et_3_N, but still did not observe the formation of any intermediate or product under the reaction conditions (entries 2 and 3, Figure 1B and Supplementary Figure 1 of the Supporting Information).

To our delight, the addition of 1,8-diazabicyclo[5.4.0]undec-7-ene (DBU) promoted the cleavage of a peptide backbone at C-terminal Arg in both aqueous and organic solvents, such as acetonitrile (ACN) and MeOH (entries 4–6, Figure 1B and Supplementary Figure 1 of the Supporting Information), but the HPLC spectra were difficult to analyze because DBU coeluted with the model peptide substrate **1a** (Supplementary Figure 1 of the Supporting Information).

Next, we switched an inorganic base, NaOH, and utilized varying concentrations, such as 2, 1, and 0.5 M, of NaOH solutions. Although we observed the desired cleavage of amide bonds at the Arg C terminus, we also observed the hydrolysis of the amide side chain of Asn to Asp. With further optimization of the NaOH concentration, we observed that 0.08 M NaOH is ideal for cleaving Arg at the C terminus without any modification of other side chains (entry 7, Figure 1B and Supplementary Figure 1 of the Supporting Information). When the concentration of NaOH was further lowered to 0.02 M, we observed the formation of l-glutamate γ-semialdehyde intermediate **B** as analyzed by liquid chromatography–mass spectrometry (LCMS) (Supplementary Figure 1 of the Supporting Information). The reaction was found to work in various aqueous and organic solvents, including ACN and MeOH. However, the use of anhydrous solvents resulted in the attachment of 9,10-phenanthrenequinone to Arg and generated intermediate **A**, as analyzed by LCMS (Supplementary Figure 1 of the Supporting Information).

From the above study, we concluded that the optimal reaction conditions for the C-terminal arginine cleavage involve incubation with 3 equiv of 9,10-phenanthrenequinone in 0.08 M NaOH solution in 9:1 H_2_O/ACN at 37 °C for 3 h. A model dipeptide dansyl-HR-CONH_2_ was cleaved, isolated, and characterized by ^1^H and ^13^C nuclear magnetic resonance (NMR) and high-resolution mass spectrometry (HRMS) analyses (Supplementary Figure 2 of the Supporting Information). The fluorophore byproduct was also isolated and confirmed by ^1^H and ^13^C NMR and HRMS analyses (Supplementary Figure 3 of the Supporting Information).^11^

To determine the chemoselectivity of this approach toward Arg residues, we tested the optimized conditions on other peptides, dansyl-XHG-CO_2_H, containing reactive amino acids, where X = Cys, Asp, Lys, Met, Ser, Trp, and Tyr. No modification of any other amino acid was observed under the reaction conditions (Supplementary Figure 4 of the Supporting Information). Next, we tested the optimized conditions on peptides containing Arg at positions other than the C terminus [H_2_N-RHYK(dansyl)FA-CONH_2_, dansyl-RHQL-CONH_2_, and dansyl-MEHFRWGKPV-CONH_2_]. Surprisingly, no cleavage of the peptide backbone was observed when Arg was not located at the C terminus. However, we observed the formation of l-glutamate γ-semialdehyde intermediate **B** with these peptides under the reaction conditions (Supplementary Figure 5 of the Supporting Information).

To further understand the unique site selectivity of this method for C-terminal arginine, we investigated the impact of the neighboring residue on the efficiency of the cleavage, by synthesizing peptides dansyl-HFAXR-CONH_2_ (**1a**–**1f**) with different amino acids at the C + 1 position, including a negative-charged residue (X = Asp), a positive-charged residue (X = His), polar residues (X = Ser or Asn), and hydrophobic and bulky residues (X = Val and Trp).

We subjected these peptides to the optimized cleavage conditions and observed the full cleavage of C-terminal Arg in all cases, regardless of the nature of the neighboring C + 1 residue and without any modification of the side chain of any of these reactive amino acids (entries 1–6, Figure 2 and Supplementary Figure 6 of the Supporting Information). Additionally, no difference in the cleavage efficiency of the C-terminal Arg residue was observed with peptides comprising carboxylic acid at the C terminus (entry 7, Figure 2 and Supplementary Figure 6 of the Supporting Information) or a free N terminus (entry 8, Figure 2 and Supplementary Figure 6 of the Supporting Information). Intrigued by the high chemoselectivity and site selectivity of this cleavage method, we conducted the reaction on a peptide containing consecutive Arg residues at the C terminus, dansyl-HKRR-CONH_2_ (**1i**). Notably, we observed the cleavage of both Arg residues consecutively using higher equivalents of 9,10-phenanthrenequinone under the optimized reaction conditions (entry 9, Figure 2 and Supplementary Figure 6 of the Supporting Information). These studies further confirmed the high chemoselectivity and site selectivity of this cleavage protocol.

**Figure 2:**
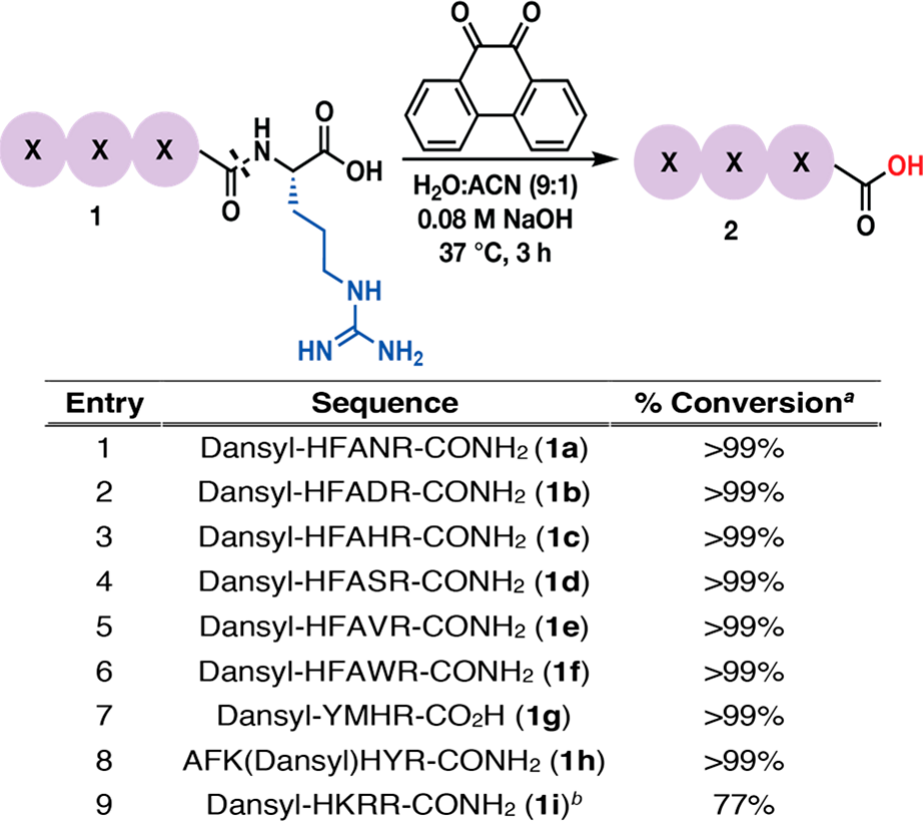
Peptide scope for C-terminal arginine cleavage using the optimized conditions. ^*a*^Percent conversion to product **2** was determined by HPLC and LCMS (Supplementary Figure 6 of the Supporting Information). ^*b*^A total of 6 equiv of 9,10-phenanthrenequinone.

Given the robustness for site selectivity of solely C-terminal arginine cleavage, we decided to investigate the mechanistic pathway using density functional theory (DFT) calculations [B3LYP/6-311+G(d,p)]. The computational studies on peptide aldehyde intermediates containing Arg at the C terminus and in the middle of the chain, H_3_N^+^-GR(CHO)G-CO_2_^–^ and H_3_N^+^-GGR(CHO)-CO_2_^–^, and corresponding pyrrolinium-like intermediates revealed that the energy required for the cleavage of C-terminal Arg is significantly lower (∼16 kcal/mol) compared to when Arg was not present at the C terminus (Figure 3A and Supplementary Figure 7 of the Supporting Information). Given that the terminal position in the model peptide used for these calculations is Gly, the residue with the least steric impact, we hypothesized that other residues would result in substantially starker differences, because they will increase the steric interactions and could inhibit the necessary cyclization. This observation is similar to that of terminal serine cleavage in peptides and proteins by serine proteases that have been reported in previous precedent.^13^ On the basis of these calculations and observed experimental results with varying peptides, we hypothesized that the site selectivity of Arg cleavage was most likely due to the lower steric hindrance to the formation of C-terminal five-membered pyrrolinium-like intermediate (intermediate **C**) compared to when Arg is present at other positions on the peptide. In agreement with the computational analysis, peptides with internal or N-terminal arginine resulted in only the formation of l-glutamate γ-semialdehyde intermediate **B**.

**Figure 3:**
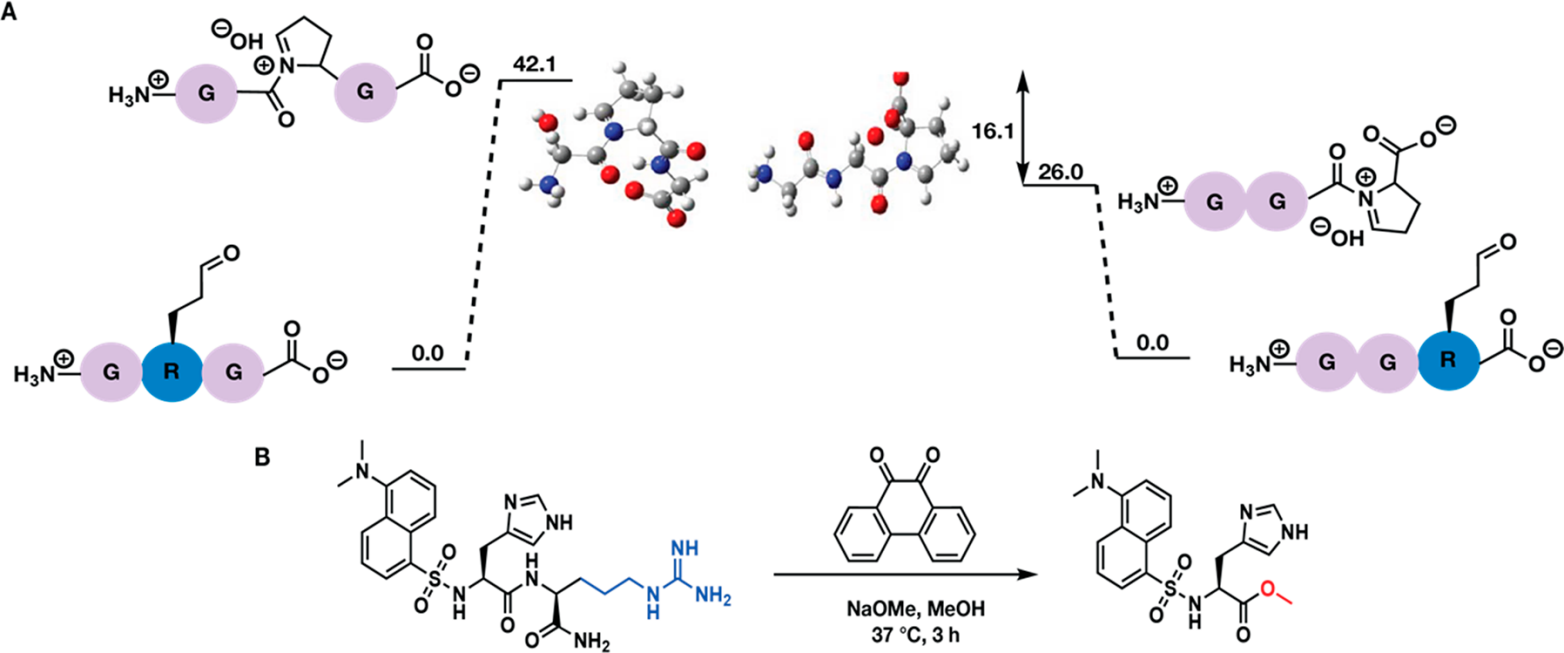
Mechanistic investigations: (A) DFT computational analysis at the B3LYP/6-311+G(d,p) theory and basis set for geometry optimization and frequency calculations for peptide aldehydes H_3_N^+^-GR(CHO)G-CO_2_^–^ and H_3_N^+^-GGR(CHO)-CO_2_^–^ and pyrrolinium-like intermediates. Energy differences (kcal/mol) are the difference between the aldehyde and pyrrolinium-like intermediates. The gray, white, red, and blue balls represent the carbon, hydrogen, oxygen, and nitrogen atoms, respectively. (B) Formation of methyl ester on model dipeptide dansyl-HR-CONH_2_.

We concluded that, when Arg is on the C terminus, the amide of the peptide backbone chain can carry out a nucleophilic attack onto the aldehyde intermediate **B**, generating the five-membered pyrrolinium-like intermediate **C** through dehydration (Figure 1A). Typically, amides are not strong electrophiles as a result of the formation of the resonating structure with the carbonyl via delocalization of the lone pairs of the amide nitrogen.^14^ However, the formation of pyrrolinium with the backbone amide leads to the activation and twisting of the amide bond, so that it is no longer able to form resonating structures with the carbonyl group, thus reducing its double-bond character and making it more susceptible to nucleophilic attack.^15^ The reaction of the hydroxide ion with the activated amide results in the cleavage of the arginine residue, along with the formation of a new C-terminal carboxylic acid on the peptide.

To find more supporting evidence for the proposed mechanism, we planned to trap the activated amide with a different nucleophile. We conducted the reaction on a model dipeptide dansyl-HR-CONH_2_ and incubated it with 9,10-phenanthrenequinone in anhydrous methanol with the addition of NaOMe as the base and nucleophile and left the reaction at 37 °C for 3 h. As expected, we observed the cleavage of C-terminal Arg along with the formation of the corresponding methyl ester, dansyl-H-CO_2_Me, as confirmed by ^1^H and ^13^C NMR and HRMS (Figure 3B and Supplementary Figure 8 of the Supporting Information). This observation supports the hypothesis of the formation of an activated amide and the requirement of a nucleophilic attack on the activated amide for the cleavage.

In summary, we report the development of a chemoselective and site-specific chemical proteolysis method, mimicking carboxypeptidase B, that enables the cleavage of C-terminal Arg with 9,10-phenanthrenequinone through the hydrolysis of the activated amide under basic conditions. On the basis of the computational calculations and experimental data, we concluded that the high C-terminal selectivity is due to the lower energy of the activated amide intermediate if Arg is present at the C terminus compared to any other position. The method exhibits a high substrate scope independent of the amino acid residues on the peptide. This method also exhibits the potential to generate C-terminal diversified peptides with varying functional groups, such as esters, thioesters, and amides.

## Data Availability

The data underlying this study are available in the published article and its Supporting Information.

## Supporting Information Available

General experimental procedures, computational calculations, and characterization details, including HPLC, HRMS, and ^1^H and ^13^C NMR spectra of all compounds (PDF)
FAIR data, including the primary NMR FID files, for all the three compounds reported in the manuscript (ZIP)
