# Correction to “C-Terminal Arginine-Selective Cleavage of Peptides as a Method for Mimicking Carboxypeptidase B”

**DOI:** 10.1021/acs.orglett.3c02915

**Published:** 2023-10-04

**Authors:** Lyndsey C. Prosser, John M. Talbott, Rose P. Garrity, Monika Raj

Figure 1A was published with an incorrect structure for “Intermediate A”. We are missing the double bond. We have corrected the structure to display the proper imine.

**Figure 1:**
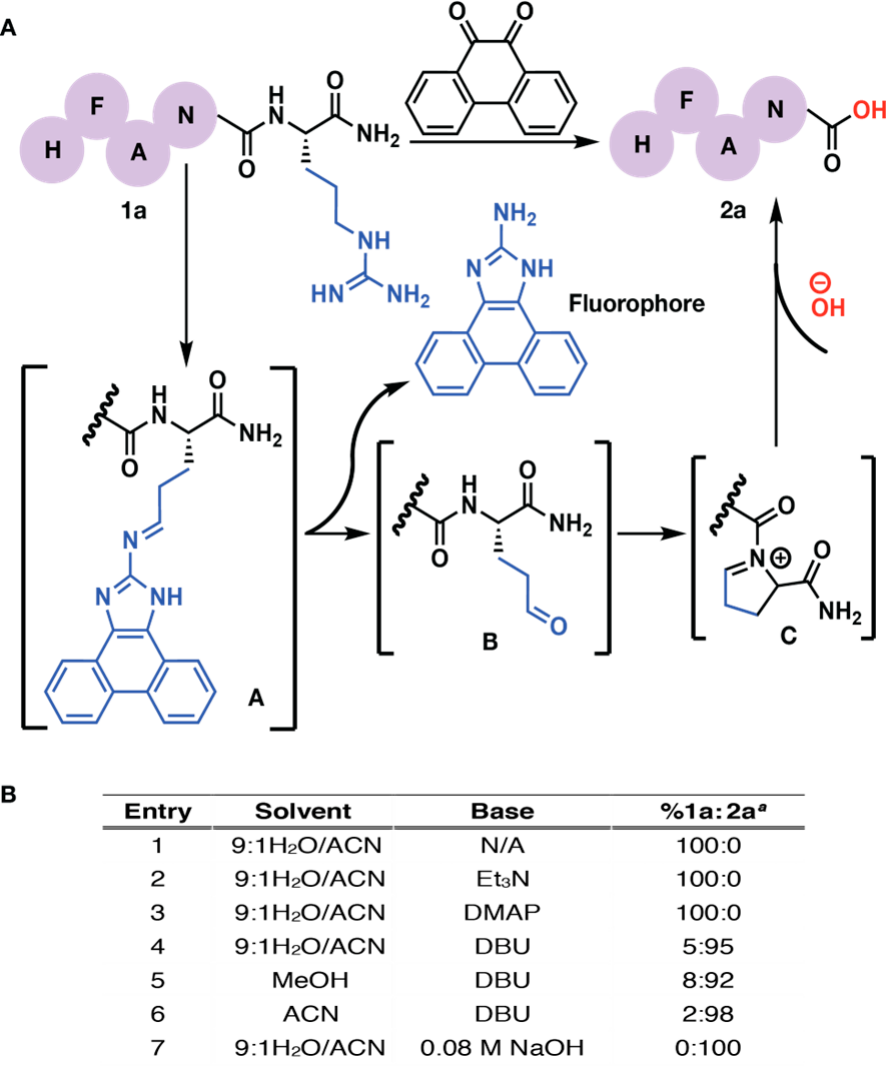
(A) Proposed mechanistic pathway for C-terminal arginine cleavage. (B) Optimization of the cleavage of peptide **1a**. All reactions were set at 1 mg of compound **1a** with 3 equiv of 9,10-phenanthrenequinone in 1 mL of solvent and stirred at 37 °C for 3 h. ^*a*^Percent conversion to product **2a** was determined by HPLC and LCMS (Supplementary Figure 1 of the Supporting Information).

Page 2, column 2, first paragraph: “However, the use of anhydrous solvents resulted in the attachment of 9,10-phenanthrenequinone to Arg and generated intermediate A, as analyzed by LCMS (Supplementary Figure 1 of the Supporting Information)” should be rephrased to “However, the use of anhydrous solvents resulted in the attachment of 9,10-phenanthrenequinone to Arg, which was generated by the reduction of imine intermediate **A**, purified by HPLC and analyzed by high-resolution mass spectrometry (HRMS) analysis (Supplementary Figure 1 of the Supporting Information). DBU has been reported as a reducing agent and exhibits the potential to reduce imine intermediate **A**.^9b,c^ We have further confirmed the formation of reduced imine intermediate **A** in the presence of DBU by carrying out reactions with other peptides (Supplementary Figure 1 of the Supporting Information).

We add the following new refs 9b,c and 12b:

(9) (b) NyoniD.; LobbK. A.; KayeP. T.; CairaM. R.DBU-mediated cleavage of aryl- and heteroaryl disulfides.ARKIVOC2012, 2012, 245–252. 10.3998/ark.5550190.0013.623. (c) XuS.; YangF.; FanH.; ZhaoX.; XuY.; WangS.; ZhangX.1,8-Diazobicyclo[5.4.0]undec-7-ene (DBU)-promoted reduction of azides to amines under metal-free conditions.New J. Chem.2022, 46, 9994−9998. 10.1039/D2NJ00341D

(12) (b) KemnitzC. R.; LoewenM. J.Amide resonance correlates with a breadth of C-N rotation barriers. J. Am. Chem. Soc.2007, 129, 2521−2528. 10.1021/ja066302417295481

Reference numbers were changed in the revised manuscript as follows.

Page 1:

1. “such as protein localization and the formation of complexes.^8^” should be changed to “such as protein localization and the formation of complexes.^7,8^”

2. “C-terminal arginine clusters resulted in the loss of activity of the protein.^8d^” should be changed to “C-terminal arginine clusters resulted in the loss of activity of the protein.^8^”

3. “C-terminal cleavage a point of interest in recent years.^9^” should be changed to “C-terminal cleavage a point of interest in recent years.^8^”

4. “been used in deep screening of C-terminome.^10^” should be changed to “been used in deep screening of C-terminome.^8^”

5. “along with the formation of a fluorophore byproduct (Figure 1A).^11^” should be changed to “along with the formation of a fluorophore byproduct (Figure 1A).^9^”

Page 2:

6. “overshadow the peptide.^12^” should be changed to ”overshadow the peptide.^9^”

7. “HRMS analyses (Supplementary Figure 3 of the Supporting Information).^11^” should be changed to “HRMS analyses (Supplementary Figure 3 of the Supporting Information).^9−11^”

Page 3:

8. “serine proteases that have been reported in previous precedent.^13^” should be changed to “serine proteases that have been reported in previous precedent.^11^”

9. “via delocalization of the lone pairs of the amide nitrogen.^14^” should be changed to “via delocalization of the lone pairs of the amide nitrogen with the carbonyl group of the amide.^12,13^”

10. “and making it more susceptible to nucleophilic attack.^15^” should be changed to “and making it more susceptible to nucleophilic attack.^14,15^”

Rewording of sentences:

Page 1, column 2:

11. “resulting in the cleavage of the peptide backbone chain to carboxylic acid (**2**, Figure 1A).” should be changed to “resulting in the cleavage of the peptide backbone chain to carboxylic acid (**2a**, Figure 1A).”

Page 2, column 2:

We would like to add the following after “(Supplementary Figure 4 of the Supporting Information)”. “Due to subpar solubility of 9,10-phenanthrenequinone in water, the solvent system was adjusted to 6:1 H_2_O/ACN.”

Page 3, column 2:

13. “Typically, amides are not strong electrophiles as a result of the formation of the resonating structure with the carbonyl via delocalization of the lone pairs of the amide nitrogen.” should be changed to “Typically, amides are challenging to cleave due to the formation of the resonating structure with the carbonyl via delocalization of the lone pairs of the amide nitrogen with the carbonyl group of amide.^12,13^”

Figure 2: The only change is on the arrow from (9:1) to (6:1).
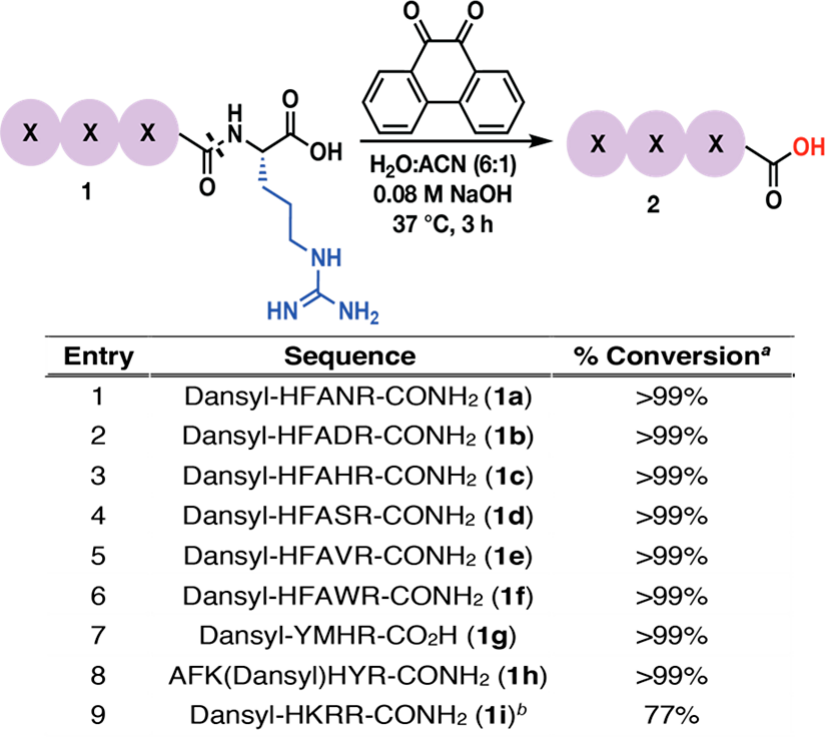


The Supporting Information has been corrected to include “DBU in anhydrous ACN with peptide **1e**” (HPLC and HRMS data added) and “No Base in ACN with free N-terminal Arg peptide” (HPLC and HRMS data added).

## Supporting Information Available

General experimental procedures, computational calculations, and characterization details, including HPLC, HRMS, and ^1^H and ^13^C NMR spectra of all compounds (PDF)

