# Supplementary material for: C-Terminal Arginine-Selective Cleavage of Peptides as a Method for Mimicking Carboxypeptidase B
Source: Org Lett. 2023 Aug 16;25(33):6206–10. doi: 10.1021/acs.orglett.3c02418 (PMC10463270; doi:10.1021/acs.orglett.3c02418)
Supplement: Supplementary file 1 — ol3c02418_si_001.pdf [file ol3c02418_si_001.pdf]

Supporting Information

C-terminal Arginine Selective Cleavage of Peptides as a Method for Mimicking Carboxypeptidase B

Lyndsey C. Prosser[1‡], John M. Talbott[1‡], Rose P. Garrity[1], Monika Raj[1*]
[1]Department of Chemistry, Emory University, Atlanta, GA, 30322, US

Table of contents

		Pages
**I.**	General	1
**II.**	Materials	1
**III.**	Purification	1
**IV.**	Analytical Methods	2
**V.**	Fmoc Solid Phase Peptide Synthesis	2
**VI.**	Optimized Cleavage of Peptide **1a**	3-5
**VII.**	Reaction Optimization with Peptide **1a**	6-9
**VIII.**	Characterization of Cleavage Product with Peptide Dansyl-HR-CONH$_2$	10-12
**IX.**	Characterization of fluorophore by-product	13-14
**X.**	Chemoselectivity Studies of Dansyl-XHG-CO$_2$H Peptides	14-23
**XI.**	Non-C-Terminal Arg Peptides	23-28
**XII.**	Cleavage of C+1 Peptides	28-35
**XIII.**	Cleavage of Peptides with free N and C-termini and multiple Arg at C-terminus	36-40
**XIV.**	Computational Data	40-45
**XV.**	Formation of Methyl Ester Peptide	45-47
**XVI.**	References	48

I. General. All commercial materials (Sigma-Aldrich, Oakwood, and Novabiochem) were used without further purification. All solvents were reagent or HPLC (Fisher) grade. All reactions were performed under air in glass vials. Percent conversions were obtained by comparing HPLC peak areas of products and starting materials. HPLC and MS were used to monitor reaction progress, and product elucidation was done using MS and NMR.

II. Materials. Fmoc-amino acids, Rink amide resin, and hexafluorophosphate benzotriazole tetramethyl uronium (HBTU) were obtained from CreoSalus (Louisville, Kentucky). Wang resin was obtained from Sigma Aldrich (St. Louis, Missouri). N,N′-diisopropylethylamine (DIPEA) was obtained from TCI (Portland, Oregon). Piperidine and trifluoroacetic acid (TFA) were obtained from Alfa Aesar (Ward Hill, Massachusetts). N,N-dimethylformamide (DMF), dichloromethane (DCM), methanol (MeOH), and acetonitrile (ACN) were obtained from VWR (100 Matsonford Road Radnor, Pennsylvania). All other small molecules, including 9,10-phenanthrenequinone, were obtained from Sigma.

III. Purification. HPLC: Purification of peptide starting materials was performed using high performance liquid chromatography (HPLC) on an Agilent 1100 series HPLC equipped with a C-18 reverse phase column with a particle size of 5 μm or Teledyne ISCO ACCQ Prep HP150 equipped with a C-18 reverse phase 9.4x250 mm column with a particle size of 5 μm. All separations involved a mobile phase of 0.1 % formic acid in water (solvent A) and 0.1 % formic acid in acetonitrile (solvent B). The

HPLC method used a linear gradient of 5-40% solvent B over 30 min at RT with a flow rate of 1 mL min^{-1}. The eluent was monitored by absorbance at 220 nm and 254 nm.

IV. Instrumentation and sample analysis.

NMR. ^1H and ^{13}C spectra were acquired at 25 °C in CD_3OD or DMSO-d_6 using an Agilent DD2 (400 MHz) spectrometer with a 3-mm He triple resonance (HCN) cryoprobe. All ^1H NMR chemical shifts (δ) were referenced relative to the residual CD_3OD peak at 3.31 ppm or DMSO-d_6 at 2.50 ppm. ^{13}C NMR chemical shifts were referenced to 49.00 ppm for CD_3OD or 39.52 ppm. ^{13}C NMR spectra were proton decoupled. NMR spectral data are reported as chemical shift (integration, multiplicity, coupling constants (*J*)). The following abbreviations (or combinations thereof) were used to explain multiplicities: apparent (app), singlet (s), doublet (d), triplet (t), quartet (q), multiplet (m), broad (b). Coupling constants (J) are reported in hertz (Hz).

Analytical HPLC. Analytical HPLC chromatography (HPLC) was performed on an Agilent 1200 series HPLC equipped with a 5 µm pore size C-18 reversed-phase column. All separations involved mobile phase of 0.1 % formic acid in water (solvent A) and 0.1 % formic acid in acetonitrile (solvent B) run in linear gradients with a constant flow rate of 1 mL min^{-1}. The eluent was monitored with a detection wavelength of 220 nm. **HPLC METHOD A:** Gradient: 2-60% B over 30 min. **HPLC METHOD B:** Gradient: 2-80 % B over 30 min.

HRMS. High resolution MS data were acquired on Thermo Exactive Plus using a heated electrospray source. The solution was infused at a rate of 10-25 µL min^{-1} electrospray using 3.3 kV. The typical settings were Capillary temp 320 °C. S-lens RF level was between 30-80 with an AGC setting of 1 E6. The maximum injection time was set to 50 ms. Spectra were taken at 140,000 resolutions at m/z 200 using Tune software and analyzed with ThermoFischer's Freestyle software.

V. Fmoc Solid-Phase Peptide Synthesis (Fmoc-SPPS).[1] Peptides were synthesized using standard protocols. Peptides were synthesized manually on a 0.25 or 0.40 mmol scale using Rink amide resin or Wang resin. Resin was swollen with DCM for 1 hour at RT. Fmoc was deprotected using 20 % piperidine in DMF for 30 min to obtain a deprotected resin. Fmoc protected amino acid (1.25 mmol or 2.00 mmol, 5 equiv.) was coupled using HBTU (1.25 mmol or 2.00 mmol, 5 equiv.) and DIPEA (1.25 mmol or 2.00 mmol, 5 equiv.) in DMF for 25 min at RT. Fmoc deprotection was achieved using 20% piperidine in DMF for 20 min at RT. N-terminal dansylation was accomplished with Dansyl-Cl (0.75 mmol or 1.2 mmol, 3 equiv.) and Et_3N (0.75 mmol or 1.2 mmol, 3 equiv.) in DMF at RT overnight. Lysine side chain dansylation was achieved using commercially available Fmoc-MTT-lysine which was selectively cleaved using 1.8% TFA in DCM for 3 min at RT (10 x 3 mL), yielding red resin. Resin was washed with 10% DIPEA in DMF to remove color, then Dansyl-Cl and Et_3N were coupled overnight. Peptides were cleaved from the resin using 10 mL of a cocktail consisting of 95:2.5:2.5 trifluoroacetic acid : water : triethylsilane for 2 hours. The resin was removed by filtration and the resulting solution was concentrated via air. Peptides were precipitated and centrifugated with cold diethyl ether (3 x 10 mL) to obtain the crude product. Crude peptides were dissolved in ACN:H_2O and purified by preparatory HPLC.

VI. Supplementary Figure 1. Optimization of C-terminal Arginine Cleavage using peptide 1a

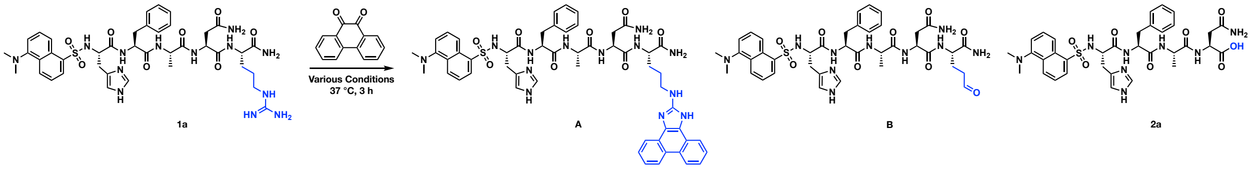

General Procedure: Dansyl-HFANR-$CONH_2$ (**1a**) (1 mg, 1.14 µmol, 1 equiv.) was dissolved in 500 µL solvent H_2O in a 1/2" dram vial. Next, 9,10-phenanthrenequinone (0.71 mg, 3.43 µmol, 3 equiv.) from a freshly prepared stock solution in ACN was added in one portion before 258 µL of solvent H_2O was added to reach 900 µL total volume. For reaction utilizing organic bases (DMAP, DBU, or Et_3N), 30 equiv. was added along with 100 µL of H_2O yielding the final 1 mL volume. For reactions utilizing NaOH as the base, 0.8 M NaOH (100 µL) was added from a freshly prepared stock solution yielding a total volume of 1 mL with a concentration of 0.08 M NaOH. The concentration of the peptide in solution was 1.14 mM. The vial was stirred at 37 °C for 3 h. The solution was filtered via syringe filtration then analyzed via **HPLC method A** to determine the percent conversion to **2a**.

Entry	Solvent	Base	% 1a:A:B:2a
**1**	H_2O	N/A	100:0:0:0
**2**	H_2O	Et_3N	100:0:0:0
**3**	H_2O	DMAP	100:0:0:0
**4**	H_2O	DBU	5:0:0:95[a]
**5**	MeOH	DBU	8:0:0:92[a]
**6**	ACN	DBU	2:0:0:98[a]
**7**	H_2O	0.08 M NaOH	0:0:0:100
**8**	H_2O	0.02 M NaOH	0:0:100:0
**9**	Anhydrous ACN	DBU	0:100:0:0[a]

[a]DBU overlapped with peak for **2a** making accurate percent conversion undeterminable. However, only minimal **1a** is still seen at 11.6 min.

__Please note__: For optimized conditions using 0.08 N NaOH, **HPLC method B** was used for analysis therefore there is difference in the retention time of **1a** and **2a** in the HPLC traces.

Dansyl-HFANR-$CONH_2$ peptide 1a. LCMS *m/z* 438.6989 (calcd. $[(M+2H^+)/2]$ = 438.7009), *m/z* 876.3903 (calcd. $[M+H^+]$ = 876.3939), Purity: > 99 % (HPLC analysis at 220 nm). Retention time in HPLC: 11.5 min. **HPLC method A**

No Base in H_2O with peptide 1a (entry 1)

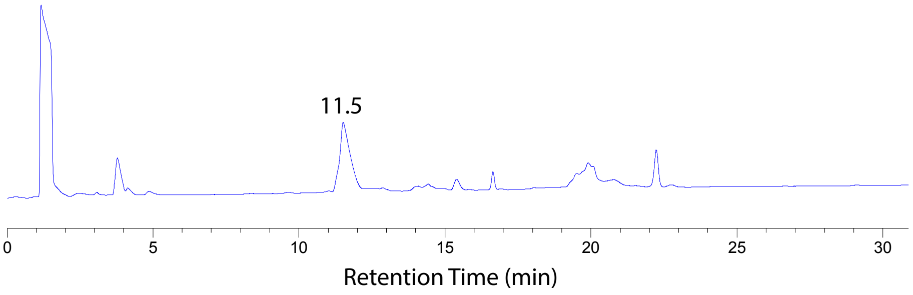

Et_3N in H_2O with peptide 1a (entry 2)

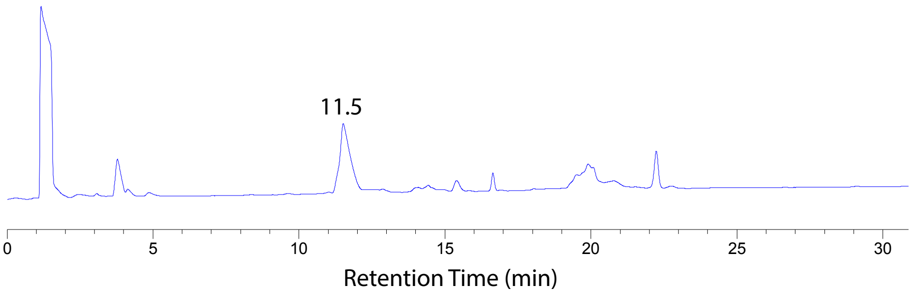

DMAP in H_2O with peptide 1a (entry 3)

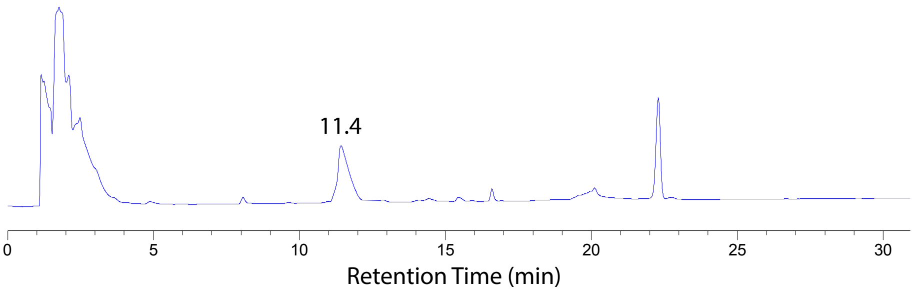

DBU in H_2O with peptide 1a (entry 4)

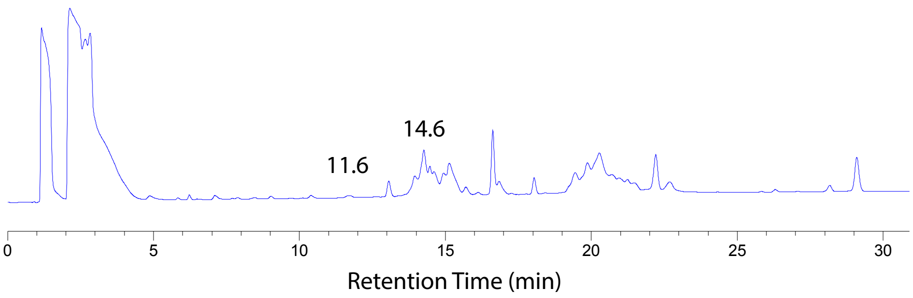

DBU in MeOH with peptide 1a (entry 5)

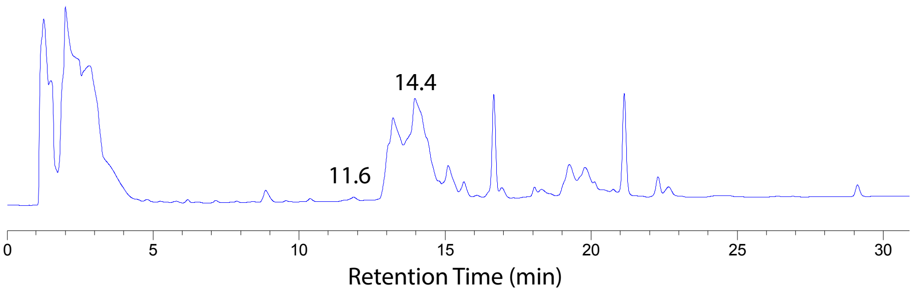

DBU in ACN with peptide 1a (entry 6)

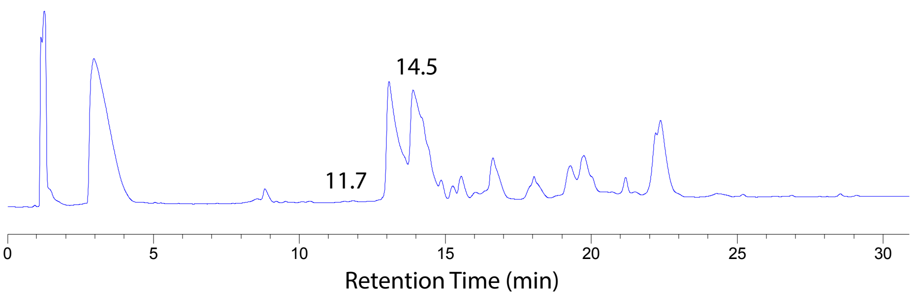

HPLC Method A probe degradation

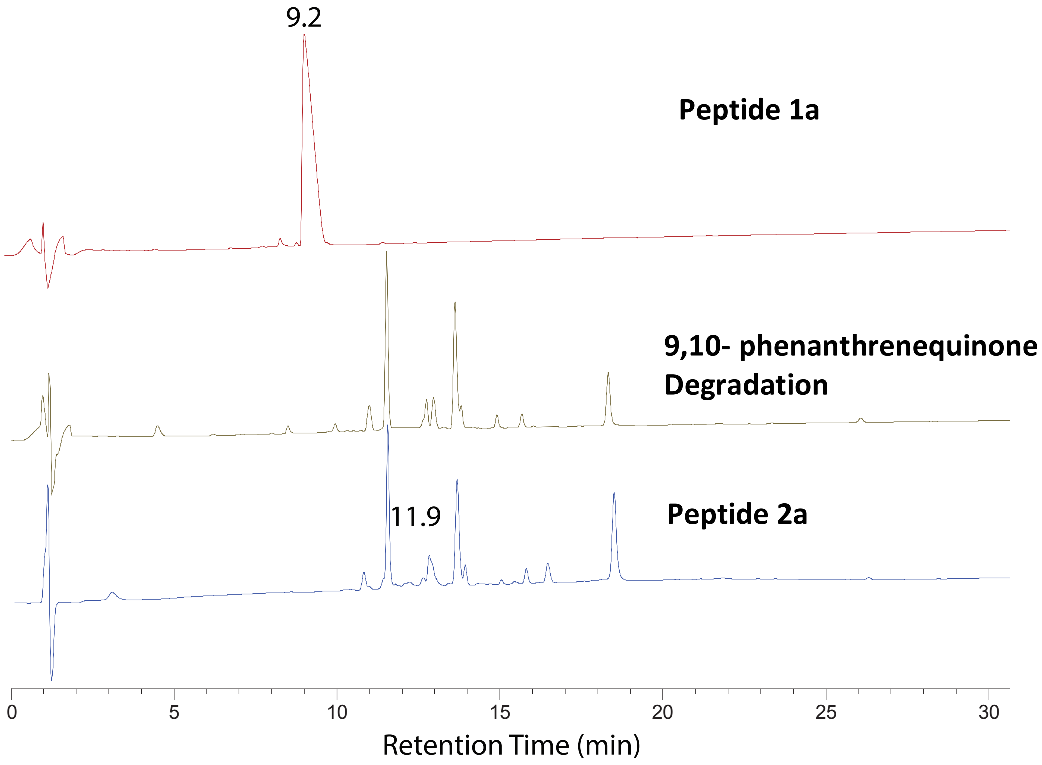

Optimized procedure for cleavage of model peptide 1a (entry 7)

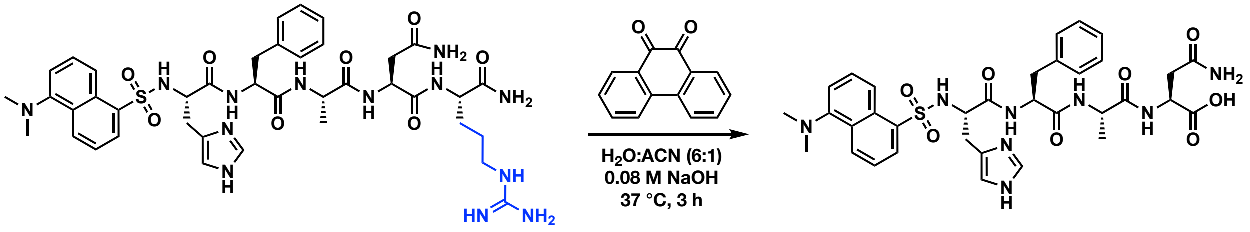

1 mg of Dansyl-HFANR-CONH$_2$ (**1a,** 1 equiv.) was dissolved in 500 µL H_2O in a 1/2” dram vial. Next, 9,10-phenanthrenequinone (0.71 mg, 3.43 µmol, 3 equiv.) from a freshly prepared stock solution in ACN was added in one portion before 258 µL H_2O was added to reach 900 µL total volume. Then 0.8 M NaOH (100 µL) was added from a freshly prepared stock solution yielding a total volume of 1 mL with a concentration of 0.08 M NaOH. The concentration of the peptide in solution was 1.14 mM. The vial was stirred at 37 °C for 3 h. The solution was filtered via syringe filtration then analyzed via **HPLC method B** to determine the percent conversion to **2a** (>99%).

Dansyl-HFANR-CONH$_2$ peptide 1a. LCMS *m/z* 438.6989 (calcd. [(M+2H$^+$)/2] = 438.7009), *m/z* 876.3903 (calcd. [M+H$^+$] = 876.3939), Purity: > 99 % (HPLC analysis at 220 nm). Retention time in HPLC: 9.2 min. **HPLC method B**

Dansyl-HFAN-CO$_2$H peptide 2a. LCMS *m/z* 361.1419 (calcd. [(M+2H$^+$)/2] = 361.1423), *m/z* 721.2724 (calcd. [M+H$^+$] = 721.2768), *m/z* 743.2581 (calcd. [M+Na$^+$] = 743.2587), (HPLC analysis at 220 nm). Retention time in HPLC: 11.9 min. *unlabeled peaks in HPLC traces are 9,10-phenanthrenequinone degradation and fluorophore by-product formation. **HPLC method B**

HPLC trace of Starting peptide 1a

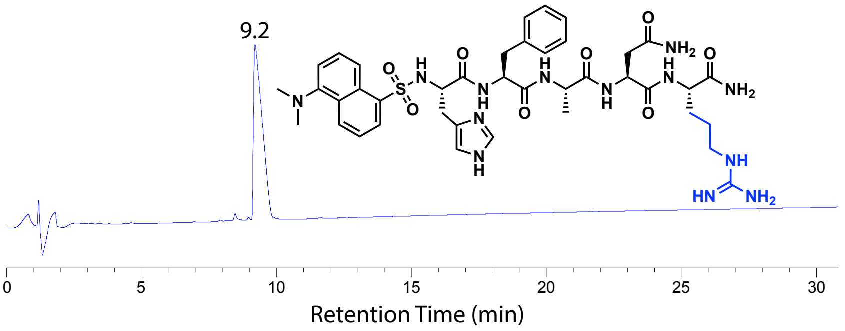

HPLC trace of Cleaved peptide 2a

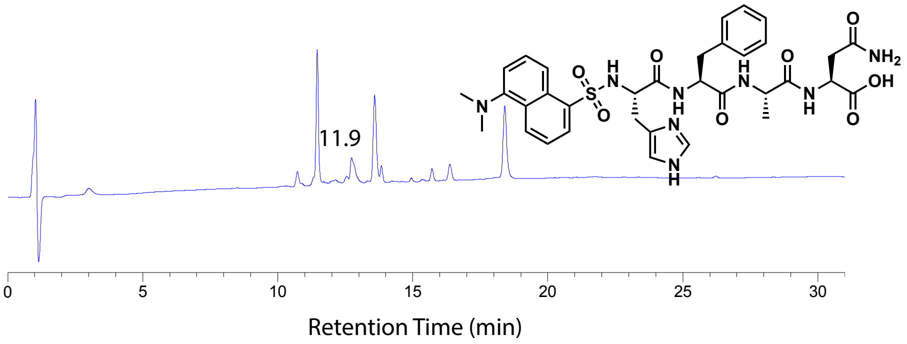

HPLC Method B-probe degradation

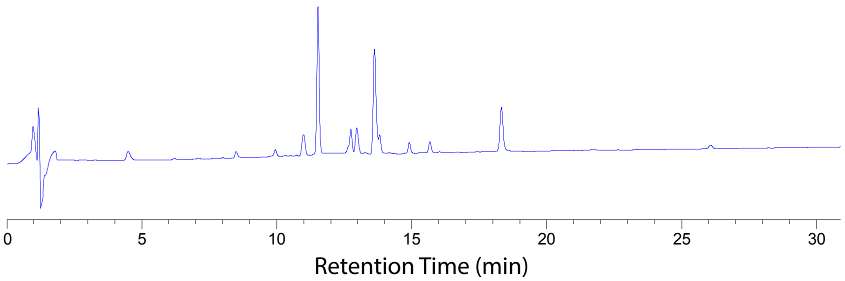

HRMS trace of 1a

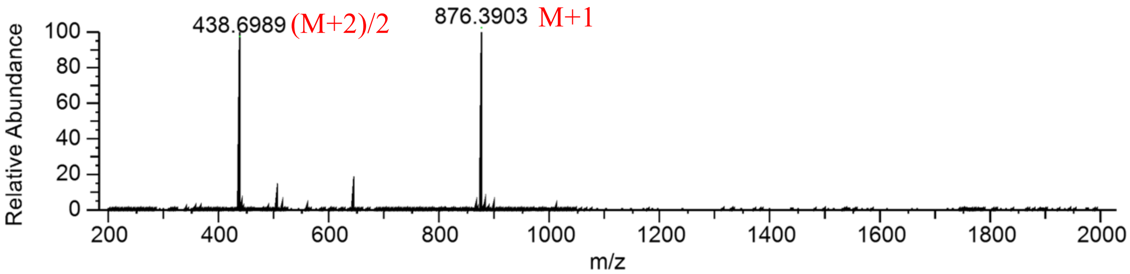

HRMS trace of 2a

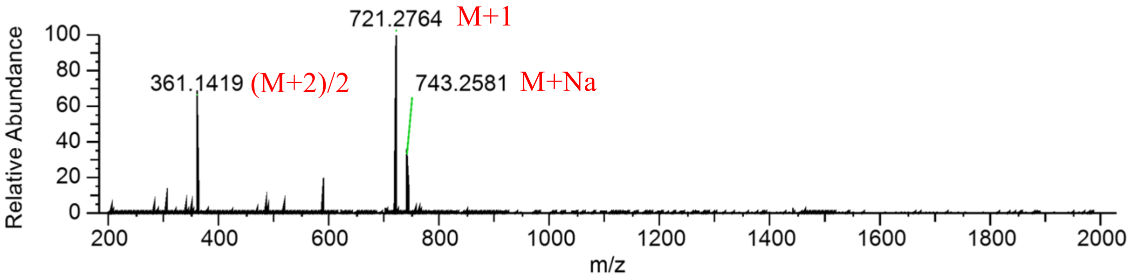

0.02M NaOH in H_2O with peptide 1a (entry 8)

Dansyl-HFANR(aldehyde)-CONH$_2$ peptide Intermediate B. LCMS *m/z* 834.3302 (calcd. [M+H$^+$] = 834.3482), (HPLC analysis at 220 nm). Retention time in HPLC: 14.0 min. **HPLC Method A**

HPLC Trace of **Intermediate B**

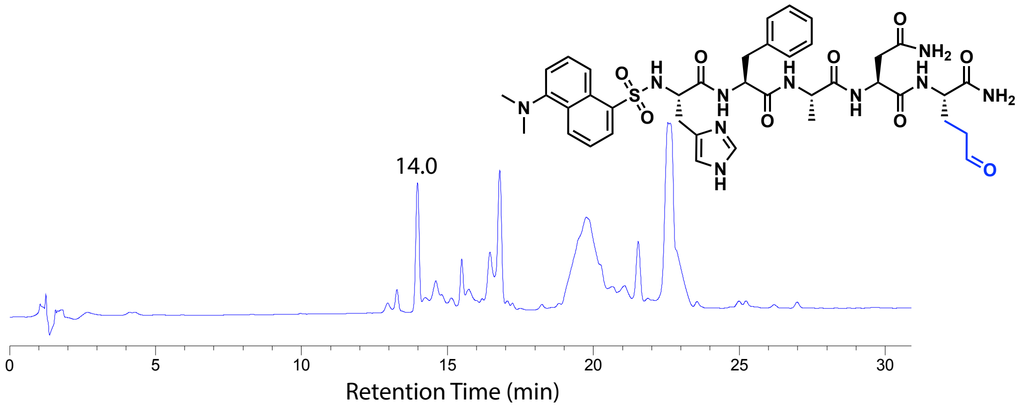

HRMS Trace of **Intermediate B**

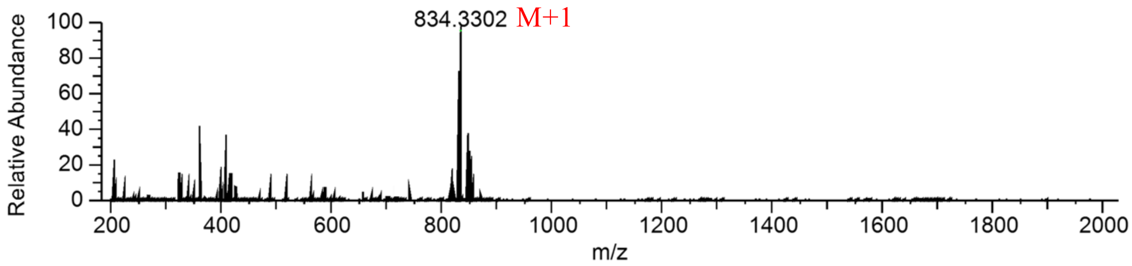

No Base in ACN with peptide 1a (entry 9)

Dansyl-HFANR(Phen)-CONH$_2$ peptide (Intermediate A). LCMS *m/z* 525.7238 (calcd. [(M+2H$^+$)/2] = 525.7243), *m/z* 1050.4410 (calcd. [M+H$^+$] = 1050.4409), (HPLC analysis at 220 nm). Retention time in HPLC: 16.7 min. **HPLC Method A**

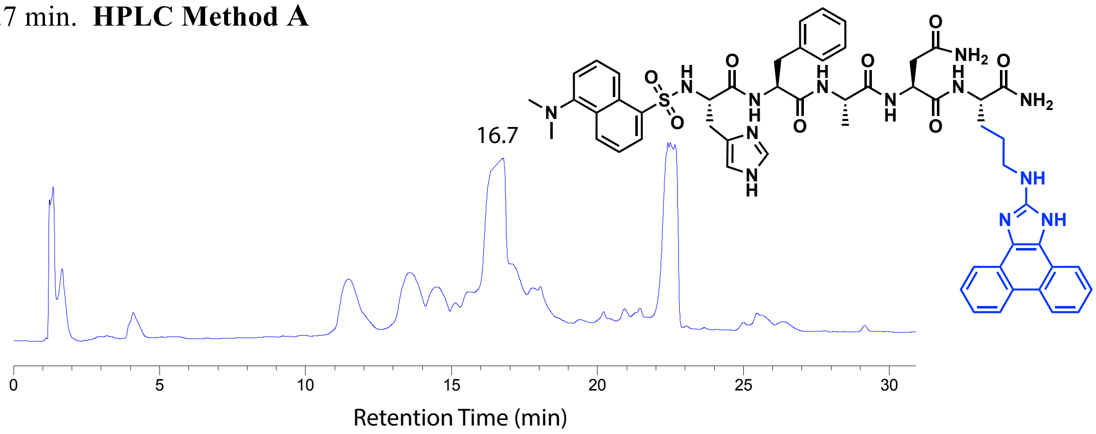

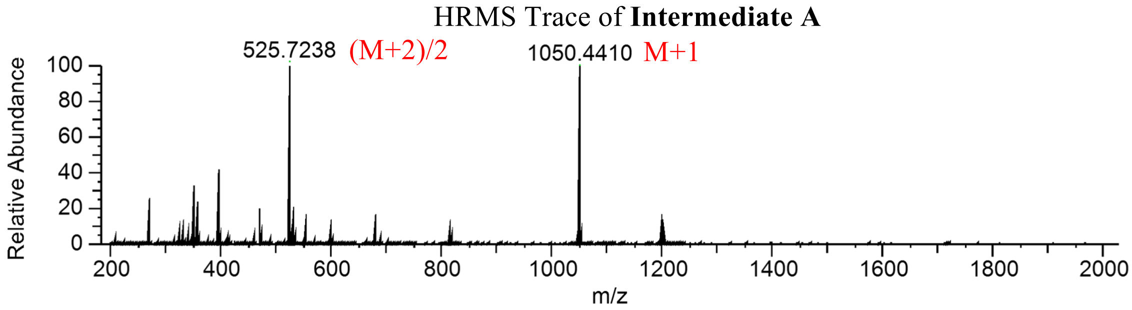

VII. Supplemental Figure 2. Characterization of cleavage product with Dansyl-HR-CONH$_2$

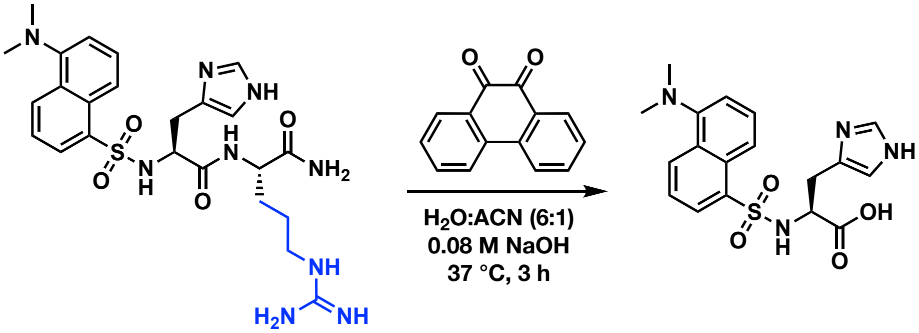

Dansyl-HR-CONH$_2$ (10 mg, 1.8 µmol, 1 eq) was dissolved in 5 mL H_2O in a 1.5" dram vial. Next, 9,10-phenanthrenequinone (1.15 mg, 5.4 µmol, 3 equiv.) from a freshly prepared stock solution in ACN was added in one portion before 2.85 mL H_2O was added to reach 9 mL total volume. Then 0.8 M NaOH (1 mL) was added from a freshly prepared stock solution yielding a total volume of 10 mL with 0.08 M NaOH. The concentration of the peptide in solution was 1.8 mM. The vial was stirred at 37 °C for 3 h. The solution was filtered via syringe filtration then analyzed via HPLC method B to determine the percent conversion to cleaved product Dansyl-H-CO$_2$H (>99%). Fractions were combined and lyophilized revealing a fluffy white solid for characterization.

Dansyl-HR-CONH$_2$ peptide.
LCMS, *m/z* 272.6268 (calcd. [(M+2H$^+$)/2] = 272.6266), *m/z* 544.260 (calcd. [M+H$^+$] = 544.2454) Purity: > 99 % (HPLC analysis at 220 nm). Retention time in HPLC: 5.9 min.

Dansyl-H-CO$_2$H peptide.
LCMS *m/z* 389.1490 (calcd. [M+H$^+$] = 389.1285), *m/z* 411.1309 (calcd. [M+Na$^+$] = 411.1102), *m/z* 799.2727 (calcd. [2M+Na$^+$] = 799.2308), (HPLC analysis at 220 nm). Retention time in HPLC: 8.8 min.

^{1}H NMR (400 MHz, CD$_3$OD, ppm) δ: 8.52 (1H, d, J = 8.5 Hz), 8.44 (1H, s), 8.18 (1H, d, J = 8.6 Hz), 8.11 (1H, d, J = 7.4 Hz), 7.52 (2H, t, J = 8.0 Hz), 7.25 (2H, m), 6.58 (1H, s), 3.96 (1H, q, J = 4.7 Hz), 2.89 (6H, s), 2.87 (1H, t, J = 5.5 Hz), 2.71 (1H, app. q, J = 9.6 Hz).
^{13}C NMR (101 MHz, CD$_3$OD, ppm) δ: 176.0, 152.9, 136.5, 135.4, 133.4, 131.3, 131.1, 130.6, 130.4, 128.9, 124.2, 120.7, 118.0, 116.4, 58.1, 45.8, 31.0.

HPLC trace of Dansyl-HR-CONH$_2$

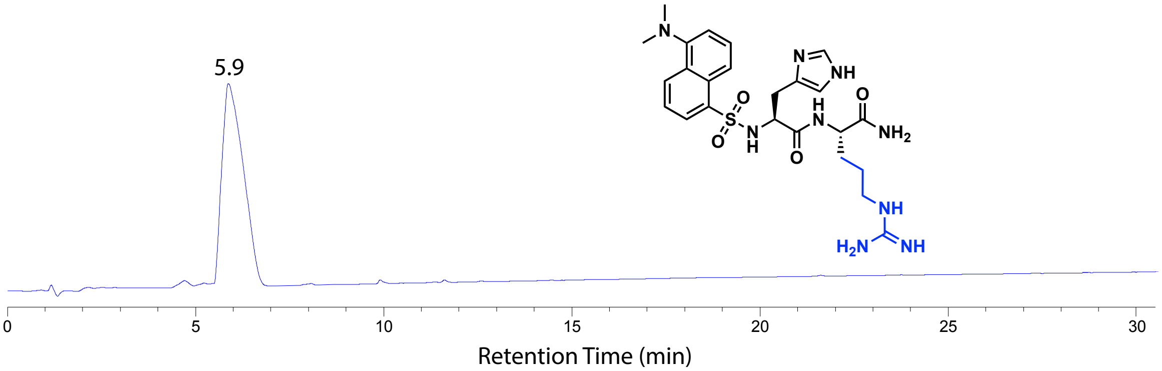

HPLC trace of Dansyl-H-CO$_2$H

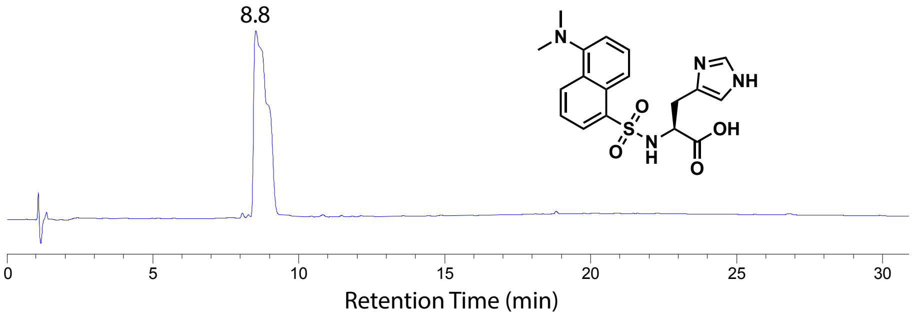

HRMS trace of 1 Dansyl-HR-CONH$_2$

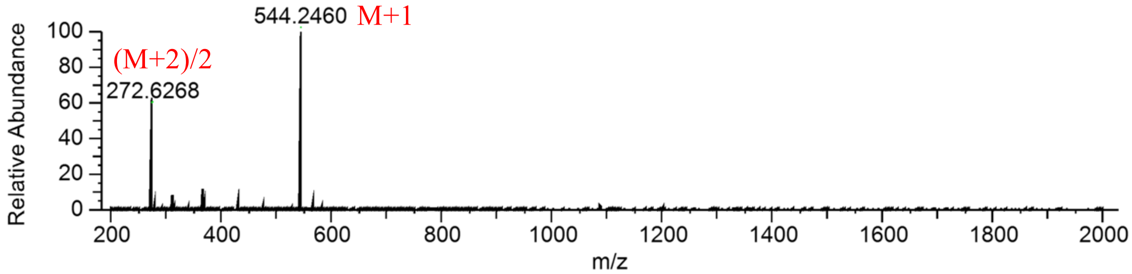

HRMS trace of Dansyl-H-CO$_2$H

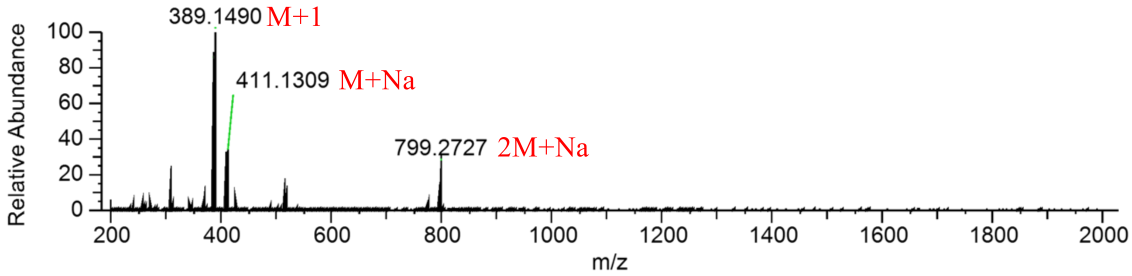

^{1}H NMR Dansyl-H-CONH$_2$

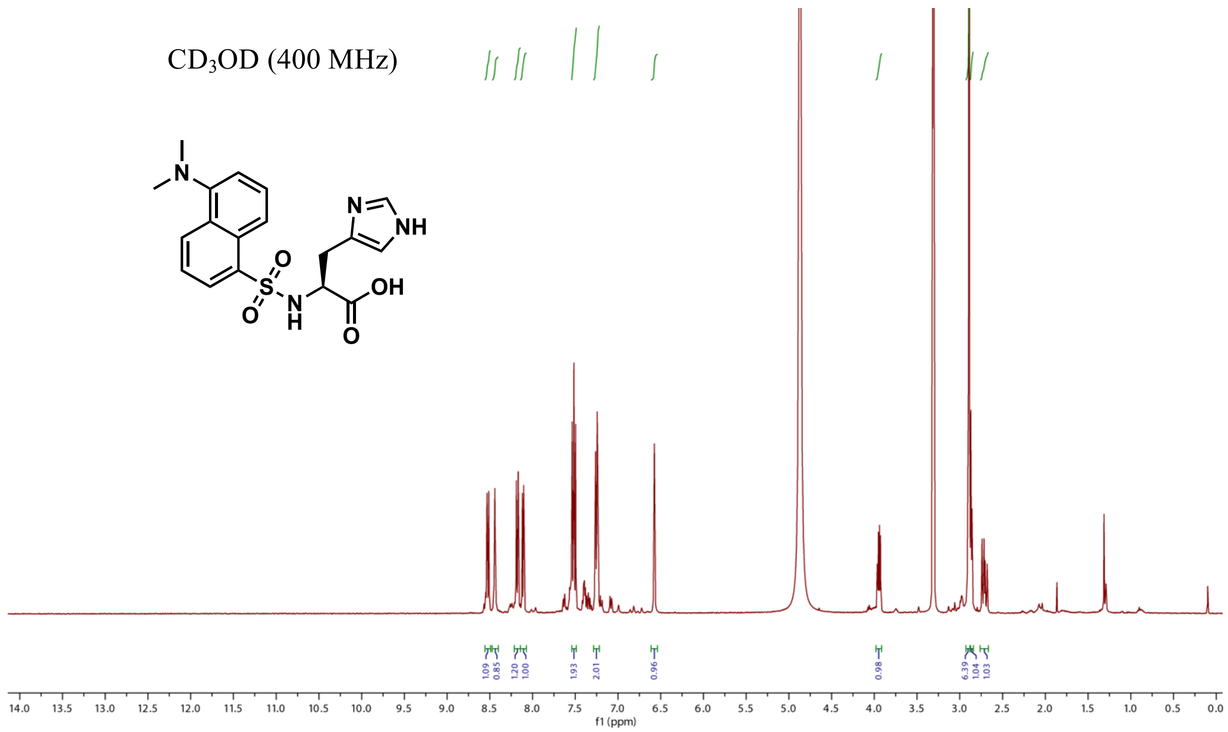

^{13}C NMR Dansyl-H-CO$_2$H

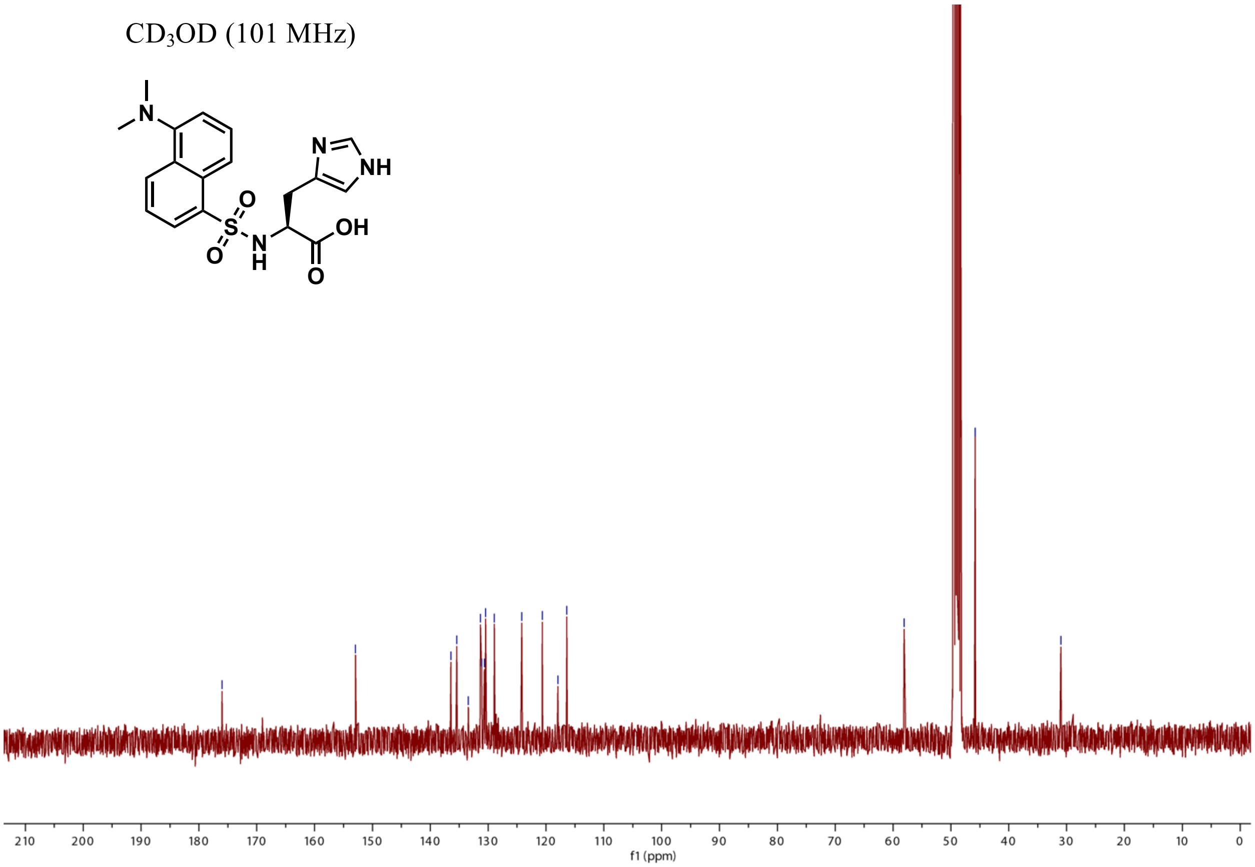

VIII. Supplemental Figure 3. Characterization of Fluorophore by-product

Fluorophore by-product. White solid. ^{1}H NMR (400 MHz, DMSO-d$_6$, ppm) δ: 8.79 (2H, d, J = 8.2 Hz), 8.30 (1H, s), 8.22 (2H, d, J = 8.1 Hz), 7.61 (2H, app. t, J = 7.4 Hz), 7.49 (2H, app. t, J = 7.5 Hz), 6.22 (1H, s).
^{13}C NMR (101 MHz, CD_3OD, ppm) δ: 164.58, 154.04, 126.39, 125.97, 123.61, 123.21, 120.99.
LCMS m/z 234.1026 (calcd. [M+H$^+$] = 234.1031).

^{1}H NMR Fluorophore by-product

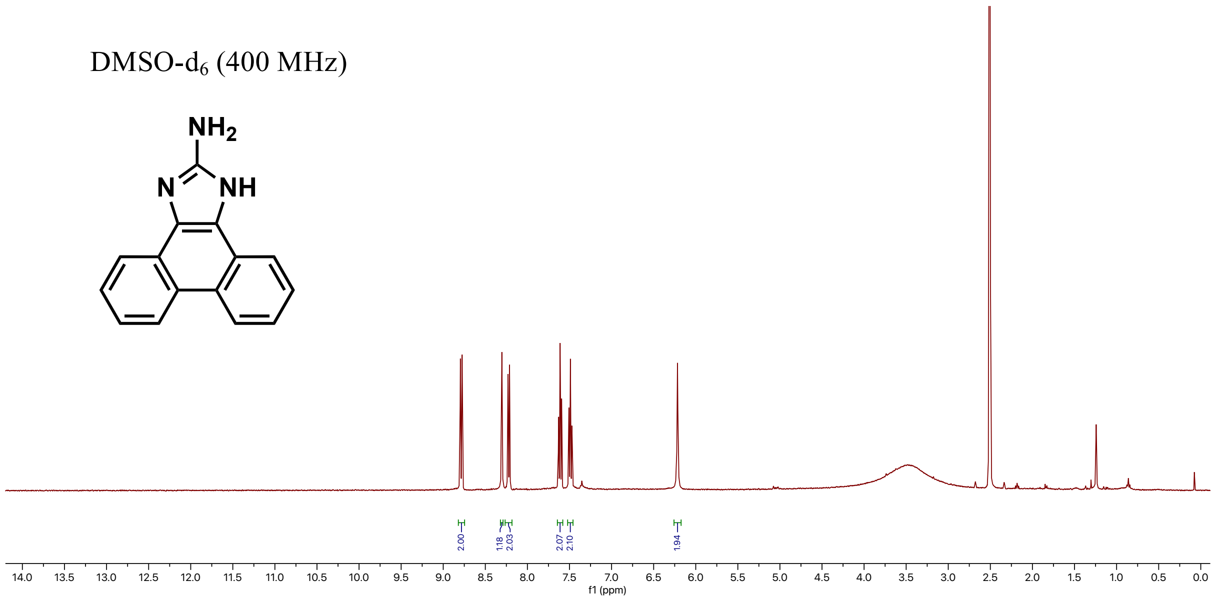

^{13}C NMR Fluorophore by-product

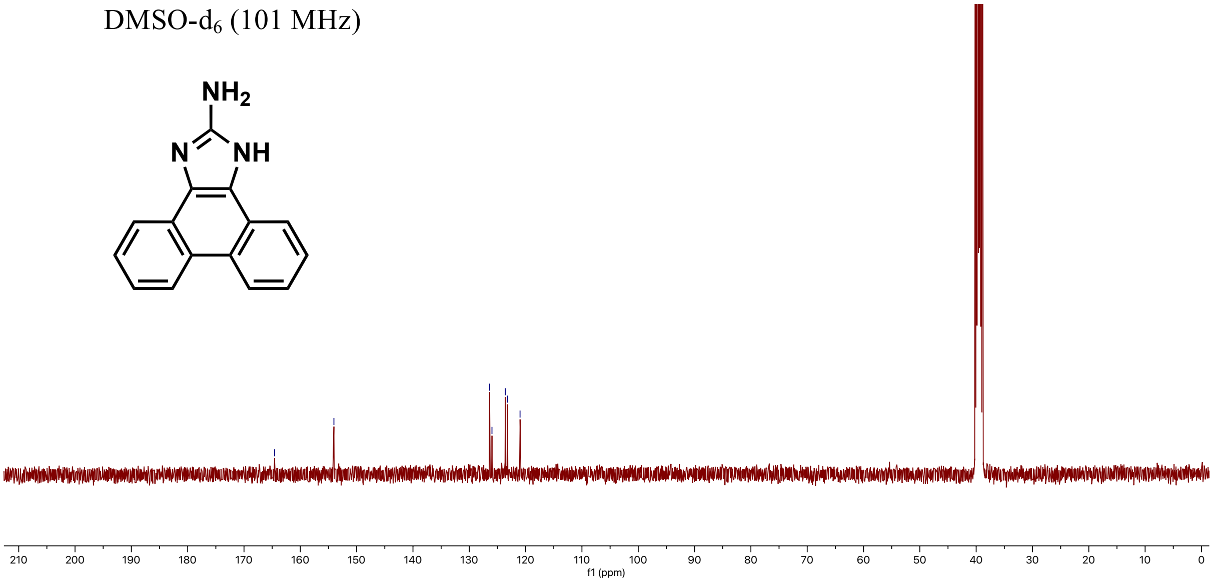

HRMS of Fluorophore by-product

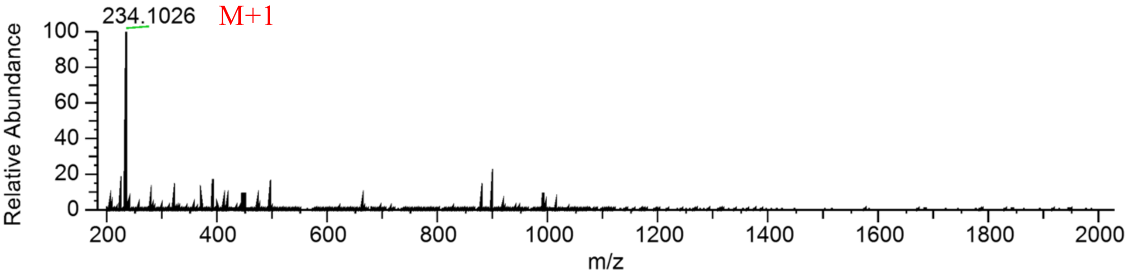

IX. Supplementary Figure 4. Chemoselectivity Studies of Dansyl-XHG-CO_2H Peptides

Chemoselectivity with Cysteine peptide, Dansyl-CHG-CO_2H

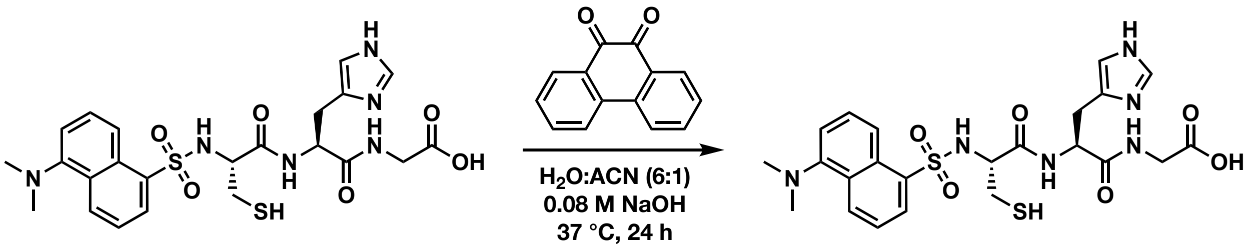

Dansyl-CHG-CO_2H (1 mg, 1.82 µmol, 1 equiv.) was dissolved in 500 µL H_2O in a 1/2” dram vial. Next, 9,10-phenanthrenequinone (1.14 mg, 5.47 µmol, 3 equiv.) from a freshly prepared stock solution in ACN was added in one portion before 172 µL H_2O was added to reach 900 µL total volume. Then 0.8 M NaOH (100 µL) was added from a freshly prepared stock solution yielding a total volume of 1 mL with 0.08 M NaOH. The concentration of the peptide in solution was 1.91 mM. The vial was stirred at 37 °C for 24 h. The solution was filtered via syringe filtration then analyzed via HPLC method B. No modification occurred.

Dansyl-CHG-CO_2H peptide. LCMS, *m/z* 275.0768 (calcd. $[(M+2H^+)/2]$ = 275.0834), *m/z* 549.1458 (calcd. $[M+H^+]$ = 549.1590) Purity: > 99 % (HPLC analysis at 220 nm). Retention time in HPLC: 9.8 min.

HPLC trace of Starting peptide Dansyl-CHG-CO_2H

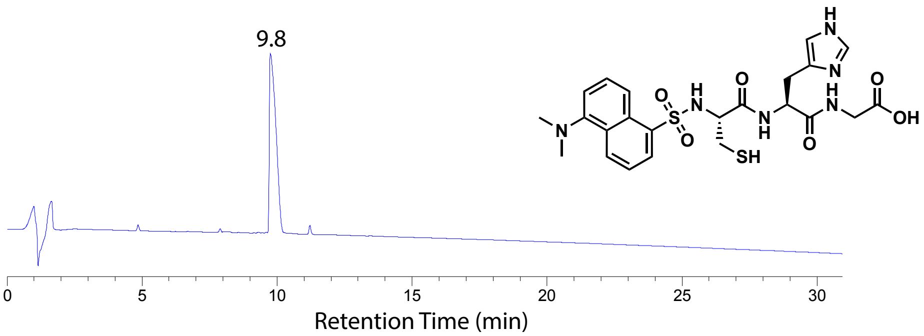

HPLC trace of Reaction Dansyl-CHG-CO_2H

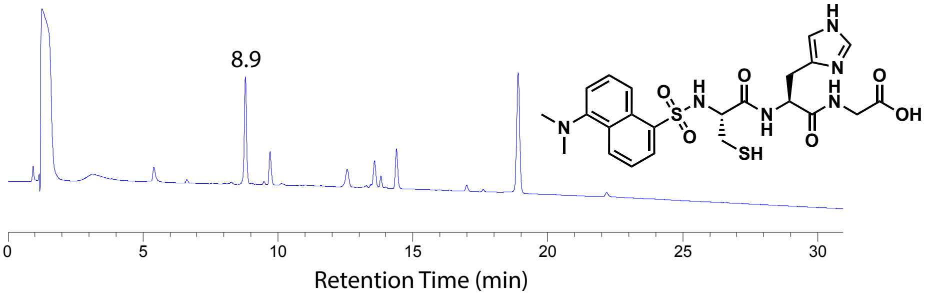

HRMS trace of Dansyl-CHG-CO_2H

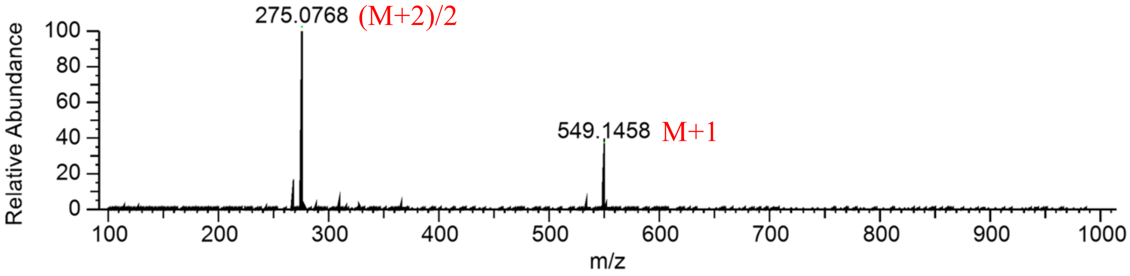

Chemoselectivity with Aspartate peptide, Dansyl-DHG-CO_2H

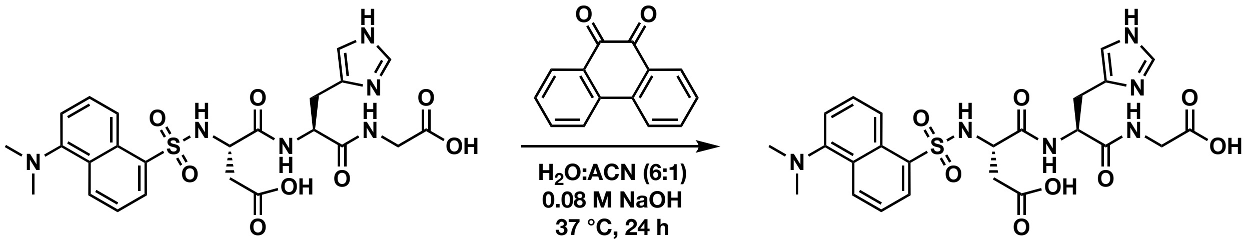

Dansyl-DHG-CO_2H (1 mg, 1.79 µmol, 1 equiv.) was dissolved in 500 µL H_2O in a 1/2" dram vial. Next, 9,10-phenanthrenequinone (1.12 mg, 5.36 µmol, 3 equiv.) from a freshly prepared stock solution in ACN was added in one portion before 176 µL H_2O was added to reach 900 µL total volume. Then 0.8 M NaOH (100 µL) was added from a freshly prepared stock solution yielding a total volume of 1 mL with 0.08 M NaOH. The concentration of the peptide in solution was 1.79 mM. The vial was stirred at 37 °C for 24 h. The solution was filtered via syringe filtration then analyzed via HPLC method B. No modification occurred.

Dansyl-DHG-CO_2H peptide. LCMS, *m/z* 561.1684 (calcd. [M+H^+] = 561.1767) Purity: > 99 % (HPLC analysis at 220 nm). Retention time in HPLC: 10.0 min.

HPLC trace of Starting peptide Dansyl-DHG-CO_2H

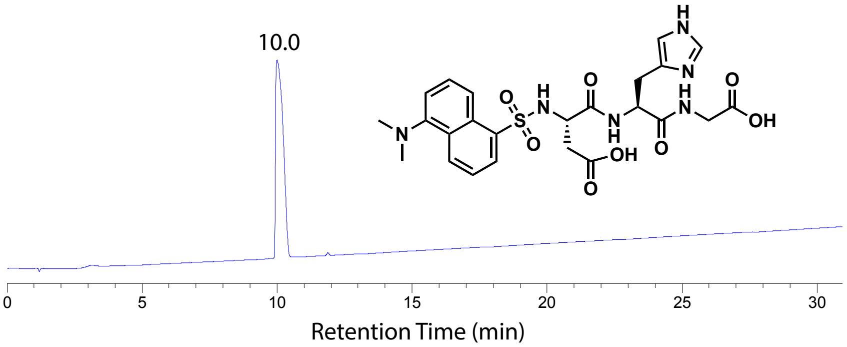

HPLC trace of Reaction Dansyl-DHG-CO_2H

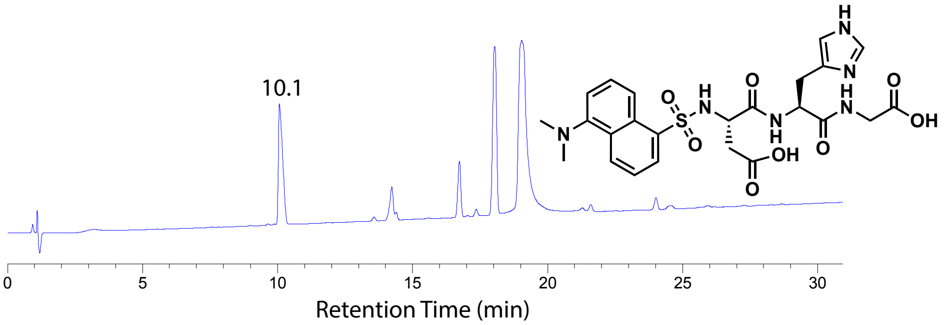

HRMS trace of Dansyl-DHG-CO_2H

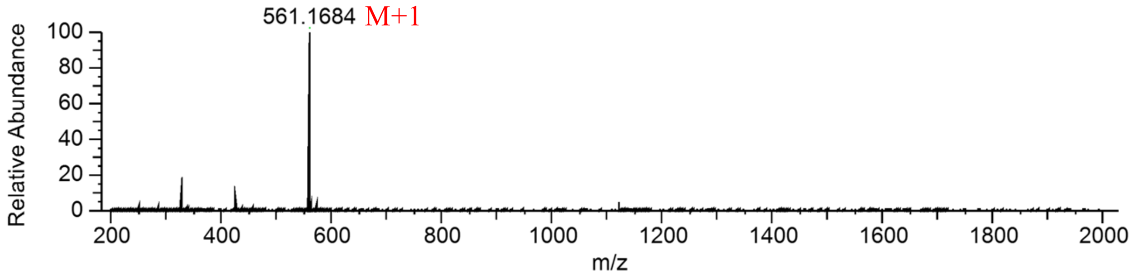

Chemoselectivity with Lysine peptide, Dansyl-KHG-CO_2H

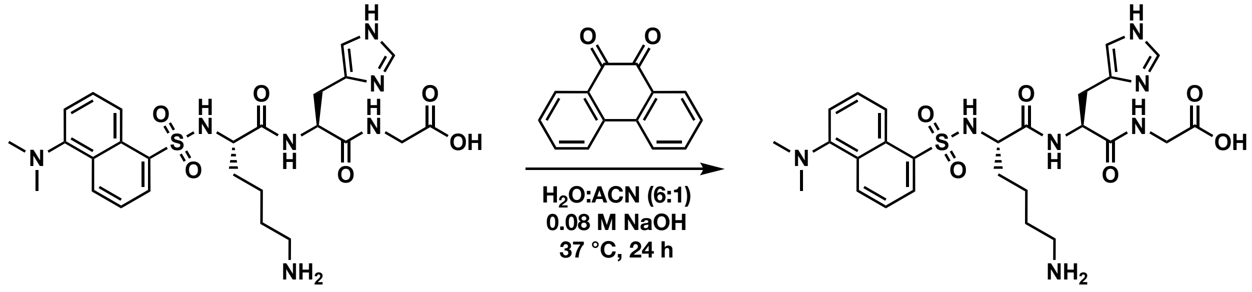

Dansyl-KHG-CO_2H (1 mg, 1.74 µmol, 1 equiv.) was dissolved in 500 µL H_2O in a 1/2" dram vial. Next, 9,10-phenanthrenequinone (1.09 mg, 5.23 µmol, 3 equiv.) from a freshly prepared stock solution in ACN was added in one portion before 182 µL H_2O was added to reach 900 µL total volume. Then 0.8 M NaOH (100 µL) was added from a freshly prepared stock solution yielding a total volume of 1 mL with 0.08 M NaOH. The concentration of the peptide in solution was 1.74 mM. The vial was stirred at 37 °C for 24 h. The solution was filtered via syringe filtration then analyzed via HPLC method B. No modification occurred.

Dansyl-KHG-CO_2H peptide. LCMS, *m/z* 287.6285 (calcd. $[(M{+}2H^+)/2]$ = 287.6263), *m/z* 574.2368 (calcd. $[M{+}H^+]$ = 574.2447) Purity: > 99 % (HPLC analysis at 220 nm). Retention time in HPLC: 7.8 min.

HPLC trace of Starting peptide Dansyl-KHG-CO_2H

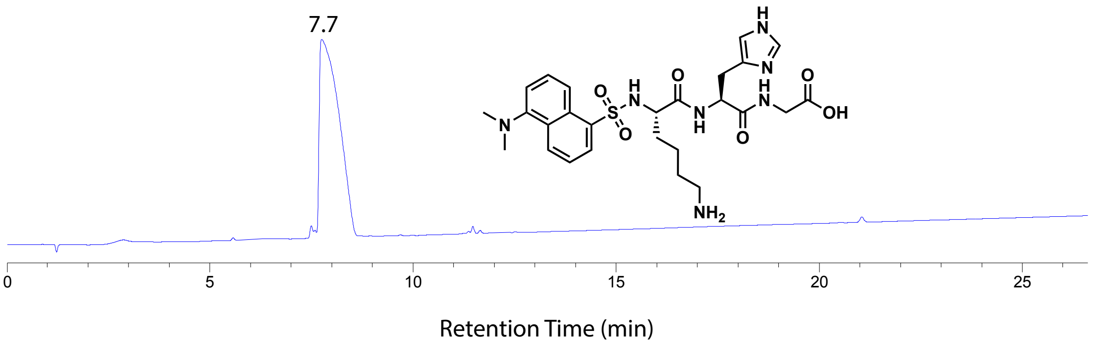

HPLC trace of Reaction Dansyl-KHG-CO_2H

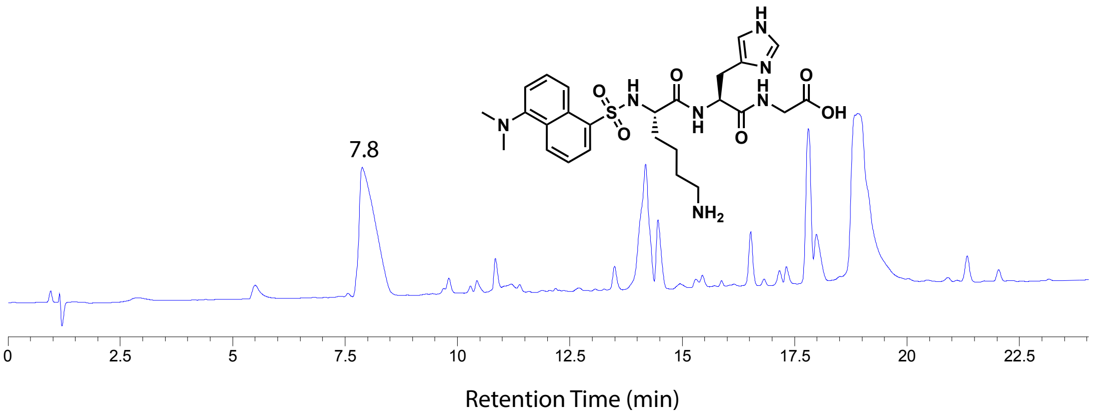

HRMS trace of Dansyl-KHG-CO_2H

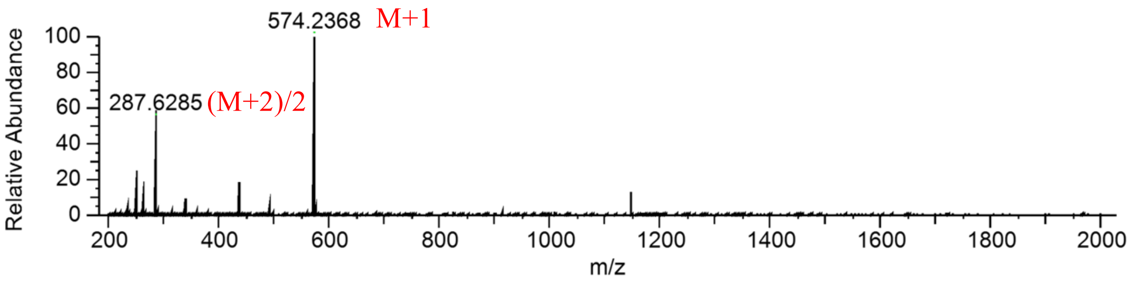

Chemoselectivity with Methionine peptide, Dansyl-MHG-CO_2H

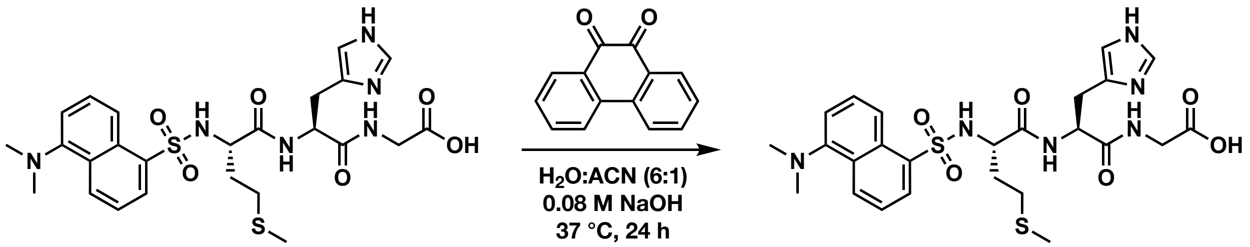

Dansyl-MHG-CO_2H (1 mg, 1.74 µmol, 1 equiv.) was dissolved in 500 µL H_2O in a 1/2" dram vial. Next, 9,10-phenanthrenequinone (1.08 mg, 5.21 µmol, 3 equiv.) from a freshly prepared stock solution in ACN was added in one portion before 184 µL H_2O was added to reach 900 µL total volume. Then 0.8 M NaOH (100 µL) was added from a freshly prepared stock solution yielding a total volume of 1 mL with 0.08 M NaOH. The concentration of the peptide in solution was 1.74 mM. The vial was stirred at 37 °C for 24 h. The solution was filtered via syringe filtration then analyzed via HPLC method A. No modification occurred.

Dansyl-MHG-CO_2H peptide. LCMS, *m/z* 289.0921 (calcd. $[(M{+}2H^+)/2]$ = 289.0991), *m/z* 577.1768 (calcd. $[M{+}H^+]$ = 577.1903) Purity: > 99 % (HPLC analysis at 220 nm). Retention time in HPLC: 14.7 min.

HPLC trace of Starting peptide Dansyl-MHG-CO_2H

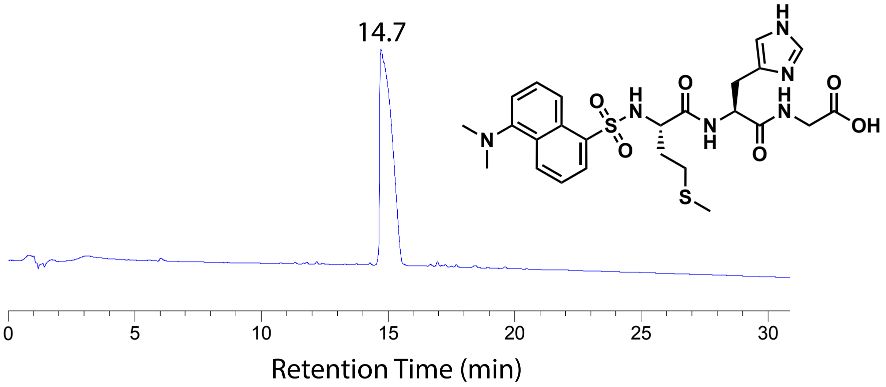

HPLC trace of Reaction Dansyl-MHG-CO$_2$H

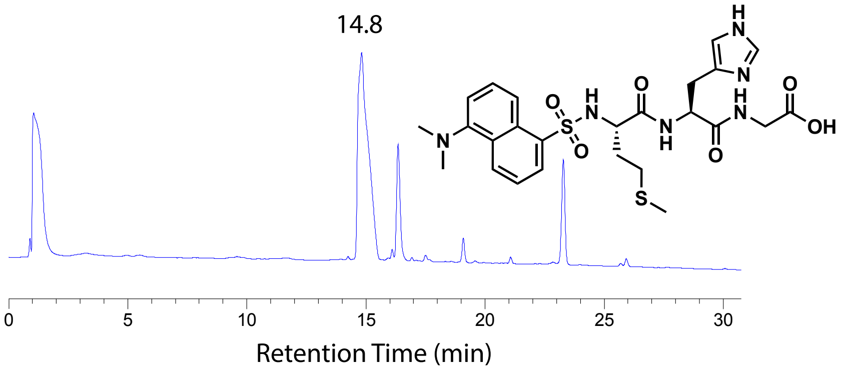

HRMS trace of Dansyl-MHG-CO$_2$H

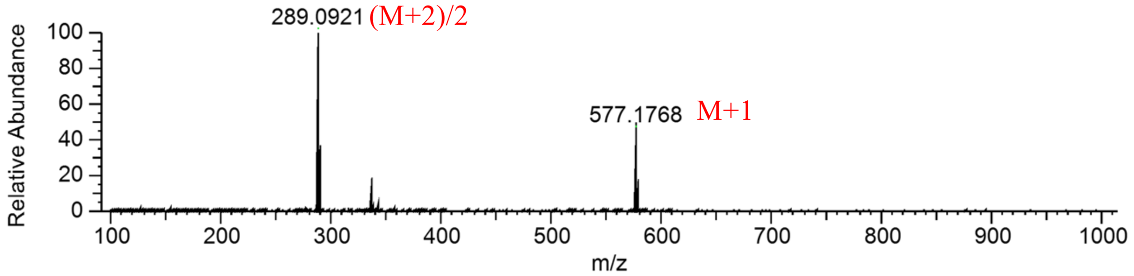

Chemoselectivity with Serine peptide, Dansyl-SHG-CO$_2$H

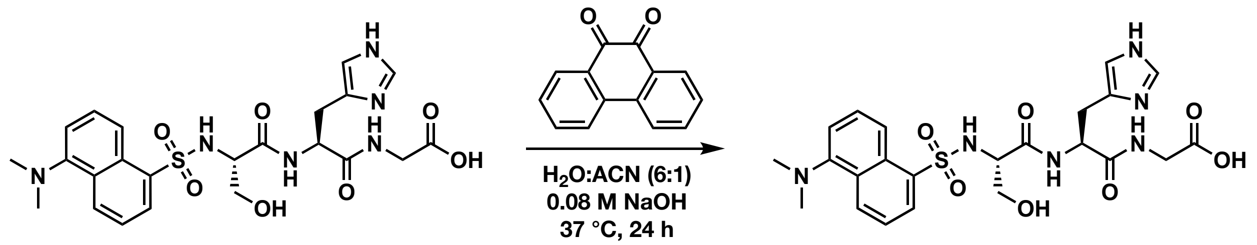

Dansyl-SHG-CO$_2$H (1 mg, 1.88 µmol, 1 equiv.) was dissolved in 500 µL H_2O in a 1/2” dram vial. Next, 9,10-phenanthrenequinone (1.17 mg, 5.64 µmol, 3 equiv.) from a freshly prepared stock solution in ACN was added in one portion before 166 µL H_2O was added to reach 900 µL total volume. Then 0.8 M NaOH (100 µL) was added from a freshly prepared stock solution yielding a total volume of 1 mL with 0.08 M NaOH. The concentration of the peptide in solution was 1.88 mM. The vial was stirred at 37 °C for 24 h. The solution was filtered via syringe filtration then analyzed via HPLC method B. No modification occurred.

Dansyl-SHG-CO$_2$H peptide. LCMS, *m/z* 267.0082 (calcd. $[(M+2H^+)/2]$ = 267.0648), *m/z* 533.1689 (calcd. $[M+H^+]$ = 533.1818) Purity: > 99 % (HPLC analysis at 220 nm). Retention time in HPLC: 7.7 min.

HPLC trace of Starting peptide Dansyl-SHG-CO_2H

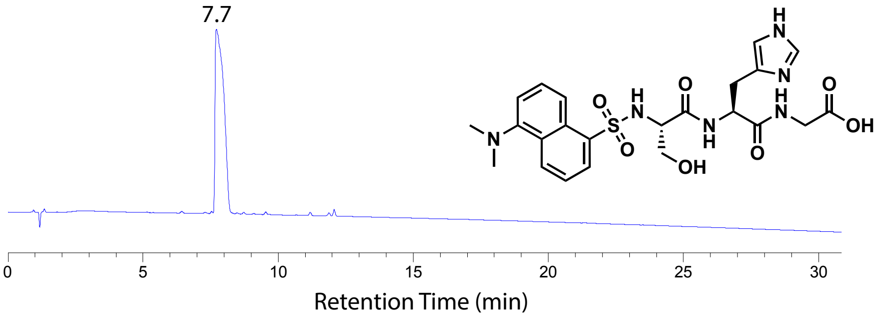

HPLC trace of Reaction Dansyl-SHG-CO_2H

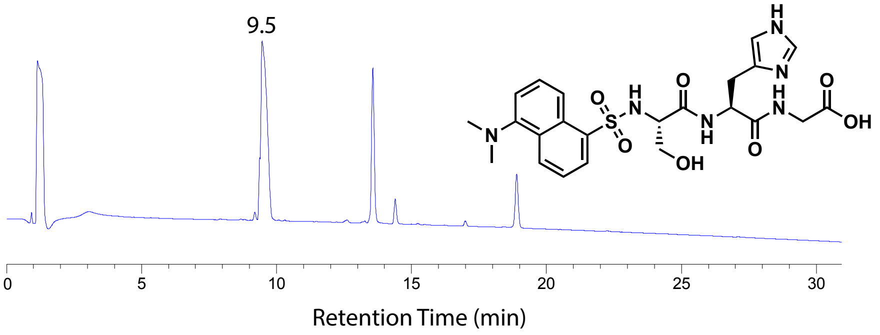

HRMS trace of Dansyl-SHG-CO_2H

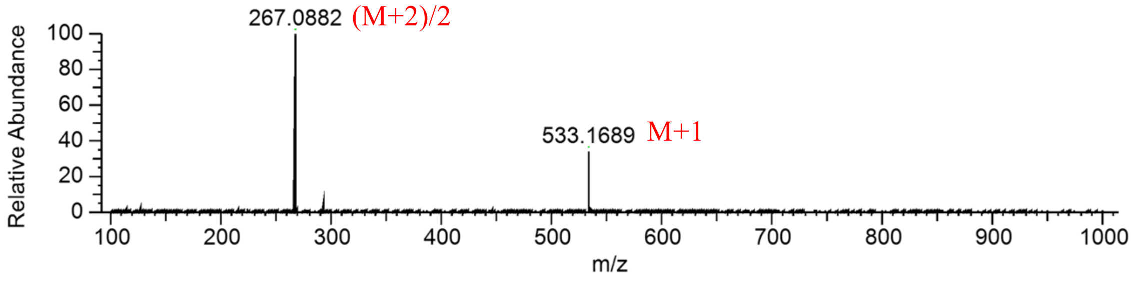

Chemoselectivity with Tryptophan peptide, Dansyl-WHG-CO_2H

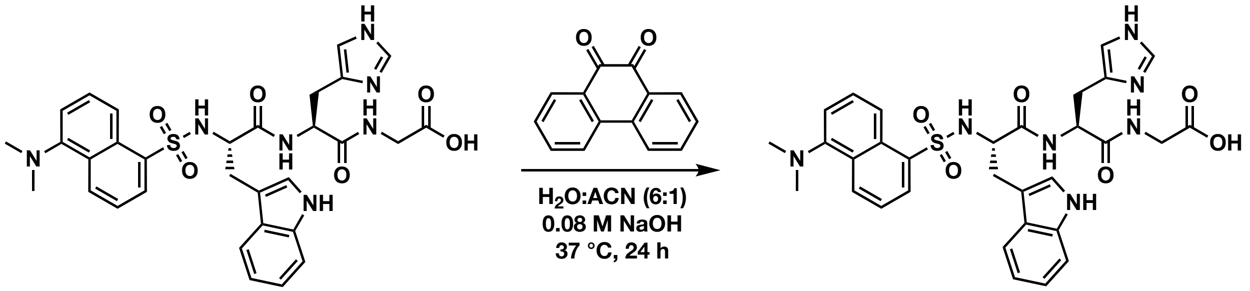

Dansyl-WHG-CO_2H (1 mg, 1.58 µmol, 1 equiv.) was dissolved in 500 µL H_2O in a 1/2" dram vial. Next, 9,10-phenanthrenequinone (1.0 mg, 4.75 µmol, 3 equiv.) from a freshly prepared stock solution in ACN was added in one portion before 200 µL H_2O was added to reach 900 µL total volume. Then 0.8 M NaOH (100 µL) was added from a freshly prepared stock solution yielding a total volume of 1 mL with 0.08 M NaOH. The concentration of the peptide in solution was 1.58 mM. The vial was stirred at 37 °C for 24 h. The solution was filtered via syringe filtration then analyzed via HPLC method A. No modification occurred.

Dansyl-WHG-CO_2H peptide. LCMS, *m/z* 316.6174 (calcd. $[(M+2H^+)/2]$ = 316.6184), *m/z* 63.2275 (calcd. $[M+H^+]$ = 632.2291) Purity: > 99 % (HPLC analysis at 220 nm). Retention time in HPLC: 16.1 min.

HPLC trace of Starting peptide Dansyl-WHG-CO_2H

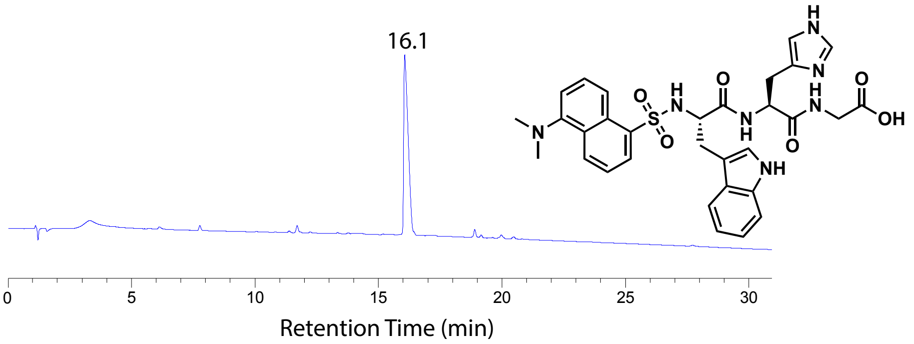

HPLC trace of Reaction Dansyl-WHG-CO_2H

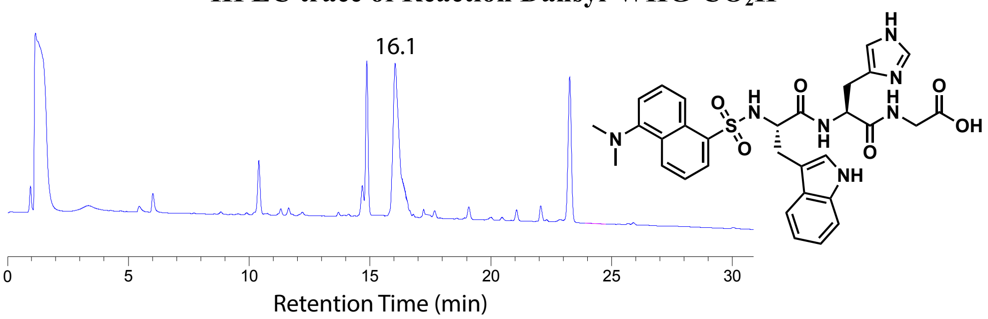

HRMS trace of Dansyl-WHG-CO₂H

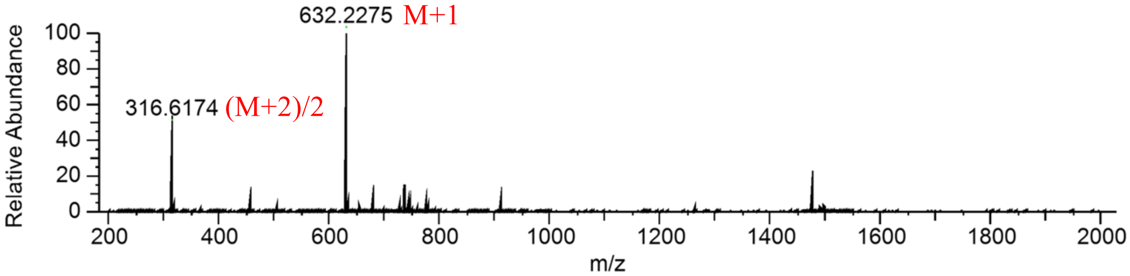

Chemoselectivity with Tyrosine peptide, Dansyl-YHG-CO₂H

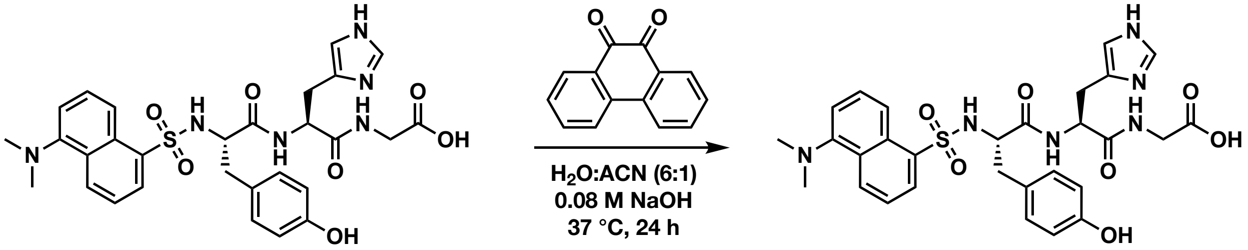

Dansyl-YHG-CO_2H (1 mg, 1.64 µmol, 1 equiv.) was dissolved in 500 µL H_2O in a 1/2” dram vial. Next, 9,10-phenanthrenequinone (1.03 mg, 4.93 µmol, 3 equiv.) from a freshly prepared stock solution in ACN was added in one portion before 194 µL H_2O was added to reach 900 µL total volume. Then 0.8 M NaOH (100 µL) was added from a freshly prepared stock solution yielding a total volume of 1 mL with 0.08 M NaOH. The concentration of the peptide in solution was 1.64 mM. The vial was stirred at 37 °C for 24 h. The solution was filtered via syringe filtration then analyzed via HPLC method B. No modification occurred.

Dansyl-YHG-CO_2H peptide. LCMS, *m/z* 305.1093 (calcd. $[(M+2H^+)/2]$ = 305.1105), *m/z* 609.2109 (calcd. $[M+H^+]$ = 609.2131) Purity: > 99 % (HPLC analysis at 220 nm). Retention time in HPLC: 12.4 min.

HPLC trace of Starting peptide Dansyl-YHG-CO₂H

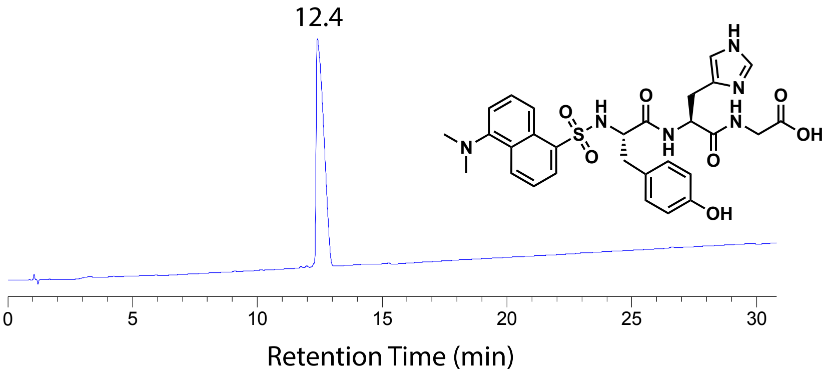

HPLC trace of Reaction Dansyl-YHG-CO$_2$H

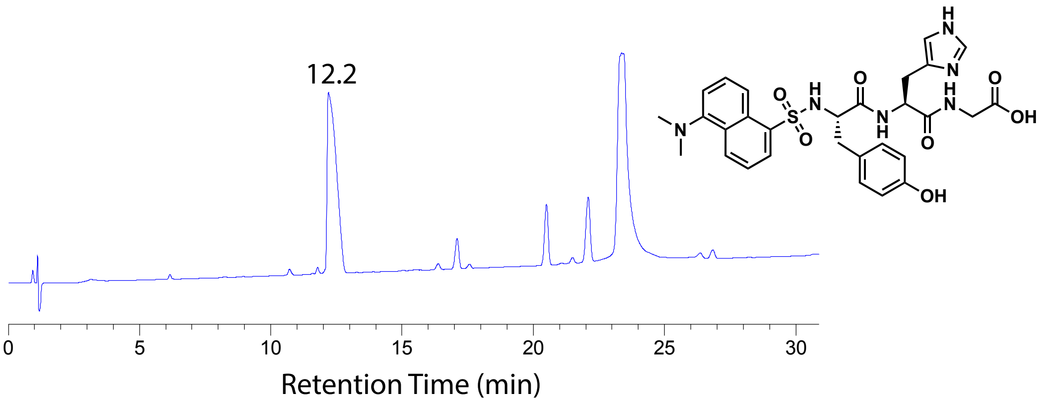

HRMS trace of Dansyl-YHG-CO$_2$H

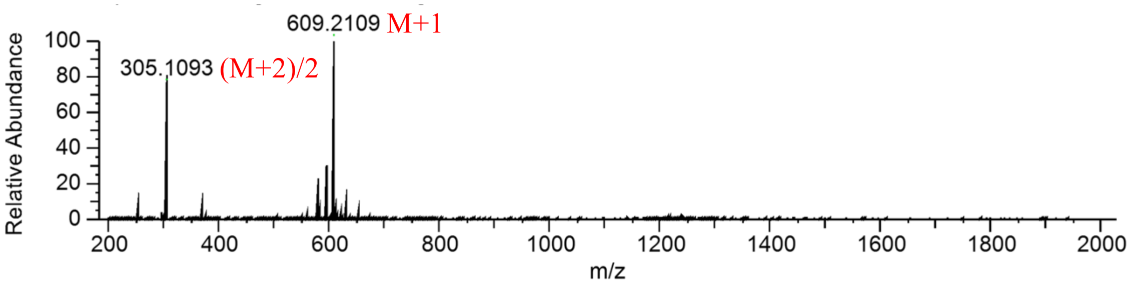

X. Supplementary Figure 5. Non-C-terminal Arginine peptides
Imine formation of Free N-terminal Arginine peptide.

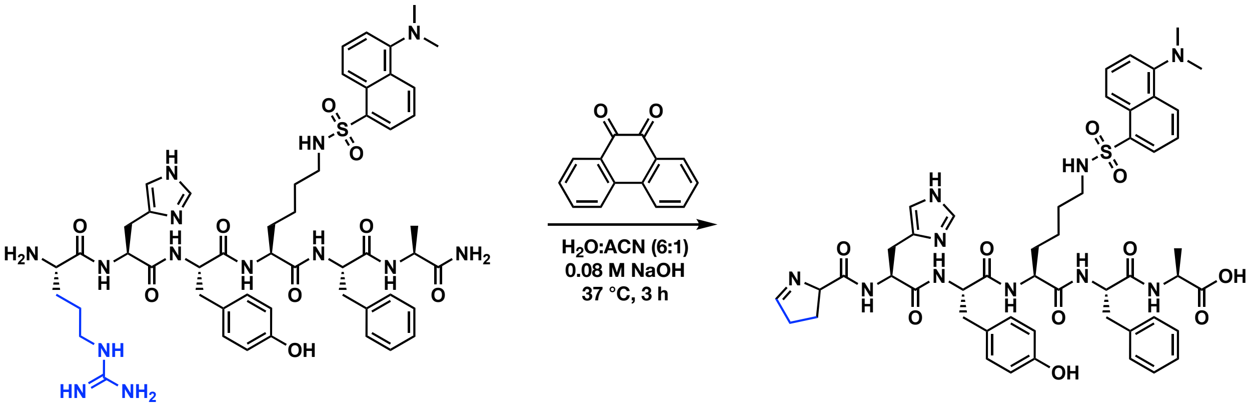

H_2N-RHYK(dansyl)FA-$CONH_2$ (1 mg, 0.95 µmol, 1 equiv.) was dissolved in 500 µL H_2O in a 1/2" dram vial. Next, 9,10-phenanthrenequinone (0.59 mg, 2.85 µmol, 3 equiv.) from a freshly prepared stock solution in ACN was added in one portion before 282 µL H_2O was added to reach 900 µL total volume. Then 0.8 M NaOH (100 µL) was added from a freshly prepared stock solution yielding a total volume of 1 mL with a concentration of 0.08 M NaOH. The concentration of the peptide in solution was 0.95 mM.

The vial was stirred at 37 °C for 3 h. The solution was filtered via syringe filtration then analyzed via HPLC method A to determine the percent conversion to imine product.

H_2N-RHYK(Dansyl)FA-$CONH_2$ peptide. LCMS *m/z* 527.2570 (calcd. [(M+2H^+)/2] = 527.2585), *m/z* 1053.5066 (calcd. [M+H^+] = 1053.5092), Purity: > 99 % (HPLC analysis at 220 nm). Retention time in HPLC: 9.2 min.

Imine-HYK(Dansyl)FA-$CONH_2$ peptide. LCMS *m/z* 331.8151 (calcd. [(M+3H^+)/3] = 331.8150), *m/z* 497.2188 (calcd. [(M+2H^+)/2] = 497.2186), *m/z* 993.4301 (calcd. [M+H^+] = 993.4293), (HPLC analysis at 220 nm). Retention time in HPLC: 15.3 min.

HPLC trace of H_2N-RHYK(Dansyl)FA-$CONH_2$

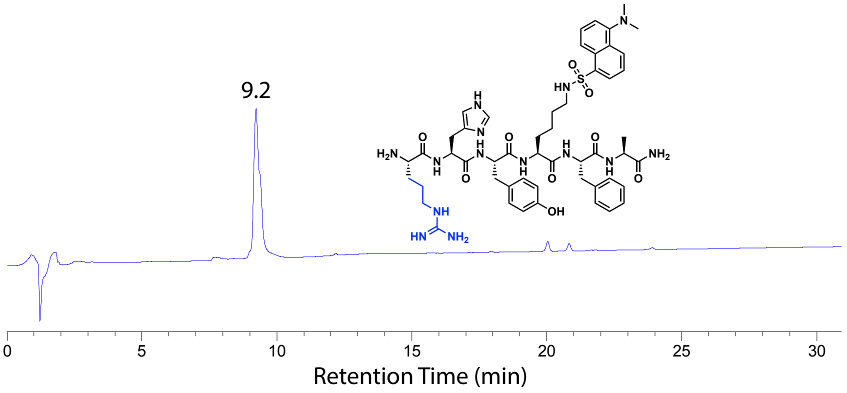

HPLC trace of Imine-HYK(Dansyl)FA-$CONH_2$

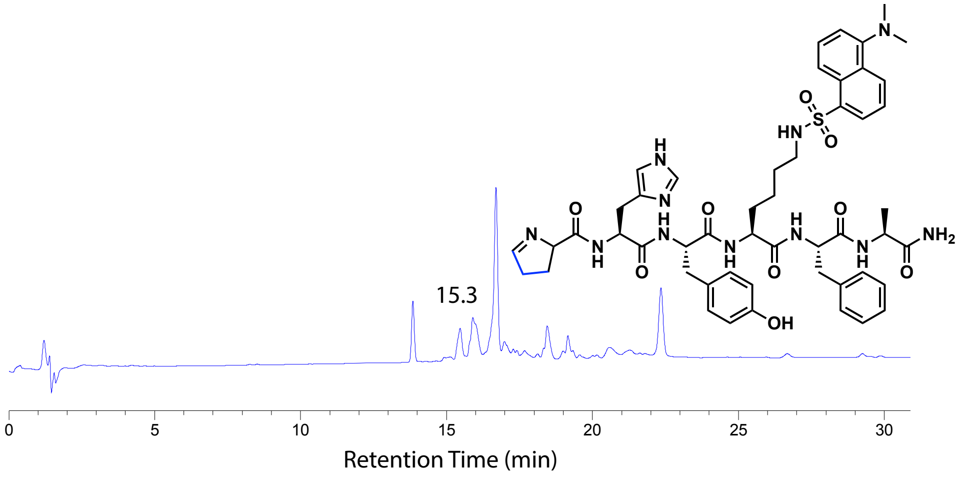

HRMS trace of H_2N-RHYK(Dansyl)FA-$CONH_2$

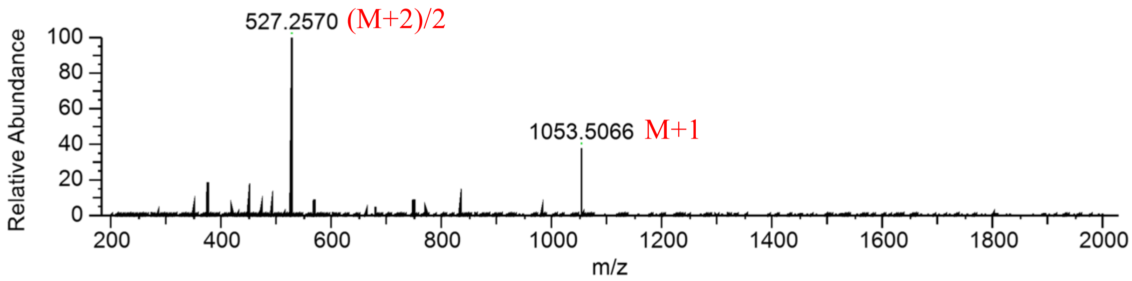

HRMS trace of Imine-HYK(Dansyl)FA-$CONH_2$

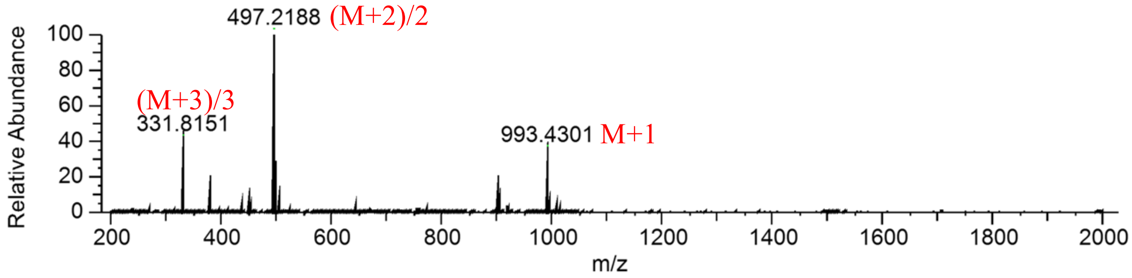

Aldehyde Formation of N-terminal Arginine peptide

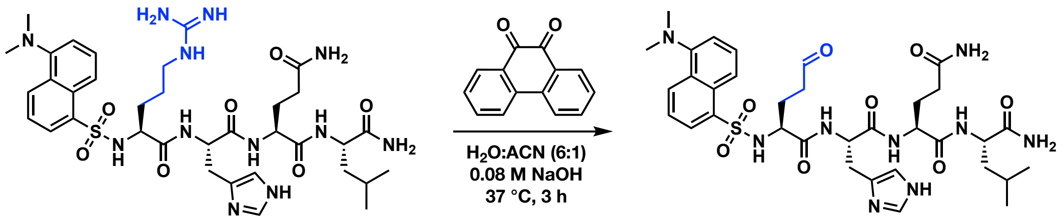

Dansyl-RHQL-$CONH_2$ (1 mg, 1.27 µmol, 1 equiv.) was dissolved in 500 µL H_2O in a 1/2" dram vial. Next, 9,10-phenanthrenequinone (0.80 mg, 3.82 µmol, 3 equiv.) from a freshly prepared stock solution in ACN was added in one portion before 240 µL H_2O was added to reach 900 µL total volume. Then 0.8 M NaOH (100 µL) was added from a freshly prepared stock solution yielding a total volume of 1 mL with a concentration of 0.08 M NaOH. The concentration of the peptide in solution was 1.27 mM. The vial was stirred at 37 °C for 3 h. The solution was filtered via syringe filtration then analyzed via HPLC method A to determine the percent conversion to cleaved product.

Dansyl-RHQL-$CONH_2$ peptide. LCMS *m/z* 393.1963 (calcd. [(M+2H^+)/2] = 393.1979), *m/z* 785.3853 (calcd. [M+H^+] = 785.3881), Purity: > 99 % (HPLC analysis at 220 nm). Retention time in HPLC: 9.2 min.

Dansyl-R(aldehyde)HQL-$CONH_2$ peptide. LCMS *m/z* 742.3348 (calcd. [M+H^+] = 742.3347), *m/z* 764.3163 (calcd. [M+Na^+] = 764.3166), (HPLC analysis at 220 nm). Retention time in HPLC: 11.9 min.

HPLC trace of Starting peptide Dansyl-RHQL-CONH$_2$

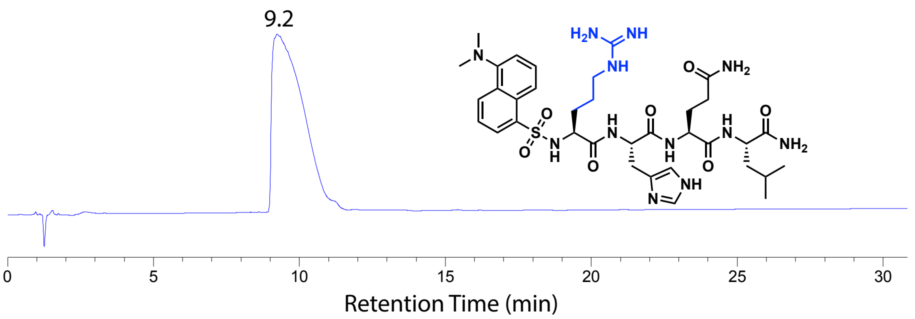

HPLC trace of Dansyl-R(aldehyde)HQL-CONH$_2$

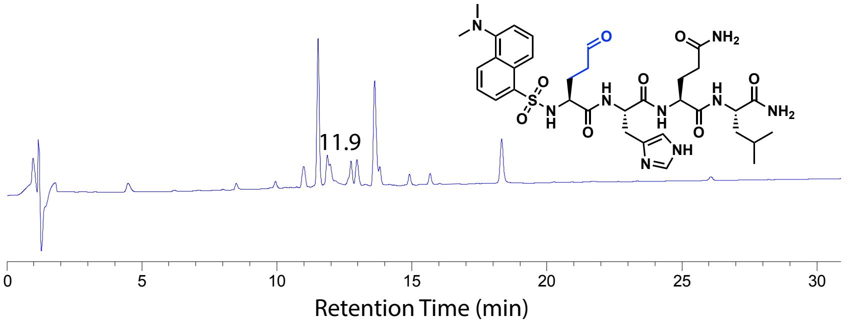

HRMS trace of Dansyl-RHQL-CONH$_2$

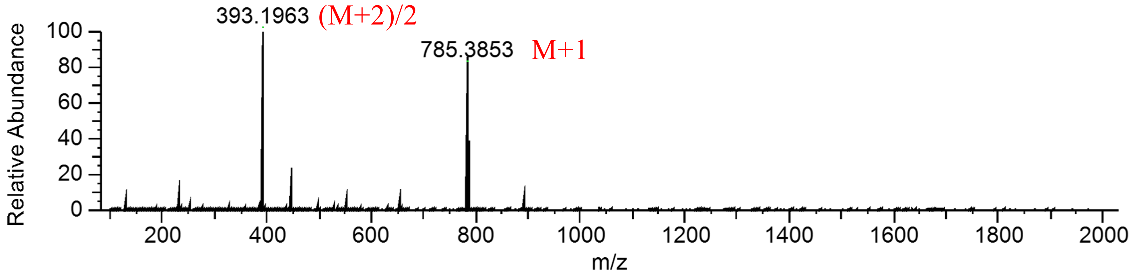

HRMS trace of Dansyl-R(aldehyde)HQL-CONH$_2$

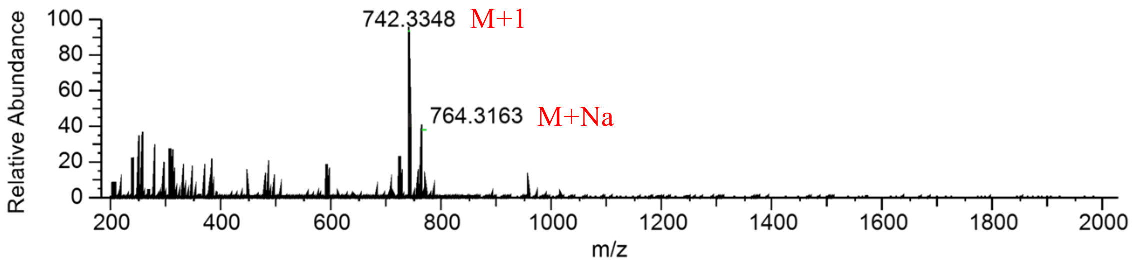

Aldehyde formation of middle Arginine peptide

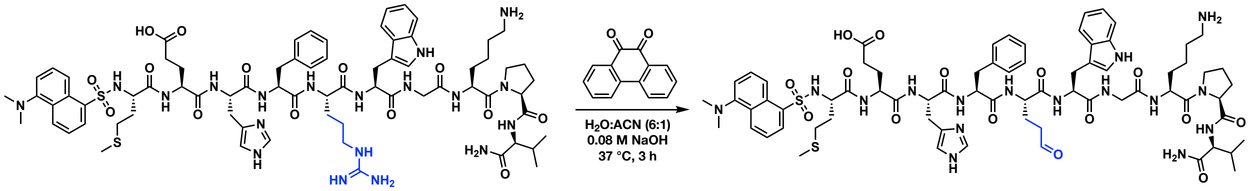

Dansyl-MEHFRWGKPV-CONH$_2$ (1 mg, 0.66 µmol, 1 equiv.) was dissolved in 500 µL H_2O in a 1/2" dram vial. Next, 9,10-phenanthrenequinone (0.41 mg, 1.98 µmol, 3 equiv.) from a freshly prepared stock solution in ACN was added in one portion before 318 µL H_2O was added to reach 900 µL total volume. Then 0.8 M NaOH (100 µL) was added from a freshly prepared stock solution yielding a total volume of 1 mL with a concentration of 0.08 M NaOH. The concentration of the peptide in solution was 0.66 mM. The vial was stirred at 37 °C for 3 h. The solution was filtered via syringe filtration then analyzed via HPLC method A to determine the percent conversion to cleaved product. Fractions were combined, lyophilized, and resubmitted to HPLC method B for characterization.

Dansyl-MEHFRWGKPV-CONH$_2$ peptide. LCMS *m/z* 506.9082 (calcd. [(M+3H$^+$)/3] = 506.9098), *m/z* 759.8586 (calcd. [(M+2H$^+$)/2] = 759.8608), *m/z* 1518.7096 (calcd. [M+H$^+$] = 1518.7138), Purity: > 99 % (HPLC analysis at 220 nm). Retention time in HPLC: 15.0 min.

Dansyl-MEHFR(aldehyde)WGKPV-CONH$_2$ peptide. LCMS *m/z* 738.3331 (calcd. [(M+2H$^+$)/2] = 738.3341), *m/z* 1475.6593 (calcd. [M+H$^+$] = 1475.6604), (HPLC analysis at 220 nm). Retention time in HPLC: 17.0 min.

HPLC trace of Dansyl-MEHFRWGKPV-CONH$_2$

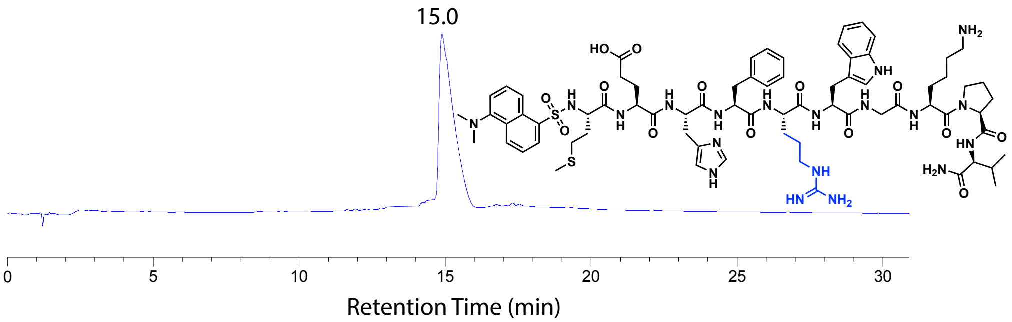

HPLC trace of Dansyl-MEHFR(aldehyde)WGKPV-CONH$_2$

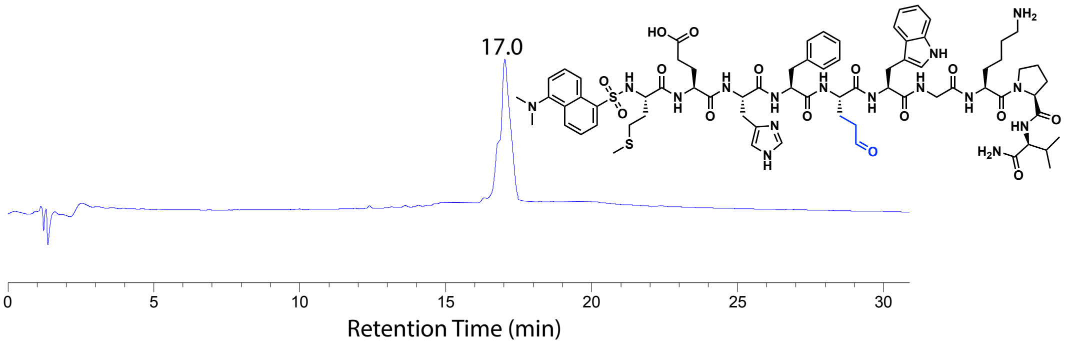

HRMS trace of Dansyl-MEHFRWGKPV-CONH$_2$

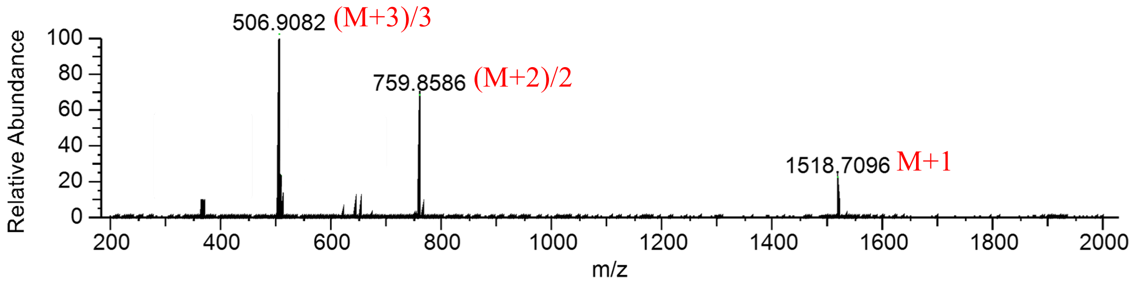

HRMS trace of Dansyl-MEHFR(aldehyde)WGKPV-CONH$_2$

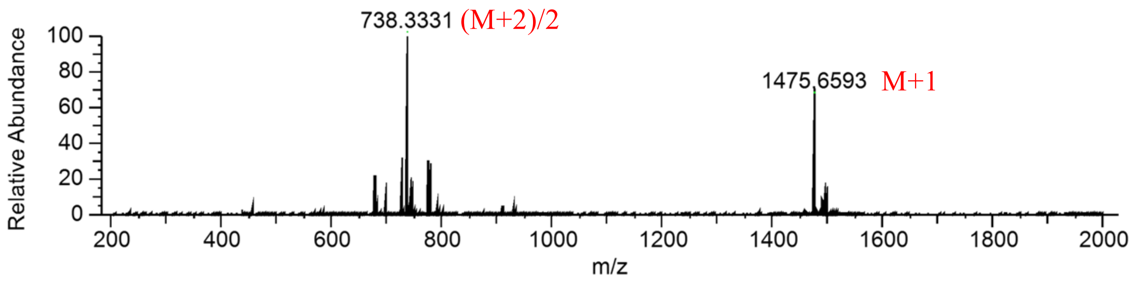

XI. Supplementary Figure 6. Cleavage of C+1 Peptides

Procedure for cleavage of C+1 Aspartate peptide 1b.

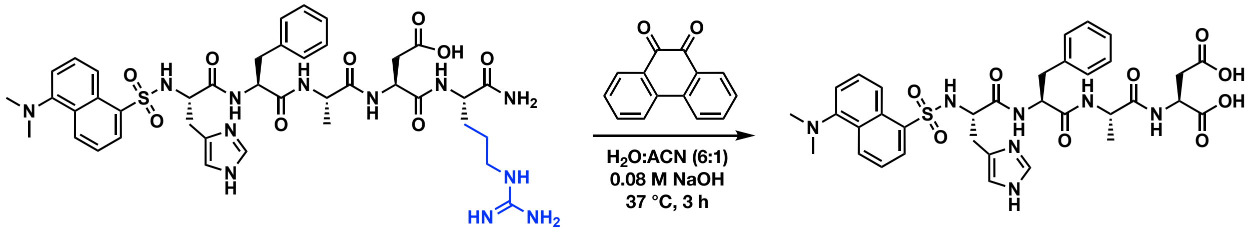

Dansyl-HFADR-CONH$_2$ (**1b**) (1 mg, 1.14 µmol, 1 equiv.) was dissolved in 500 µL H_2O in a 1/2" dram vial. Next, 9,10-phenanthrenequinone (0.71 mg, 3.42 µmol, 3 equiv.) from a freshly prepared stock solution in ACN was added in one portion before 258 µL H_2O was added to reach 900 µL total volume. Then 0.8 M NaOH (100 µL) was added from a freshly prepared stock solution yielding a total volume of 1 mL with 0.08 M NaOH. The concentration of the peptide in solution was 1.14 mM. The vial was stirred at 37 °C for 3 h. The solution was filtered via syringe filtration then analyzed via HPLC method B to determine the percent conversion to **2b** (>99%).

Dansyl-HFADR-CONH$_2$ peptide 1b. LCMS, *m/z* 439.1923 (calcd. [(M+2H$^+$)/2] = 439.1928), *m/z* 877.3772 (calcd. [M+H$^+$] = 877.3779) Purity: > 99 % (HPLC analysis at 220 nm). Retention time in HPLC: 7.8 min.

Dansyl-HFAD-CO₂H peptide 2b. LCMS *m/z* 361.1419 (calcd. [(*M*+2H$^+$)/2] = 361.1304), *m/z* 721.2763 (calcd. [*M*+H$^+$] = 721.2530), *m/z* 743.2576 (calcd. [*M*+Na$^+$] = 743.2349), (HPLC analysis at 220 nm). Retention time in HPLC: 12.0 min.

HPLC trace of Starting peptide 1b

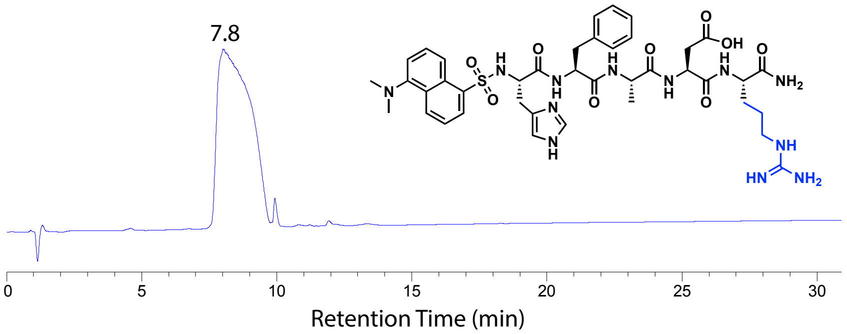

HPLC trace of Cleaved peptide 2b

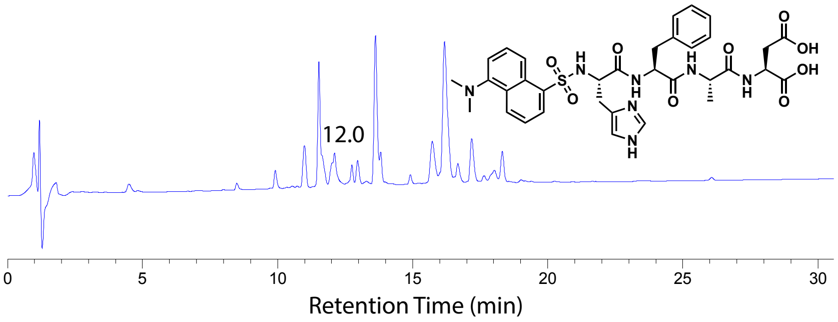

HRMS trace of 1b

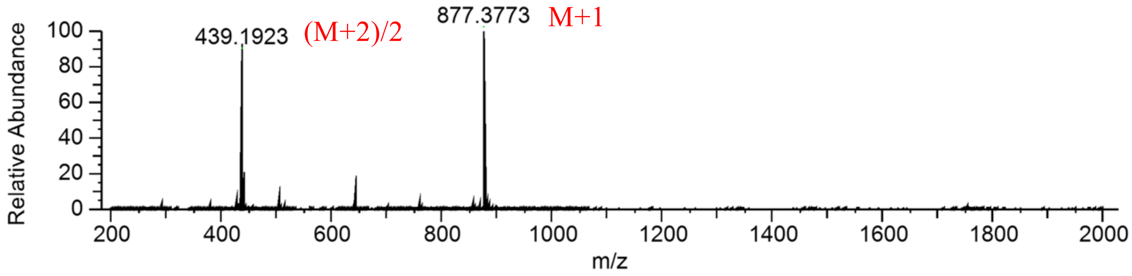

HRMS trace of 2b

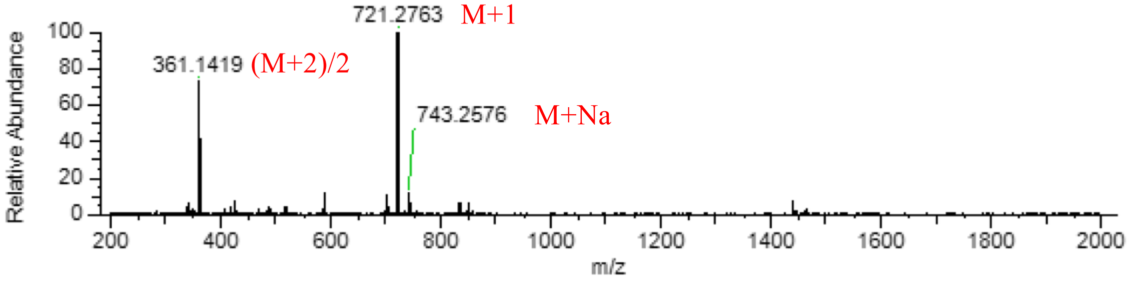

Procedure for cleavage of C+1 Histidine peptide 1c

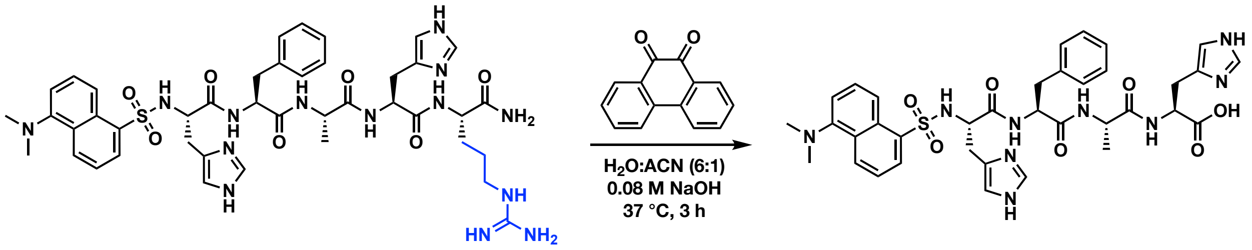

Dansyl-HFAHR-CONH$_2$ (**1c**) (1 mg, 1.11 µmol, 1 equiv.) was dissolved in 500 µL H_2O in a 1/2" dram vial. Next, 9,10-phenanthrenequinone (0.70 mg, 3.34 µmol, 3 equiv.) from a freshly prepared stock solution in ACN was added in one portion before 260 µL H_2O was added to reach 900 µL total volume. Then 0.8 M NaOH (100 µL) was added from a freshly prepared stock solution yielding a total volume of 1 mL with a concentration of 0.08 M NaOH. The concentration of the peptide in solution was 1.11 mM. The vial was stirred at 37 °C for 3 h. The solution was filtered via syringe filtration then analyzed via HPLC method B to determine the percent conversion to **2c** (>99%).

Dansyl-HFAHR-CONH$_2$ peptide 1c. LCMS *m/z* 300.4747 (calcd. $[(M{+}3H^+)/3]$ = 300.4751), *m/z* 450.2083 (calcd. $[(M{+}2H^+)/2]$ = 450.2088), *m/z* 899.4091 (calcd. $[M{+}H^+]$ = 899.4097), Purity: > 99 % (HPLC analysis at 220 nm). Retention time in HPLC: 7.1 min.

Dansyl-HFAH-CO$_2$H peptide 2c. LCMS *m/z* 372.1581 (calcd. $[(M{+}2H^+)/2]$ = 372.1464), *m/z* 372.6591 (calcd. $[(M{+}^{13}C{+}2H^+)/2]$ = 372.6464), *m/z* 743.3089 (calcd. $[M{+}H^+]$ = 743.2850), *m/z* 765.2901 (calcd. $[M{+}Na^+]$ = 765.2669), (HPLC analysis at 220 nm). Retention time in HPLC: 10.3 min.

HPLC trace of Starting peptide 1c

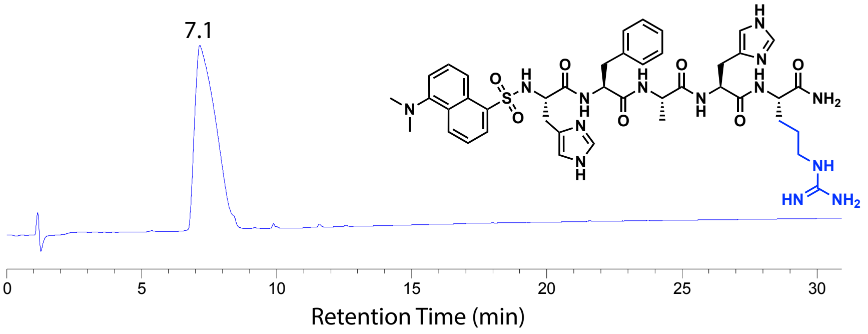

HPLC trace of Cleaved peptide 2c

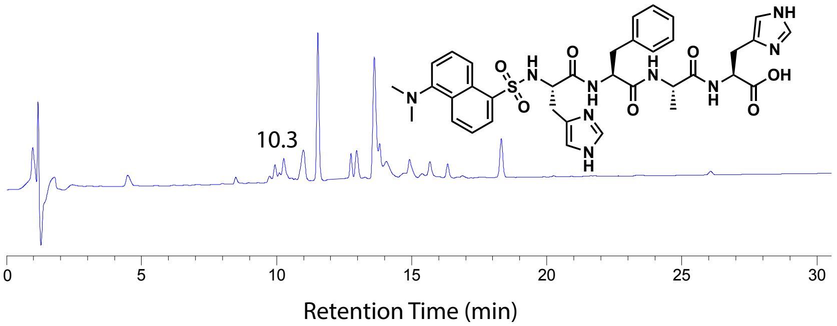

HRMS trace of 1c

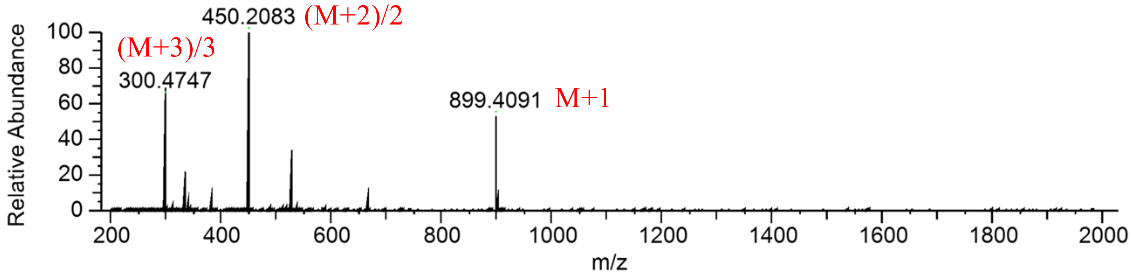

HRMS trace of 2c

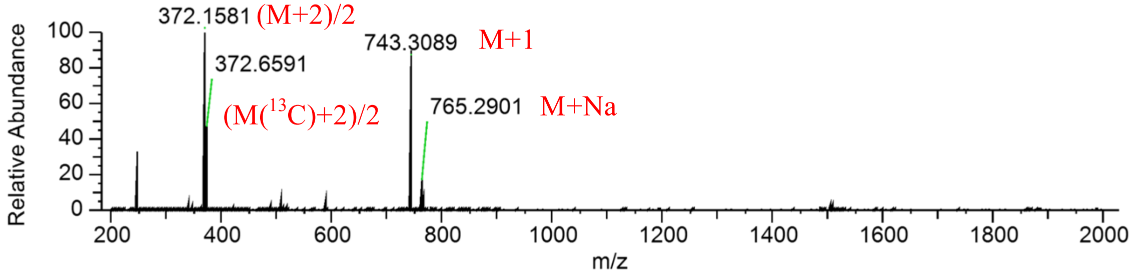

Procedure for cleavage of C+1 Serine peptide 1d

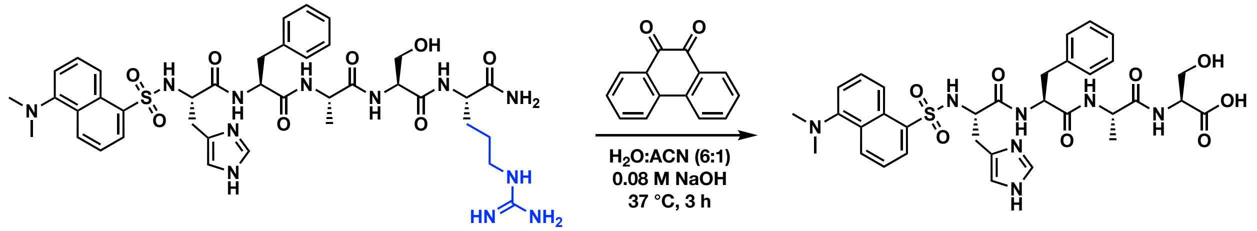

Dansyl-HFASR-CONH$_2$ (**1d**) (1 mg, 1.18 µmol, 1 equiv.) was dissolved in 500 µL H_2O in a 1/2" dram vial. Next, 9,10-phenanthrenequinone (0.74 mg, 3.54 µmol, 3 equiv.) from a freshly prepared stock solution in ACN was added in one portion before 252 µL H_2O was added to reach 900 µL total volume. Then 0.8 M NaOH (100 µL) was added from a freshly prepared stock solution yielding a total volume of 1 mL with a concentration of 0.08 M NaOH. The concentration of the peptide in solution was 1.18 µM.

The vial was stirred at 37 °C for 3 h. The solution was filtered via syringe filtration then analyzed via HPLC method B to determine the percent conversion to **2d** (>99%).

Dansyl-HFASR-CONH$_2$ peptide 1d. LCMS *m/z* 425.1949 (calcd. [(M+2H$^+$)/2] = 425.1954), *m/z* 849.3821 (calcd. [M+H$^+$] = 849.3831), *m/z* 850.3845 (calcd. [M+^{13}C+H$^+$] = 850.3831), Purity: > 99 % (HPLC analysis at 220 nm). Retention time in HPLC: 8.8 min.

Dansyl-HFAS-CO$_2$H peptide 2d. LCMS *m/z* 347.1443 (calcd. [(M+2H$^+$)/2] = 347.1329), *m/z* 693.2815 (calcd. [M+H$^+$] = 693.2659), *m/z* 715.2630 (calcd. [M+Na$^+$] = 715.2400), (HPLC analysis at 220 nm). Retention time in HPLC: 11.9 min.

HPLC trace of Starting peptide 1d

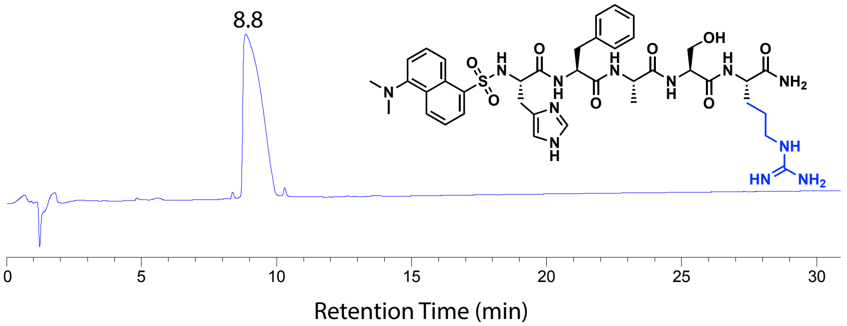

HPLC trace of Cleaved peptide 2d

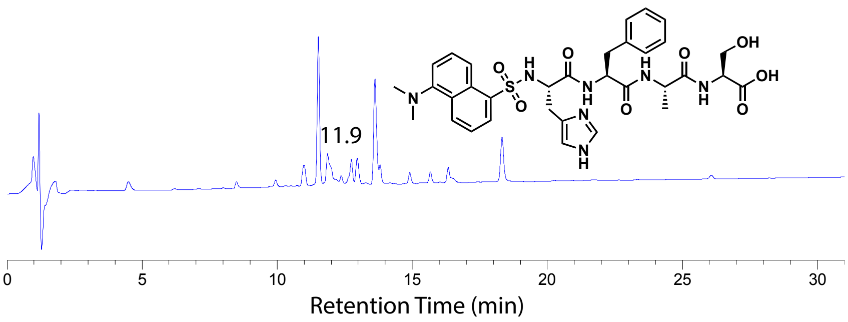

HRMS trace of 1d

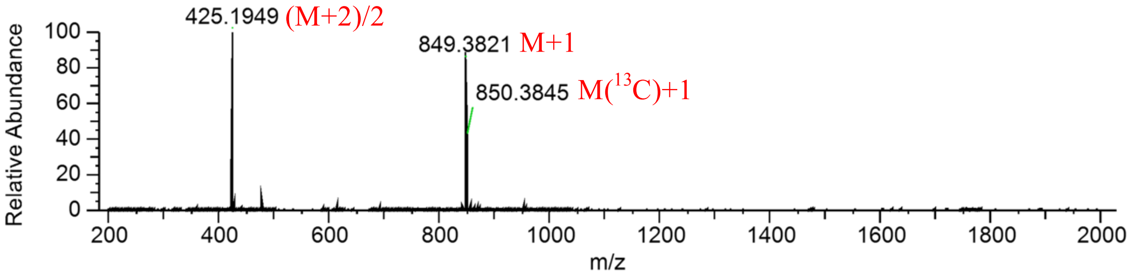

HRMS trace of 2d

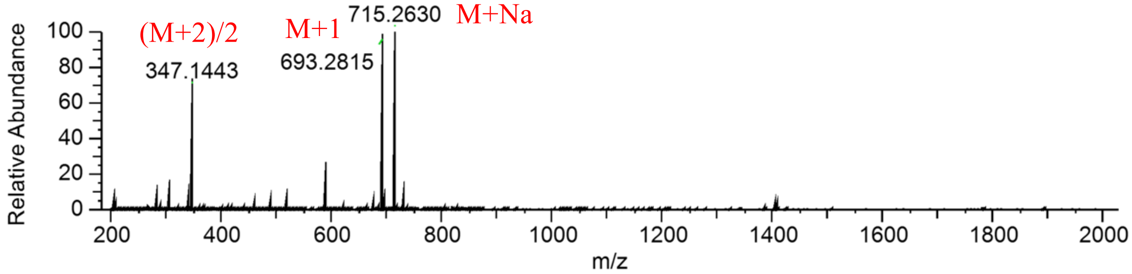

Procedure for cleavage of C+1 Valine peptide 1e

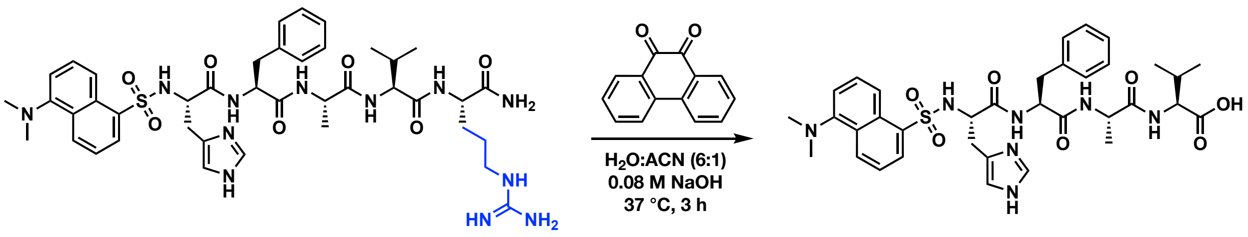

Dansyl-HFAVR-CONH$_2$ (**1e**) (1 mg, 1.16 µmol, 1 equiv.) was dissolved in 500 µL H_2O in a 1/2” dram vial. Next, 9,10-phenanthrenequinone (0.73 mg, 3.49 µmol, 3 equiv.) from a freshly prepared stock solution in ACN was added in one portion before 255 µL H_2O was added to reach 900 µL total volume. Then 0.8 M NaOH (100 µL) was added from a freshly prepared stock solution yielding a total volume of 1 mL with a concentration of 0.08 M NaOH. The concentration of the peptide in solution was 1.16 mM. The vial was stirred at 37 °C for 3 h. The solution was filtered via syringe filtration then analyzed via HPLC method B to determine the percent conversion to **2e** (>99%).

Dansyl-HFAVR-CONH$_2$ peptide 1e. LCMS *m/z* 431.2123 (calcd. [(M+2H$^+$)/2] = 431.2136), *m/z* 861.4171 (calcd. [M+H$^+$] = 861.4193), Purity: > 99 % (HPLC analysis at 220 nm). Retention time in HPLC: 10.4 min.
Dansyl-HFAV-CO$_2$H peptide 2e. LCMS *m/z* 353.1627 (calcd. [(M+2H$^+$)/2] = 353.1511), *m/z* 705.3179 (calcd. [M+H$^+$] = 705.2945), *m/z* 727.2993 (calcd. [M+Na$^+$] = 727.2764), (HPLC analysis at 220 nm). Retention time in HPLC: 12.8 min.

HPLC trace of Starting peptide 1e

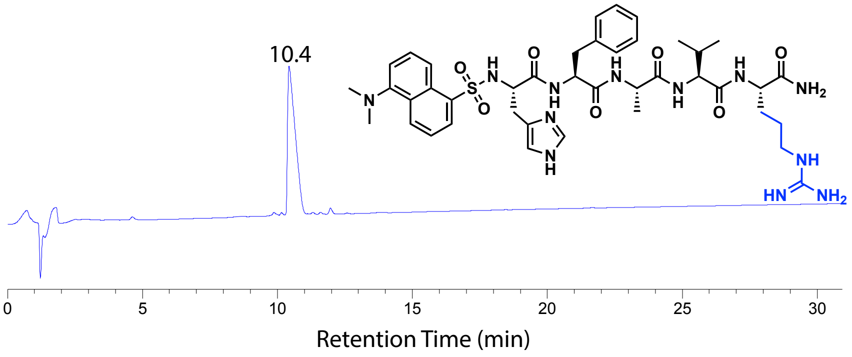

HPLC trace of Cleaved peptide 2e

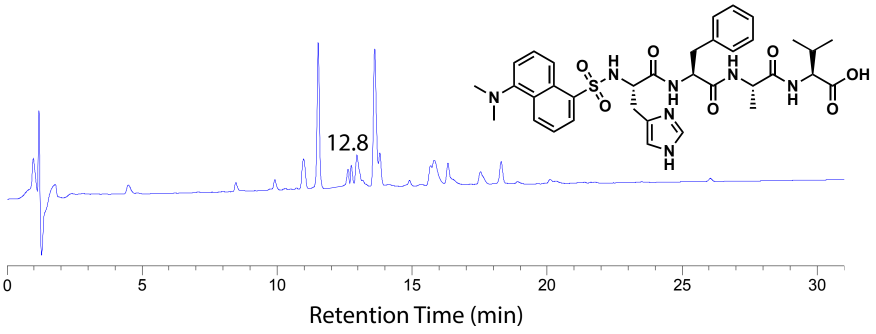

HRMS trace of 1e

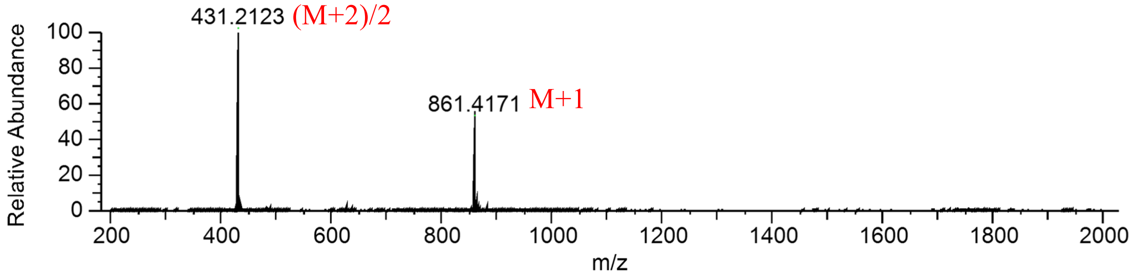

HRMS trace of 2e

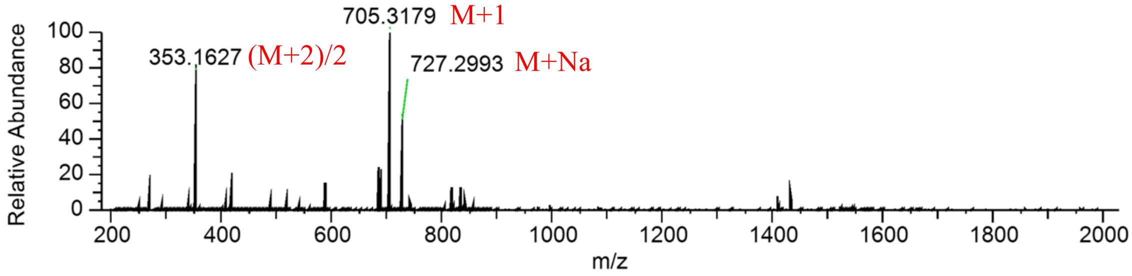

Procedure for cleavage of C+1 Tryptophan peptide 1f

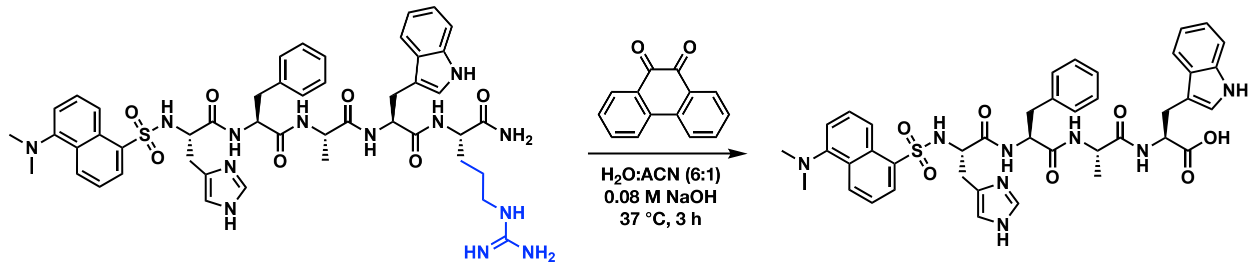

Dansyl-HFAWR-$CONH_2$ (**1f**) (1 mg, 1.06 μmol, 1 equiv.) was dissolved in 500 μL H_2O in a 1/2” dram vial. Next, 9,10-phenanthrenequinone (0.66 mg, 3.17 μmol, 3 equiv.) from a freshly prepared stock solution in ACN was added in one portion before 268 μL H_2O was added to reach 900 μL total volume. Then 0.8 M NaOH (100 μL) was added from a freshly prepared stock solution yielding a total volume of

1 mL with a concentration of 0.08 M NaOH. The concentration of the peptide in solution was 1.06 mM. The vial was stirred at 37 °C for 3 h. The solution was filtered via syringe filtration then analyzed via HPLC method A to determine the percent conversion to **2f** (>99%).

Dansyl-HFAWR-CONH$_2$ peptide 1f. LCMS *m/z* 474.7187 (calcd. [(*M*+2H$^+$)/2] = 474.7190), *m/z* 948.4298 (calcd. [*M*+H$^+$] = 948.4302), Purity: > 99 % (HPLC analysis at 220 nm). Retention time in HPLC: 10.6 min.

Dansyl-HFAW-CO$_2$H peptide 2f. LCMS *m/z* 793.3120 (calcd. [*M*+H$^+$] = 793.3131), (HPLC analysis at 220 nm). Retention time in HPLC: 13.0 min.

HPLC trace of Starting peptide 1f

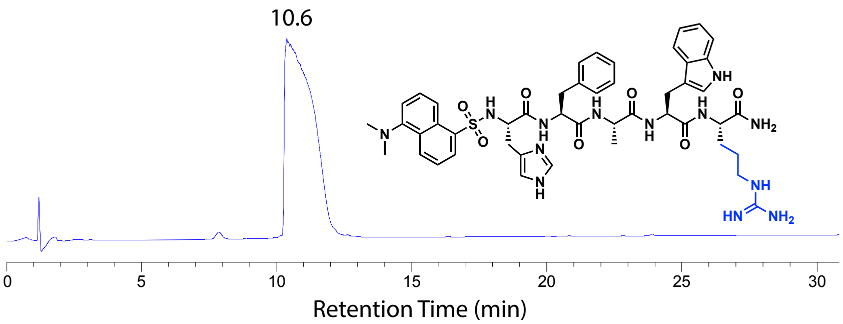

HPLC trace of Cleaved peptide 2f

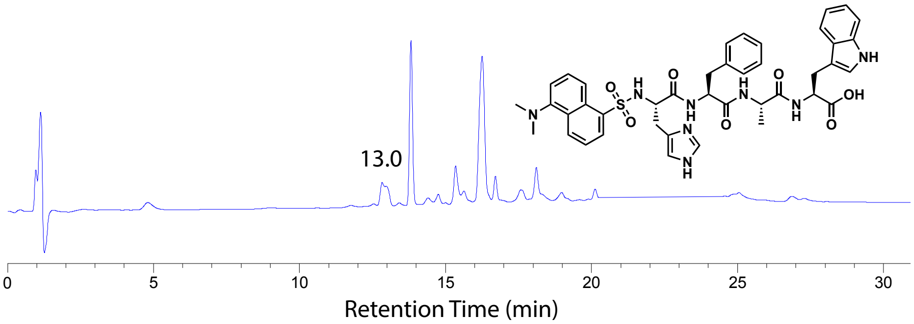

HRMS trace of 1f

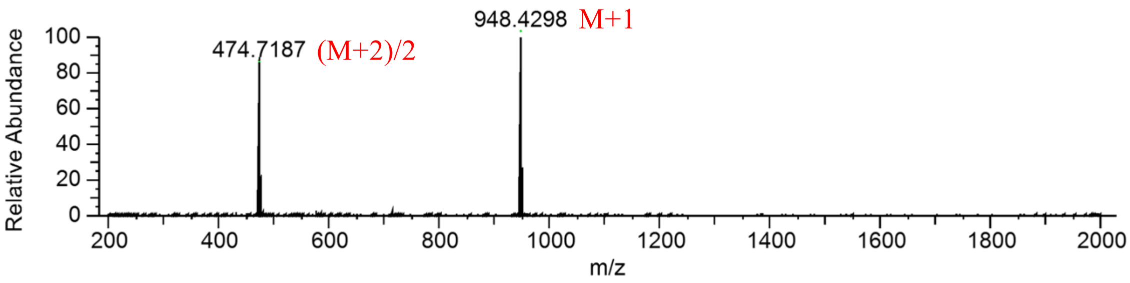

HRMS trace of 2f

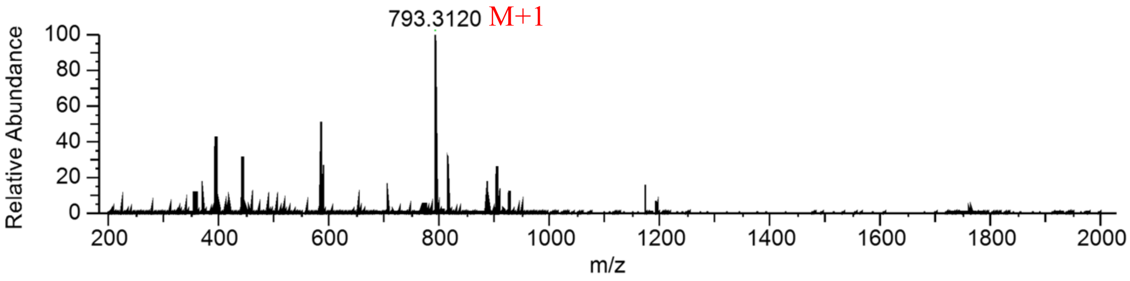

Procedure for cleavage of peptide 1g with C-terminal carboxylic acid

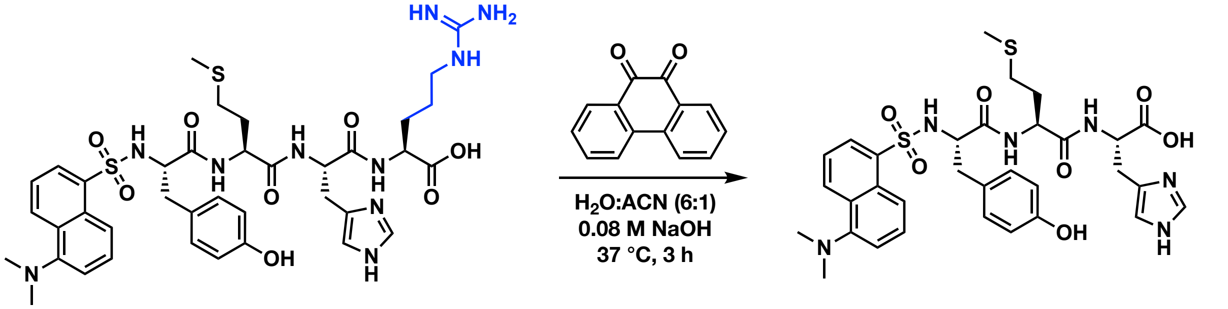

Dansyl-YMHR-CO_2H (**1g**) (1 mg, 1.19, 1 equiv.) was dissolved in 500 µL H_2O in a 1/2" dram vial. Next, 9,10-phenanthrenequinone (0.75 mg, 3.58 µmol, 3 equiv.) from a freshly prepared stock solution in ACN was added in one portion before 250 µL H_2O was added to reach 900 µL total volume. Then 0.8 M NaOH (100 µL) was added from a freshly prepared stock solution yielding a total volume of 1 mL with a concentration of 0.08 M NaOH. The concentration of the peptide in solution was 1.19 mM. The vial was stirred at 37 °C for 3 h. The solution was filtered via syringe filtration then analyzed via HPLC method B to determine the percent conversion to **2g** (>99%).

Dansyl-YMHR-CO_2H peptide 1g. LCMS *m/z* 420.1689 (calcd. [$(M+2H^+)/2$] = 420.1705), *m/z* 839.3299 (calcd. [$M+H^+$] = 839.3332), Purity: > 99 % (HPLC analysis at 220 nm). Retention time in HPLC: 9.1 min.

Dansyl-YMH-CO_2H peptide 2g. LCMS *m/z* 342.1195 (calcd. [$(M+2H^+)/2$] = 342.1199), *m/z* 683.2317 (calcd. [$M+H^+$] = 683.2321), (HPLC analysis at 220 nm). Retention time in HPLC: 11.6 min.

HPLC trace of Starting peptide 1g

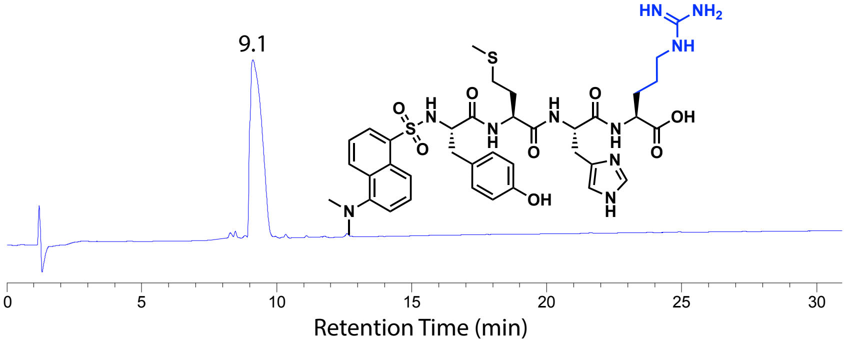

HPLC trace of Cleaved peptide 2g

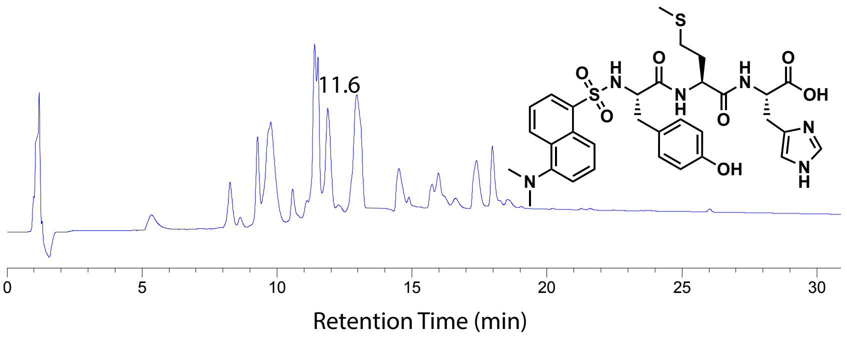

HRMS trace of 1g

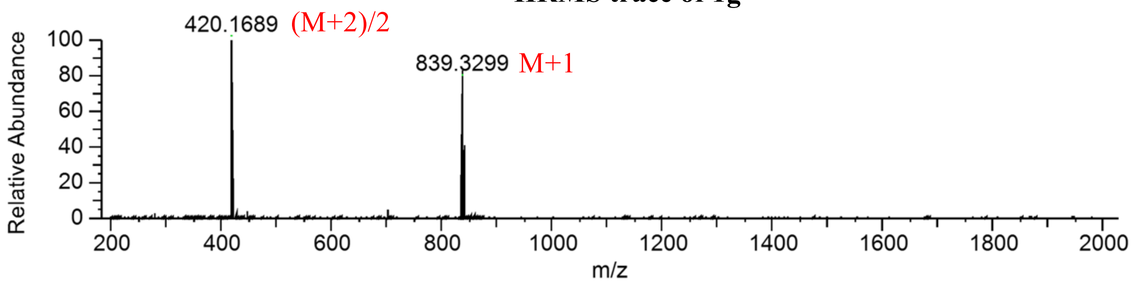

HRMS trace of 2g

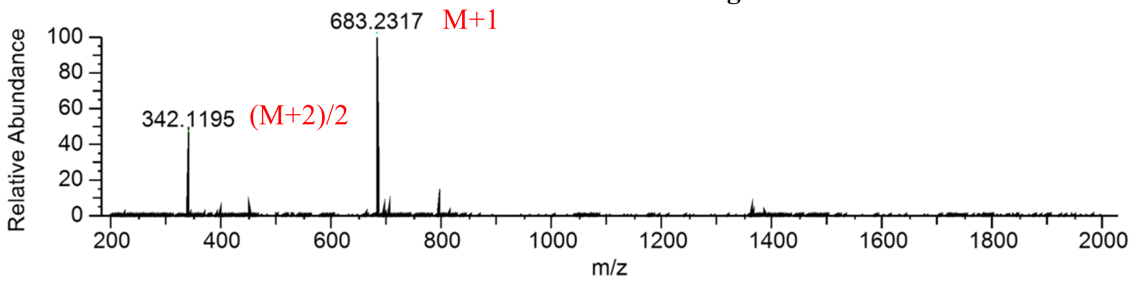

Procedure for cleavage of peptide 1h with free N-terminus

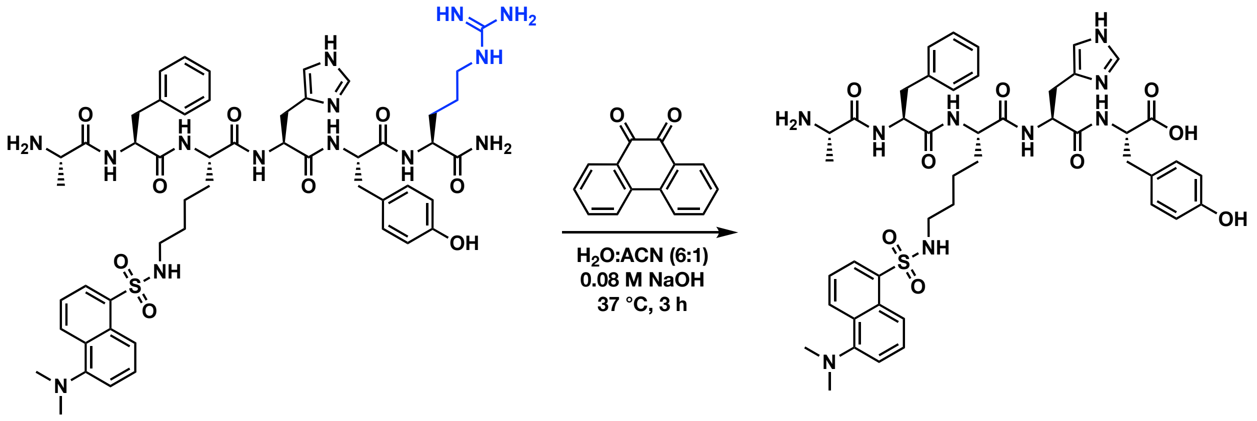

H_2N-AFK(dansyl)HYR-$CONH_2$ (**1h**) (1 mg, 0.95 µmol, 1 equiv.) was dissolved in 500 µL H_2O in a 1/2" dram vial. Next, 9,10-phenanthrenequinone (0.59 mg, 2.85 µmol, 3 equiv.) from a freshly prepared stock solution in ACN was added in one portion before 282 µL H_2O was added to reach 900 µL total volume. Then 0.8 M NaOH (100 µL) was added from a freshly prepared stock solution yielding a total volume of 1 mL with a concentration of 0.08 M NaOH. The concentration of the peptide in solution was 0.95 mM. The vial was stirred at 37 °C for 3 h. The solution was filtered via syringe filtration then analyzed via HPLC method A to determine the percent conversion to **2h** (>99%).

H_2N-AFK(dansyl)HYR-$CONH_2$ 1h. LCMS *m/z* 351.8409 (calcd. [(M+3H^+)/3] = 351.8416) *m/z* 527.2573 (calcd. [(M+2H^+)/2] = 527.2585), *m/z* 1053.5073 (calcd. [M+H^+] = 1053.5092), Purity: > 99 % (HPLC analysis at 220 nm). Retention time in HPLC: 10.4 min.

H_2N-AFK(dansyl)HY-CO_2H peptide 2h. LCMS *m/z* 449.2075 (calcd. [(M+2H^+)/2] = 449.1961), *m/z* 897.4078 (calcd. [M+H^+] = 897.3843), *m/z* 919.3890 (calcd. [M+Na^+] = 919.3663) (HPLC analysis at 220 nm). Retention time in HPLC: 13.2 min.

HPLC trace of Starting peptide 2h

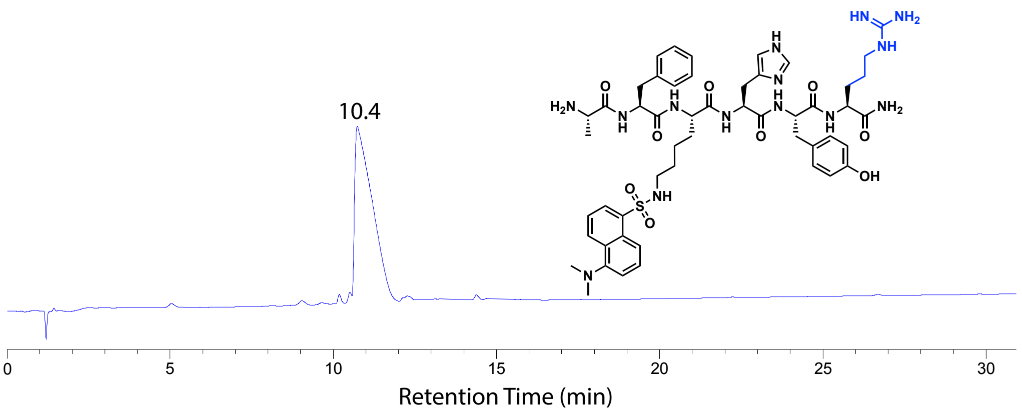

HPLC trace of Cleaved peptide 2h

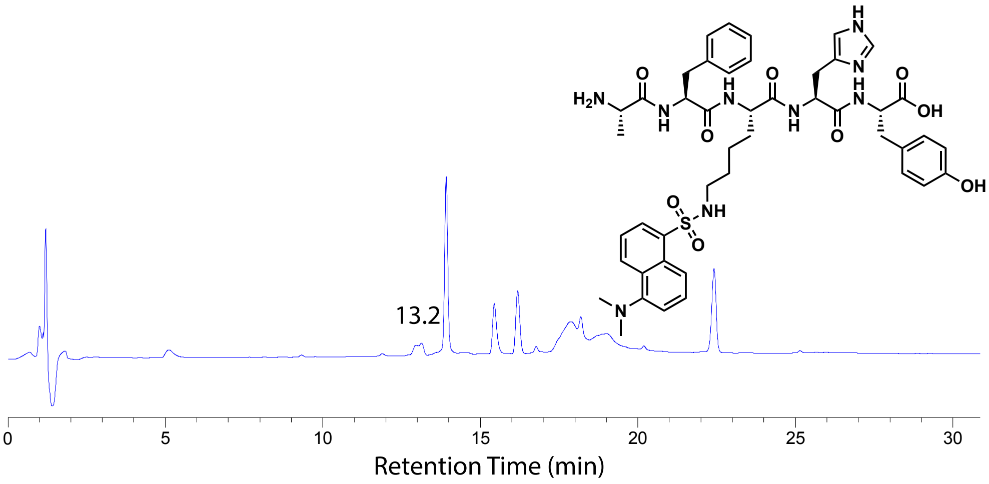

HRMS trace of 1h

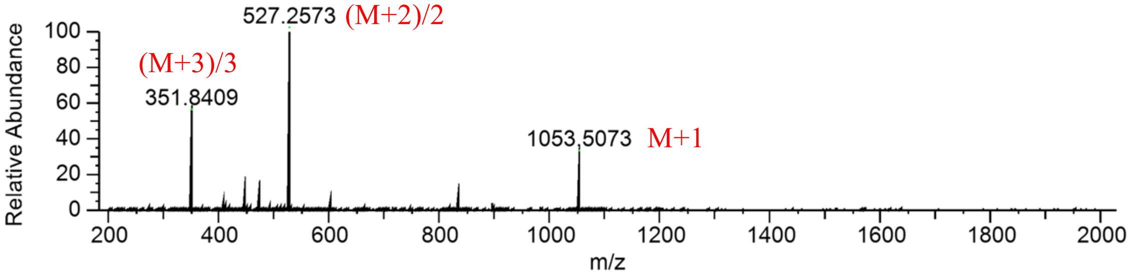

HRMS trace of 2h

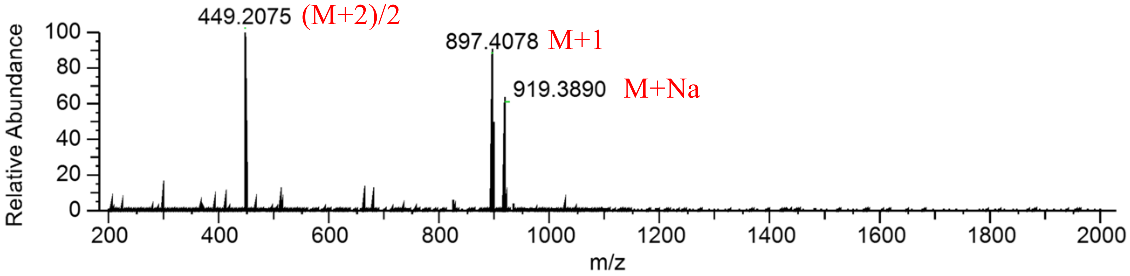

Procedure for cleavage of peptide 1i with two consecutive Arg at the C-terminus

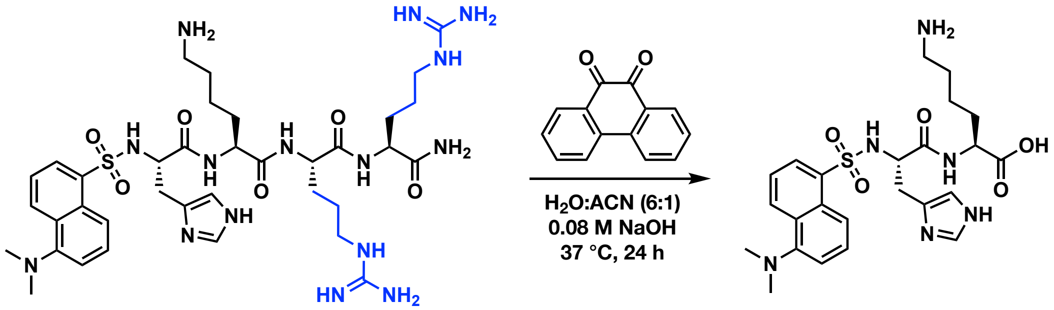

Dansyl-HKRR-CONH$_2$ (**1i**) (1 mg, 1.20 µmol, 1 equiv.) was dissolved in 500 µL H_2O in a 1/2" dram vial. Next, 9,10-phenanthrenequinone (1.51 mg, 7.25 µmol, 6 equiv.) from a freshly prepared stock solution in ACN was added in one portion before 282 µL H_2O was added to reach 900 µL total volume. Then 0.8 M NaOH (100 µL) was added from a freshly prepared stock solution yielding a total volume of 1 mL with a concentration of 0.08 M NaOH. The concentration of the peptide in solution was 1.2 mM. The vial was stirred at 37 °C for 3 h. The solution was filtered via syringe filtration then analyzed via HPLC method A to determine the percent conversion to **2i** (~ 77%).

Dansyl-HKRR-CONH$_2$ 1i. LCMS *m/z* 414.7220 (calcd. [(M+2H$^+$)/2] = 414.7246), *m/z* 828.4366 (calcd. [M+H$^+$] = 828.4415), Purity: > 99 % (HPLC analysis at 220 nm). Retention time in HPLC: 5.2 min.

Dansyl-HK-CO$_2$H peptide 2i. LCMS *m/z* 259.1147 (calcd. [(M+2H$^+$)/2] = 259.1155), *m/z* 517.2222 (calcd. [M+H$^+$] = 517.2233), (HPLC analysis at 220 nm). Retention time in HPLC: 9.6 min.

HPLC Trace of 1i

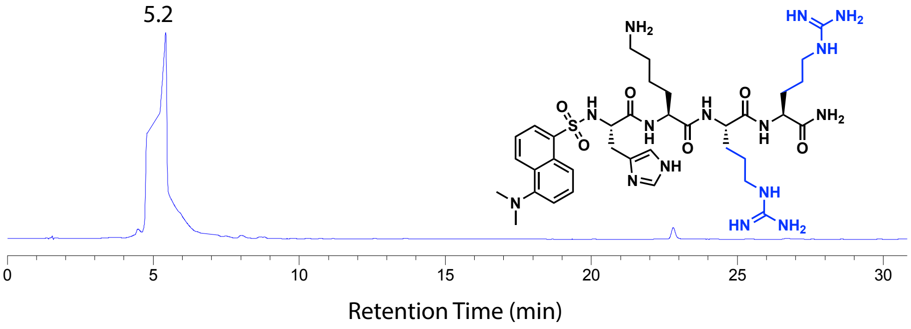

HPLC Trace of 2i

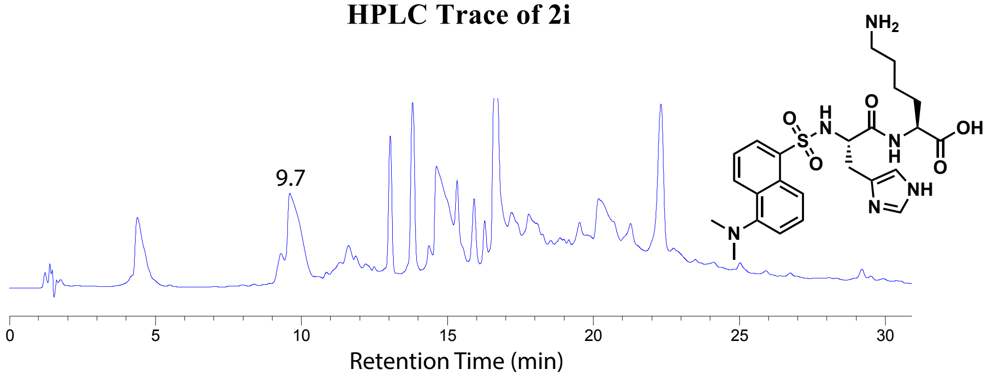

HRMS Trace of 1i

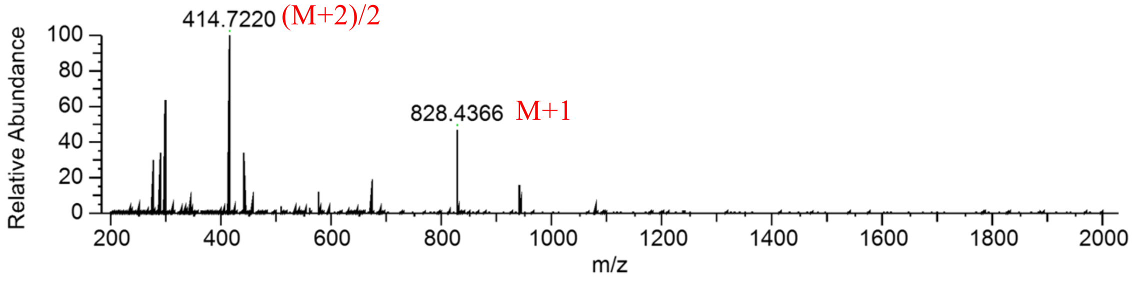

HRMS Trace of 2i

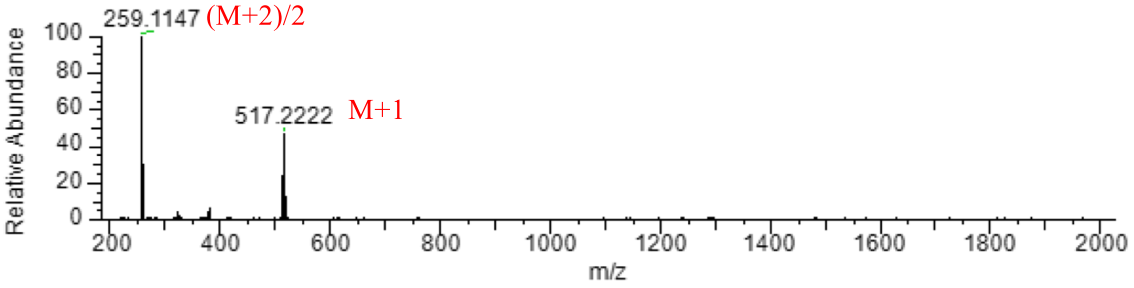

XII. Supplementary Figure 7. Computational Data

Computations were performed in Gaussian 10 through GaussView interface. Ab initio calculations were performed at B3LYP/6-311+G(d,p) level. Below are the reported geometry optimizations of model

peptides H_3N^+-GRG-CO_2^- and H_3N^+-GGR-CO_2^- for the starting aldehyde peptides (denoted as R(CHO)) and their respective pyrrolinium-like intermediates (denoted as R*). Hydroxide was added to the pyrrolinium-like intermediates to maintain the equivalent number of atoms for all structures. The grey, white, red, and blue balls represent the carbon, hydrogen, oxygen, and nitrogen atoms respectively.

(a) H_3N^+-GGR(CHO)-CO_2^- Ground State Minimizations –top view and side view

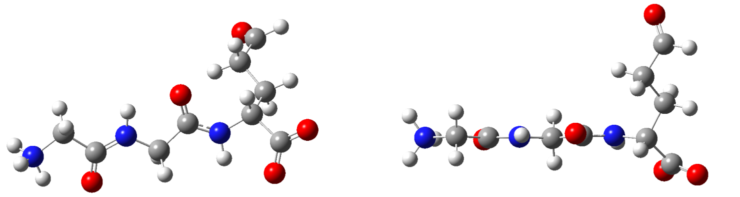

(b) H_3N^+-GGR*-CO_2^- Ground State Minimizations –top view and side view

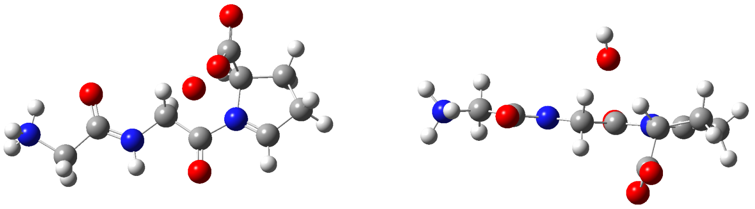

(c) H_3N^+-GRG(CHO)-CO_2^- Ground State Minimizations –top view and side view

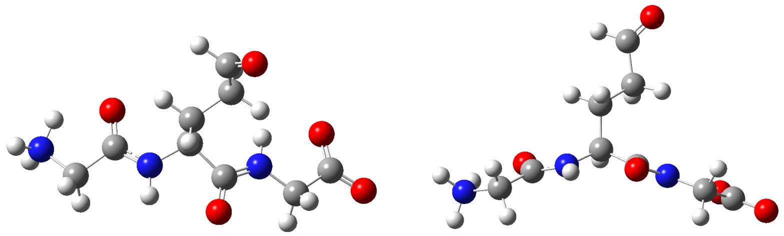

(d) H_3N^+-GR*G-CO_2^- Ground State Minimizations –top view and side view

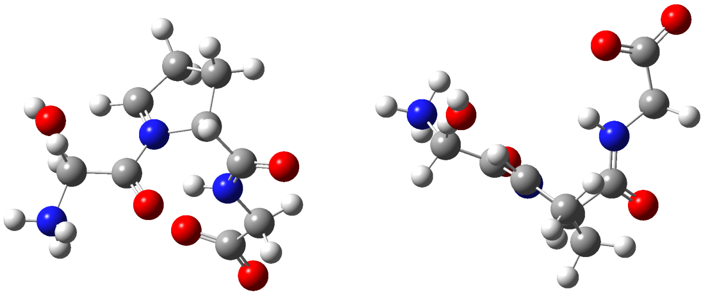

Geometry Optimizations

Cartesian coordinates and total energies for geometry optimized model peptides obtained at B3LYP/6-311+G(d, p) level.

Geometry optimized H_3N^+-GGR-CO_2^-

Center Atomic Atomic Coordinates (Angstroms)

Number	Number	Type	X	Y	Z
1	7	0	-6.189643	0.471901	0.041437
2	1	0	-5.987028	-0.048741	-0.835821
3	6	0	-4.865168	0.648805	0.716990
4	1	0	-6.635294	1.359005	-0.195724
5	1	0	-6.849624	-0.071593	0.598986
6	6	0	-3.823166	-0.032985	-0.184413
7	1	0	-4.656263	1.710637	0.827192
8	1	0	-4.897401	0.188210	1.702263
9	8	0	-4.196010	-0.571810	-1.230184
10	7	0	-2.564501	0.009918	0.245229
11	1	0	-2.307202	0.472610	1.112716
12	6	0	-1.455759	-0.588420	-0.474985
13	6	0	-0.167159	-0.300958	0.306224
14	1	0	-1.596935	-1.669071	-0.570655
15	1	0	-1.382309	-0.173953	-1.483808
16	8	0	-0.207703	0.323978	1.369524
17	7	0	0.950714	-0.769428	-0.267250
18	1	0	0.879083	-1.420731	-1.044690
19	6	0	2.267723	-0.804564	0.367173
20	6	0	2.945789	-2.173242	0.034888
21	1	0	2.130674	-0.735173	1.448001
22	8	0	2.437936	-2.839481	-0.904102
23	8	0	3.951553	-2.462028	0.723887
24	6	0	3.180075	0.344218	-0.104706
25	1	0	3.302665	0.273605	-1.190995
26	1	0	4.164286	0.184207	0.343511
27	6	0	2.661210	1.746421	0.264076
28	1	0	1.700583	1.961255	-0.207045
29	1	0	2.532060	1.803349	1.352741
30	6	0	3.649485	2.810855	-0.117742
31	8	0	3.415140	3.746986	-0.854129
32	1	0	4.661191	2.697693	0.322412

RHF energy = -892.701945 Hartree

Geometry optimized H_3N^+-GGR*-CO_2^-

Center Number	Atomic Number	Atomic Type	Coordinates (Angstroms) X	Y	Z
1	7	0	-5.523500	-0.409329	-0.175142
2	1	0	-5.084143	-1.294867	0.093339
3	6	0	-4.446725	0.655130	-0.185387
4	1	0	-5.945066	-0.531474	-1.090024
5	1	0	-6.265753	-0.196834	0.482495
6	6	0	-3.108796	-0.059914	-0.043972
7	1	0	-4.507849	1.208689	-1.107577
8	1	0	-4.608632	1.317748	0.650085

9	8	0	-3.090186	-1.289325	0.161482
10	7	0	-2.027607	0.695402	-0.140215
11	1	0	-2.096179	1.679769	-0.319664
12	6	0	-0.690712	0.121721	0.017143
13	6	0	0.305294	1.224766	-0.124469
14	1	0	-0.571043	-0.335953	1.004256
15	1	0	-0.509482	-0.621481	-0.747963
16	8	0	0.033422	2.388235	-0.348319
17	6	0	2.149837	-0.559578	0.324754
18	6	0	3.969073	1.072571	0.180885
19	6	0	3.605981	-0.303765	0.791685
20	1	0	1.538506	-0.982014	1.114243
21	1	0	4.472040	0.985991	-0.779863
22	1	0	4.585086	1.689996	0.820033
23	1	0	4.264525	-1.089972	0.461138
24	1	0	3.635276	-0.245767	1.870576
25	7	0	1.674865	0.848950	0.055898
26	6	0	2.638321	1.697049	-0.047695
27	1	0	2.447706	2.725545	-0.281137
28	6	0	2.090392	-1.379302	-0.970204
29	8	0	2.443745	-2.587364	-0.847252
30	8	0	1.705902	-0.797572	-2.027070
31	8	0	0.235085	-0.940746	2.619248
32	1	0	0.078974	-1.252427	3.525131

RHF energy = -892.660197 Hartree

Geometry optimized H_3N^+-GRG-CO_2^-

Center Number	Atomic Number	Atomic Type	Coordinates (Angstroms) X	Y	Z
1	7	0	-5.278673	-1.301913	-0.511472
2	1	0	-4.957992	-0.813135	-1.353228
3	6	0	-4.140670	-1.287334	0.488802
4	1	0	-6.105235	-0.832002	-0.157837
5	1	0	-5.545284	-2.245089	-0.772507
6	6	0	-2.948562	-0.634339	-0.200869
7	1	0	-4.444038	-0.715227	1.351054
8	1	0	-3.918740	-2.300676	0.781387
9	8	0	-3.077883	-0.199411	-1.362474
10	7	0	-1.830494	-0.567159	0.504666
11	1	0	-1.781067	-0.919559	1.444304
12	6	0	-0.608412	0.057264	-0.011409
13	6	0	0.553598	-0.597915	0.724383
14	1	0	-0.549345	-0.126695	-1.072762
15	8	0	0.429559	-0.891174	1.936349
16	6	0	-0.643149	1.577533	0.270438

17	1	0	-0.711979	1.729891	1.340871
18	1	0	-1.551275	1.961242	-0.178918
19	6	0	0.577443	2.327672	-0.304250
20	1	0	1.498591	2.037652	0.179829
21	1	0	0.664918	2.111531	-1.367626
22	6	0	0.392507	3.811170	-0.168113
23	8	0	1.227355	4.568688	0.316184
24	1	0	-0.542474	4.212986	-0.540001
25	7	0	1.662763	-0.792748	0.034119
26	1	0	1.726532	-0.594229	-0.947956
27	6	0	2.880933	-1.351680	0.611683
28	6	0	3.951877	-1.468438	-0.477588
29	1	0	3.251166	-0.718040	1.407792
30	1	0	2.689402	-2.333253	1.027085
31	8	0	3.638776	-1.101005	-1.651246
32	8	0	5.070725	-1.930379	-0.105205

RHF energy = -892.702269 Hartree

Geometry optimized H_3N^+-GR*G-CO_2^-

Center Number	Atomic Number	Atomic Type	Coordinates (Angstroms) X	Y	Z
1	7	0	2.933102	-2.238615	-1.487680
2	1	0	2.912666	-1.927763	-2.455502
3	6	0	2.815319	-1.081190	-0.521456
4	1	0	2.180062	-2.909614	-1.363347
5	1	0	3.807650	-2.732909	-1.335311
6	6	0	1.636615	-0.249193	-0.949056
7	1	0	2.742792	-1.456748	0.498709
8	1	0	3.716821	-0.488157	-0.608197
9	8	0	1.118045	-0.341227	-2.041382
10	6	0	0.279894	1.832652	-0.557875
11	6	0	0.807427	2.035914	1.818610
12	6	0	0.376151	2.872292	0.589972
13	1	0	0.672903	2.197754	-1.491087
14	1	0	-0.035589	1.716996	2.427971
15	1	0	1.499164	2.549072	2.473476
16	1	0	-0.560178	3.384746	0.742795
17	1	0	1.138988	3.597981	0.348411
18	6	0	1.442811	0.822168	1.233772
19	1	0	2.027389	0.019433	1.794265
20	7	0	1.165269	0.734761	-0.044197
21	6	0	-1.154775	1.375335	-0.786654
22	8	0	-1.889179	2.120043	-1.471732
23	7	0	-1.570582	0.261842	-0.211041
24	1	0	-0.979386	-0.337068	0.332950
25	6	0	-2.939251	-0.236518	-0.316714
26	6	0	-3.047538	-1.544859	0.475073
27	1	0	-3.641012	0.483935	0.084019

28	1	0	-3.200542	-0.422076	-1.351025
29	8	0	-1.996300	-1.962933	1.052512
30	8	0	-4.184987	-2.098678	0.482631
31	8	0	2.691791	-1.267242	2.343501
32	1	0	2.808888	-1.540817	3.264029

RHF energy = -892.634878 Hartree

XIII. Supplementary Figure 8. Procedure for Methyl Ester Formation

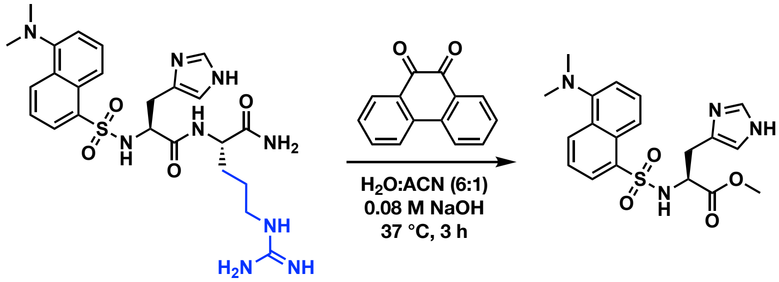

Dansyl-HR-$CONH_2$ (10 mg, 1.8 µmol, 1 equiv.) was dissolved in 5 mL anhydrous MeOH in a 1.5" dram vial. Next, 9,10-phenanthrenequinone (1.15 mg, 5.4 µmol, 3 equiv.) from a freshly prepared stock solution in ACN was added in one portion before 4.85 mL anhydrous MeOH was added to reach 10 mL volume. Then NaOMe (50 equiv.) was added. The concentration of the peptide in solution was 1.8 mM. The vial was stirred at 37 °C for 3 h. The solution was filtered via syringe filtration then analyzed via HPLC method B to determine the percent conversion to Dansyl-H-CO_2Me (90%). Fractions were combined and lyophilized revealing a fluffy white solid for characterization.

Dansyl-HR-$CONH_2$ peptide. LCMS, *m/z* 272.6268 (calcd. $[(M{+}2H^+)/2]$ = 272.6266), *m/z* 544.260 (calcd. $[M{+}H^+]$ = 544.2454) Purity: > 99 % (HPLC analysis at 220 nm). Retention time in HPLC: 5.9 min.

Dansyl-H-CO_2Me peptide. LCMS *m/z* 202.0751 (calcd. $[(M{+}2H^+)/2]$ = 202.0759), *m/z* 403.1426 (calcd. $[M{+}H^+]$ = 403.1440), (HPLC analysis at 220 nm). Retention time in HPLC: 10.2 min. Purity >90%.
Cleavage to carboxylic acid can be seen at 8.8 min.

^{1}H NMR (400 MHz, CD_3OD, ppm) δ: 8.52 (1H, d, *J* = 8.5 Hz), 8.44 (1H, s), 8.18 (1H, d, *J* = 8.6 Hz), 8.11 (1H, d, *J* = 7.4 Hz), 7.52 (2H, t, *J* = 8.0 Hz), 7.25 (2H, m), 6.58 (1H, s), 4.05 (1H, q, *J* = 4.7 Hz), 3.45 (3H, s), 2.90-2.85 (2H, overlapping), 2.86 (6H, s).

^{13}C NMR (101 MHz, CD_3OD, ppm) δ: 172.8, 1153.0, 136.8, 135.7, 131.3, 130.8, 130.4, 128.9, 128.6, 124.2, 120.8, 116.4, 57.4, 52.4, 45.8, 30.9.

HPLC Starting peptide (Dansyl-HR-$CONH_2$)

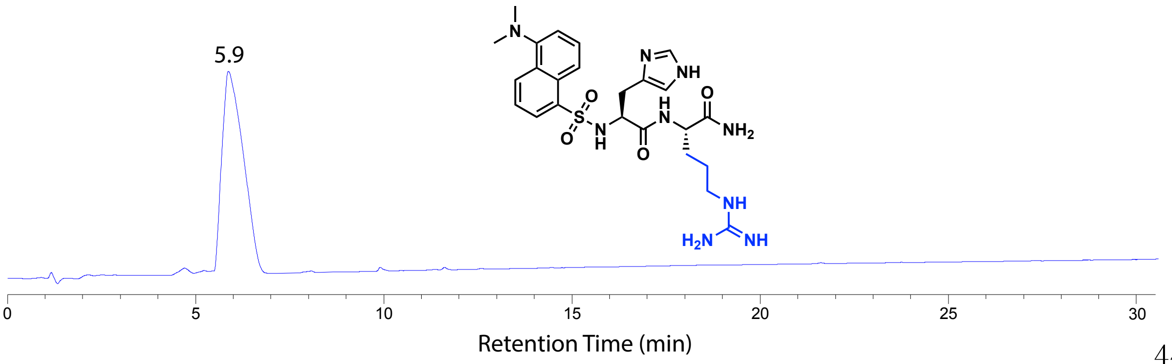

HPLC Ester peptide (Dansyl-H-CO_2Me)

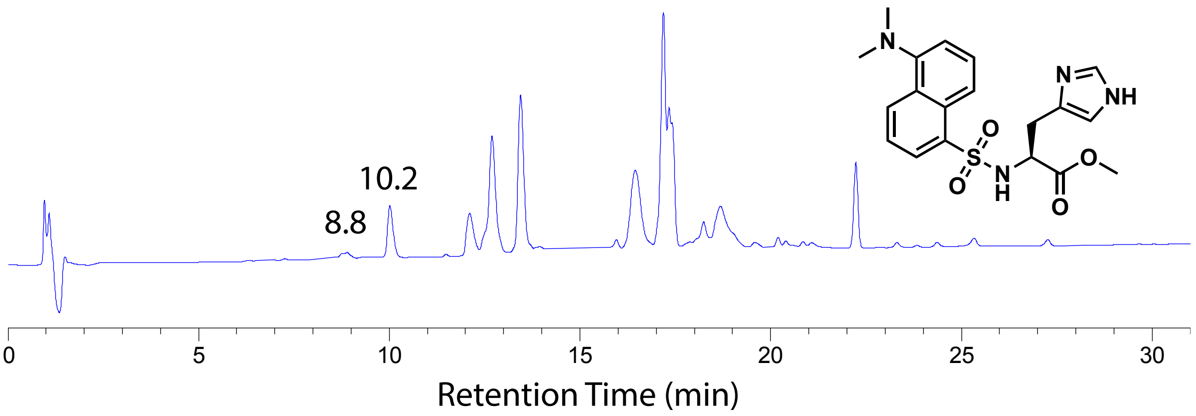

HRMS of Dansyl-HR-$CONH_2$

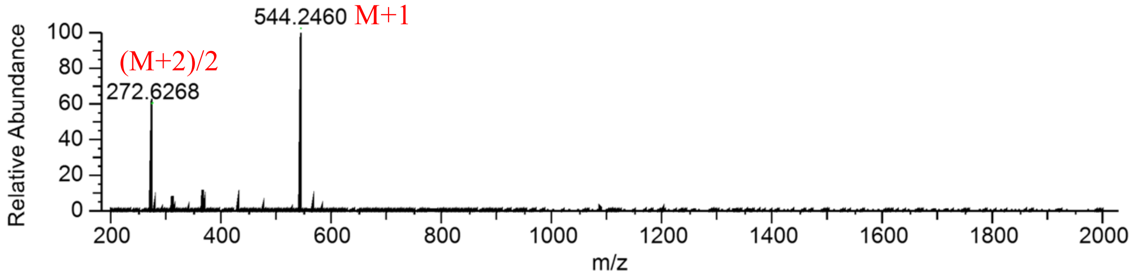

HRMS of Dansyl-H-CO_2Me

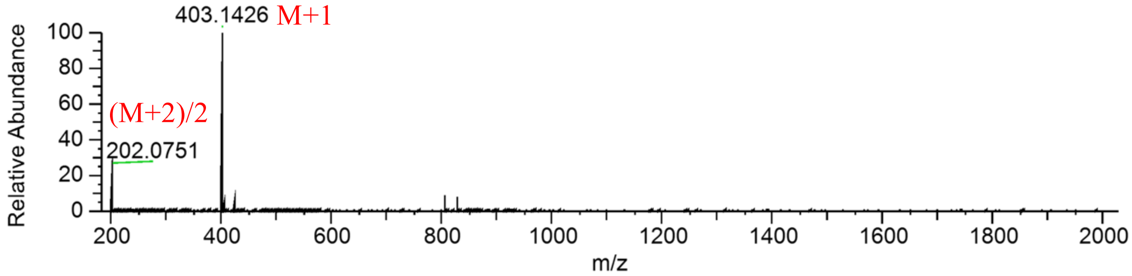

^{1}H NMR Dansyl-H-CO_2Me

CD_3OD (400 MHz)

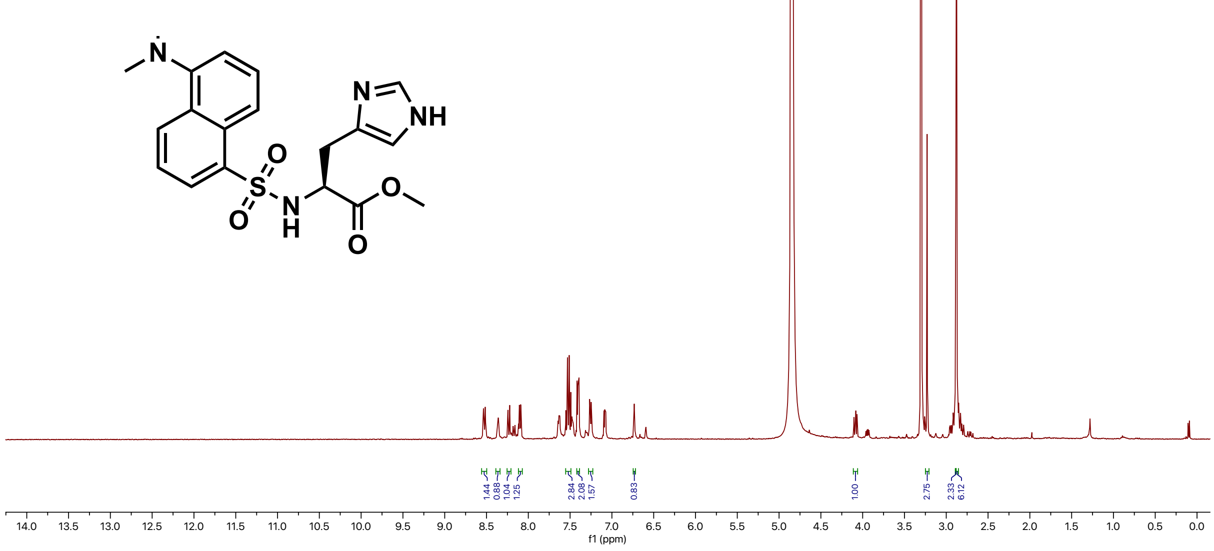

^{13}C NMR Dansyl-H-CO_2Me

CD_3OD (101 MHz)

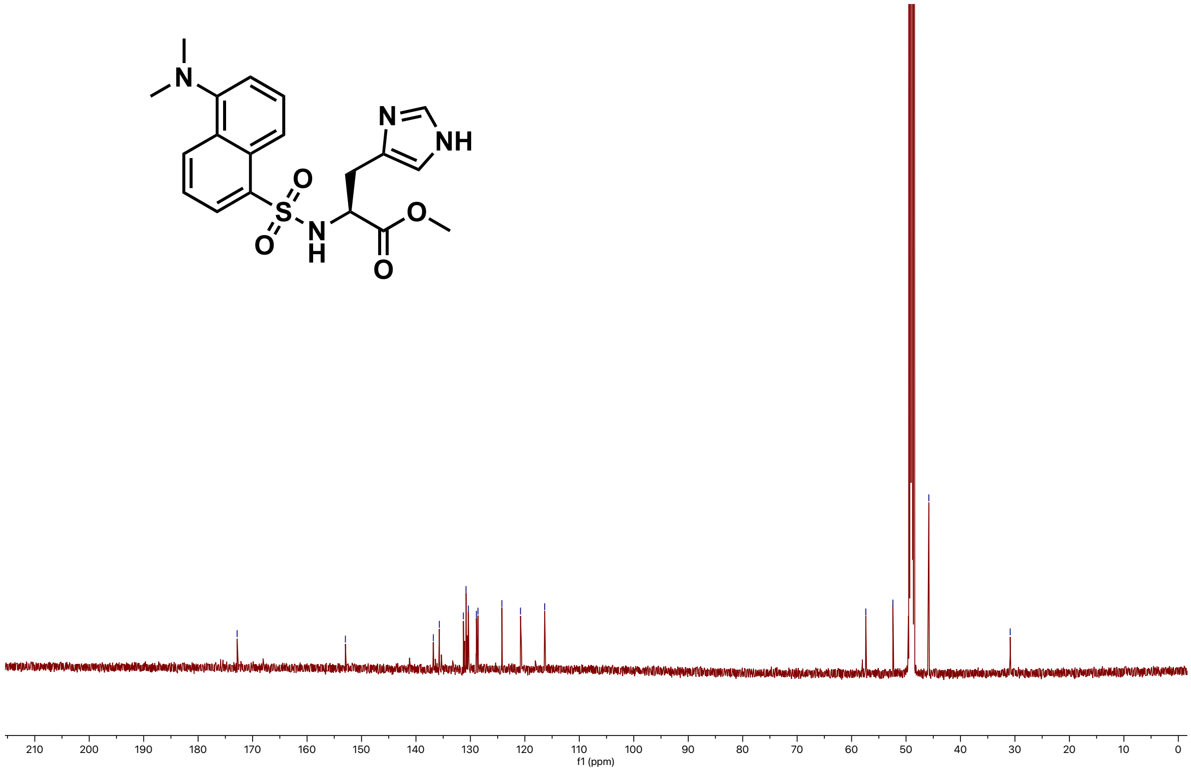

XIV. References

1. Chan, W. C.; White, P. D. Fmoc solid phase peptide synthesis: A practical approach (Oxford Univ. Press, New York, 2000).
