# Supplementary material for: C-Terminal Arginine-Selective Cleavage of Peptides as a Method for Mimicking Carboxypeptidase B
Source: Org Lett. 2023 Aug 16;25(33):6206–10. doi: 10.1021/acs.orglett.3c02418 (PMC10463270; doi:10.1021/acs.orglett.3c02418)
Supplement: Supplementary file 2 — ol3c02418_si_002.zip [file ol3c02418_si_002.zip › FID for Publication/Acquisition Details.rtf]

Spectra were acquired using an Agilent DD2 (400 MHz) spectrometer with a 3-mm He triple resonance (HCN) cyroprobe. Data was analyzed using MestReNova software.1H spectra were measured at 400 MHZ13C spectra were measured at 101 MHz
